# Selective targeting of human TREX1 exonuclease by small molecule inhibitors is mediated by a conformational switch

**DOI:** 10.1093/narmme/ugag033

**Published:** 2026-06-19

**Authors:** Patricia C Hernandez, Rahul Dadabhau Kardile, Ke Shi, Nicholas H Moeller, Joseph A Rollie, Daniel A Harki, Hideki Aihara

**Affiliations:** Department of Biochemistry, Molecular Biology and Biophysics, University of Minnesota, Minneapolis, MN 55455, United States; Institute for Molecular Virology, University of Minnesota, Minneapolis, MN 55455, United States; Masonic Cancer Center, University of Minnesota, Minneapolis, MN 55455, United States; Institute for Molecular Virology, University of Minnesota, Minneapolis, MN 55455, United States; Masonic Cancer Center, University of Minnesota, Minneapolis, MN 55455, United States; Department of Medicinal Chemistry, University of Minnesota, Minneapolis, MN 55455, United States; Department of Biochemistry, Molecular Biology and Biophysics, University of Minnesota, Minneapolis, MN 55455, United States; Institute for Molecular Virology, University of Minnesota, Minneapolis, MN 55455, United States; Masonic Cancer Center, University of Minnesota, Minneapolis, MN 55455, United States; Department of Biochemistry, Molecular Biology and Biophysics, University of Minnesota, Minneapolis, MN 55455, United States; Institute for Molecular Virology, University of Minnesota, Minneapolis, MN 55455, United States; Masonic Cancer Center, University of Minnesota, Minneapolis, MN 55455, United States; Department of Biochemistry, Molecular Biology and Biophysics, University of Minnesota, Minneapolis, MN 55455, United States; Institute for Molecular Virology, University of Minnesota, Minneapolis, MN 55455, United States; Masonic Cancer Center, University of Minnesota, Minneapolis, MN 55455, United States; Institute for Molecular Virology, University of Minnesota, Minneapolis, MN 55455, United States; Masonic Cancer Center, University of Minnesota, Minneapolis, MN 55455, United States; Department of Medicinal Chemistry, University of Minnesota, Minneapolis, MN 55455, United States; Department of Biochemistry, Molecular Biology and Biophysics, University of Minnesota, Minneapolis, MN 55455, United States; Institute for Molecular Virology, University of Minnesota, Minneapolis, MN 55455, United States; Masonic Cancer Center, University of Minnesota, Minneapolis, MN 55455, United States

## Abstract

Three-prime Repair EXonuclease 1 (TREX1) is a 3′-to-5′ exonuclease that degrades cytoplasmic DNA to prevent aberrant innate immune and inflammatory responses. Loss of TREX1 function results in the accumulation of cytosolic DNA and autoimmune disorders in humans, whereas in cancer cells, TREX1 inhibition can promote antitumor immunity triggered by tumor-derived cytoplasmic DNA. Here, we show selective inhibition of human TREX1 (hTREX1) by small molecule inhibitors and reveal their mechanism of target engagement by X-ray crystallography. The structures show that these inhibitors competitively block the binding of the 3′-terminal nucleotide of DNA substrates and make a hydrogen bond with a conserved Glu residue in the active site. Furthermore, binding of the inhibitors stabilizes Tyr129 of hTREX1 in an alternative conformation incompatible with DNA binding, generating a deep hydrophobic pocket that accommodates a key aromatic moiety of the inhibitors. The structural observations provide the basis for high selectivity of these inhibitors for hTREX1 over the closely related hTREX2 or other viral DEDDh-family nucleases. Our studies demonstrate that the conformational flexibility of the hTREX1 active site plays a crucial role in its selective inhibition, informing future design of hTREX1-selective ligands through this structural framework.

## Graphical Abstract

**Graphical Abstract:**
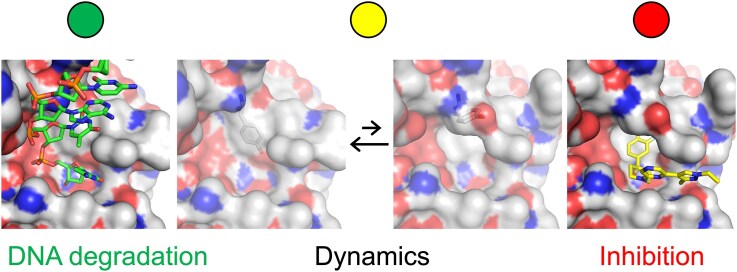

## Introduction

Three-prime Repair EXonuclease 1 (TREX1) is a 3′-to-5′ exonuclease that plays crucial roles in maintaining cellular DNA integrity and regulating immune responses in mammals [1–3]. TREX1 belongs to the DEDDh-family nucleases that share a common protein fold and conserved set of active site residues involved in the coordination of divalent metal ions essential for catalysis [4–8]. This family includes many other cellular and viral 3′-to-5′ exonuclease enzymes, including RNaseT responsible for tRNA maturation, DnaQ involved in DNA polymerase proofreading, deadenylating nuclease (DAN) involved in the regulation of messenger RNA stability, and viral exoribonucleases responsible for the suppression of host immunity and proofreading during RNA synthesis [7, 9–12]. TREX1 consists of the N-terminal DEDDh exonuclease domain and the C-terminal transmembrane domain, which allows it to associate with the endoplasmic reticulum (ER) membrane and digest cytosolic DNA to prevent the activation of cGAS/STING pathway, thereby suppressing inflammatory and innate immune responses [13–15]. A few studies have reported that TREX1 also translocates to the nucleus following DNA damage [16, 17]. Similar observations have been described in the context of granzyme A-mediated cell death, where TREX1 is identified as part of the SET complex [18]. In mice, TREX1 relocalizes to the nucleus following DNA damage or replication stress [3]. A more recent study showed that TREX1 translocates into the nucleus after nuclear envelope rupture and induces DNA damage, which promotes senescence in non-transformed cells and an invasive phenotype in cancer cells [19]. Additionally, TREX1 has been shown to resolve aberrant DNA structures such as 3′-end lesions [20], DNA–protein cross-links [21], and dicentric chromosomes [22].

TREX1 dysfunction causes severe autoimmune diseases in humans, including systemic lupus erythematosus (SLE) [23–27] and Aicardi-Goutières syndrome (AGS) [24, 27–32]. On the other hand, TREX1 upregulation is a key event that facilitates immune evasion of chromosomally unstable cancer cells, and TREX1 inactivation blocks the degradation of tumor-derived DNA and thereby promotes antitumor immunity [33–37]. Additionally, TREX1 is implicated as a hijacked host factor during HIV-1 infection to clear immunostimulatory HIV-1 DNA intermediates [38–41]. Beyond promoting HIV-1 infection through host immunosuppression, studies have shown that TREX1 binds nascent HIV-1 DNA as a protective measure against autointegration [42] and degrades HIV-1 DNA unfit for chromosomal integration [43, 44]. TREX1 activity is also critical for enterovirus replication and entry in intestinal cells [45], along with clearing cytoplasmic mitochondrial DNA produced during influenza A infection [46], broadly indicating how TREX1 is exploited across viral families. In addition, the removal of TREX1 activity enhances CRISPR–Cas9-mediated homologous recombination in gene editing [47]. These studies suggest that small-molecule inhibition of TREX1 could be beneficial in cancer immunotherapies [48–50], antiviral drug development, and genome editing applications.

Recent studies have identified small molecule inhibitors of human TREX1 (hTREX1) and showed that chemical inhibition of hTREX1 upregulates cellular interferon responses and sensitizes tumors to immune checkpoint blockade [45, 50–55]. However, the structural mechanisms of inhibition of hTREX1 by these molecules have not been reported, limiting our understanding of how small molecules can achieve selective inhibition of hTREX1 over other DEDDh-family exonucleases. In this study, we show selective inhibition of hTREX1 by established and novel small molecules through comparative biochemical studies between hTREX1 and the closely related human TREX2 (hTREX2) and viral DEDDh-family exonucleases. Furthermore, we reveal using X-ray crystallography that the highly selective hTREX1 inhibition by these small molecules is achieved by targeting a unique, transient conformation of the hTREX1 active site. Our studies provide a basis for future drug development efforts targeting hTREX1 for novel chemotherapeutic applications.

## Materials and methods

### Chemical syntheses

Previously reported hTREX1 inhibitors were synthesized as described [53]. The synthesis and validation of novel analogues are described in Supporting Information.

### Protein purification

An N-terminal fragment of human TREX1 (residues 1–242), full-length human TREX2, and the C-terminal ExoN fragment of Lassa virus NP (residues 364–569) were expressed with a C-terminal 6xHis tag using the pET-24a vector in *Escherichia coli* strain BL21(DE3) and purified through nickel-affinity chromatography and size-exclusion chromatography (SEC). The full-length SARS-CoV-2 nsp14–nsp10 complex was prepared by co-expressing the two proteins in *E. coli* with a cleavable 6xHis tag on nsp14 and purified as reported previously [56]. The purified proteins in SEC buffer containing 20 mM Tris–HCl, pH 7.4, 0.5 M NaCl, and 5 mM β-mercaptoethanol were concentrated by ultrafiltration, flash frozen in liquid nitrogen in small-volume aliquots, and stored at –80°C.

### Enzyme activity assays

DNA exonuclease activities of hTREX1 and hTREX2 were measured using a fluorescence-based real-time assay. The fluorogenic substrate was assembled from two DNA oligonucleotides: DNA20_v3_5′Cy3 (/5Cy3/GTCATTCTCCTACTCCGCCA) and DNA25_v3_IowaBlackRQ (TTTTTTGGCGGAGTAGGAGAATGAC/3IAbRQSp/). The exoribonuclease activities of SARS-CoV-2 nsp14/nsp10 and Lassa NP-ExoN were measured using a dsRNA substrate assembled from two RNA oligonucleotides /5Cy3/rGrUrCrArUrUrCrUrCrCrUrArArGrArArGrCrUrU and rCrUrArUrCrCrCrCrArUrGrUrGrArUrUrUrUrArCrArArGrCrUrUrCrUrUrArGrGrArGrArArUrGrArC/3IAbRQSp/. The reactions were performed on 52.5 nM Cy3-labeled strand and 150 nM quencher strand in 40 mM Tris–HCl, pH 8.0, 50 mM NaCl, 1.0 mM dithiothreitol (DTT), 1.0 mM MgCl_2_ (for hTREX1, hTREX2, and SARS-CoV-2 nsp14/nsp10) or MnCl_2_ (Lassa NP-ExoN), and 1.0 % dimethyl sulfoxide (DMSO). The nsp14/nsp10 reactions were supplemented with 5% glycerol for enzyme stability. The final enzyme concentrations were 2.3 nM for hTREX1, 19.8 nM for hTREX2, 1.5 nM for nsp14/nsp10, and 31.3 nM for NP-ExoN. Reaction rates were determined by quantifying the maximal slope of the kinetic traces using ICEKAT [57]. Small molecules at various concentrations were incubated with each enzyme for 5 min before the addition of the DNA or RNA substrate to start the reaction. Inhibitory activities of the compounds were evaluated by testing their dose-dependent effect on the reaction rate. The IC_50_ values were determined by plotting the reaction rates against inhibitor concentration and fitting the dose–response data via non-linear regression to the “Variable slope (four parameters)” model in GraphPad Prism.

### X-ray crystallography

Purified hTREX1 at 10.5 mg ml^−1^ (final concentration) in the SEC buffer mixed with 2.0 mM (final concentration) MWAC-1657 and 5% DMSO was crystallized in a 0.2 μl sitting drop formed by combining the protein and reservoir solutions in a 1:1 volume ratio, equilibrated via vapor diffusion against the reservoir solution consisting of 0.1 M sodium acetate trihydrate, pH 4.6, and 2.0 M sodium chloride. Co-crystallization of hTREX1 with MWAC-2515 in a similar fashion using a reservoir solution consisting of 0.1 M Tris, pH 8.3, and 0.8 M lithium sulfate monohydrate yielded microcrystals, which were crushed using Seed Bead (Hampton Research) to generate a seed stock. Diffraction-quality crystals of the hTREX1/MWAC-2515 complex were obtained by the hanging drop vapor diffusion method. 1.5 μl of hTREX1 in the SEC buffer was mixed with 0.1 M MgCl_2_ and 2.0 mM MWAC-2515 such that the final DMSO concentration was 5%. The sample was supplemented with 0.3 μl of microcrystal seeds prepared in the reservoir solution (1:1000 dilution) and then diluted with the reservoir solution to form a 2.8 μl drop, equilibrated against the same reservoir solution used to grow the seed crystals. Crystals were harvested into the respective reservoir solution supplemented with 20% ethylene glycol as a cryo-protectant. X-ray diffraction data were collected at the 17ID1 and 17ID2 beamlines of the National Synchrotron Light Source II (NSLS-II) at Brookhaven National Laboratory. The structures were determined by molecular replacement phasing using PHASER [58] with the human TREX1 crystal structure [8] (PDB: 7TQQ) as the search model. Clear residual electron density corresponding to the bound inhibitors was observed for both protein molecules in the asymmetric unit. The atomic models were built using COOT [59] and refined using PHENIX [60]. A summary of X-ray data collection and model refinement statistics is shown in Table 1. Molecular representation figures were generated using PyMOL (https://www.pymol.org/).

**Table 1.** Summary of X-ray crystallographic data statistics

	hTREX1/MWAC-1657	hTREX1/MWAC-2515
PDB ID	9ZG1	9ZG2
**Data collection**		
Space group	*P*2_1_2_1_2_1_	*P*6_5_
Unit cell dimensions		
*a, b, c* (Å)	82.54, 95.55, 63.57	111.22, 111.22, 96.84
α, β, γ (º)	90, 90, 90	90, 90, 120
Resolution (Å)	32.5–2.36 (2.57–2.36)	19.33–1.94 (2.17–1.94)
*R*_sym_ or *R*_merge_	0.181 (1.45)	0.204 (1.11)
*I / σI*	6.8 (1.5)	6.9 (1.6)
Completeness (%)**	91.1 (56.1)^a^	93.7 (82.3)^b^
Redundancy	4.9 (5.5)	6.6 (5.6)
CC_1/2_	0.995 (0.380)	0.991 (0.613)
**Model refinement**		
Resolution (Å)	32.55–2.36 (2.54–2.36)	19.33–1.94 (2.00–1.94)
No. Reflections	13773 (692)	29345 (1455)
*R*_work_ */ R*_free_	0.189 / 0.250	0.183 / 0.216
No. atoms	3418	3704
Protein	3310	3379
Ligand/ion	62	96
Water	46	229
B-factor (Å^2^)	45.23	31.83
Protein	45.44	31.73
Ligand/ion	38.67	32.25
Water	38.87	33.17
Ramachandran plot		
Favored (%)	97.14	97.92
Allowed (%)	2.38	1.85
Outliers (%)	0.48	0.23
R.m.s. deviations		
Bond lengths (Å)	0.01	0.01
Bond angles (º)	1.10	1.02

Statistics for the highest-resolution shell are shown in parentheses.

** Ellipsoidal resolution cutoff

a a*: 3.28Å , b*: 2.35Å , c*: 2.43 Å

b 0.894a* – 0.447b*: 2.49Å , b*: 2.49Å , c*: 1.91Å

## Results

### Selection and synthesis of hTREX1 inhibitors

**Figure 1.**
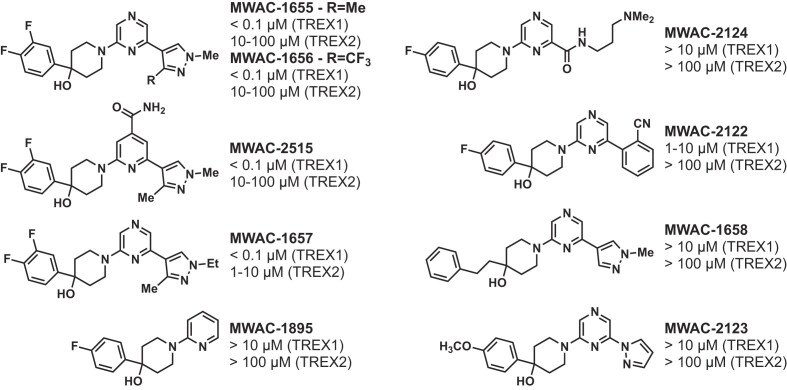
Small molecule hTREX1 inhibitors. Previously reported inhibitors and their associated hTREX1 and hTREX2 biochemical inhibition data (IC_50_) [53].

To investigate the mechanism of chemical inhibition of hTREX1, we selected nine compounds from an extensive patent literature by Constellation Pharmaceuticals towards the development of selective hTREX1 inhibitors [53] (Fig. 1). These compounds (MWAC-1655, 1656, 1657, 1658, 2122, 2123, 2124, 1895, and 2515) share a conserved 4-hydroxypiperidine core that is N-linked to either a pyrazine or pyridine ring with various functional groups. The 4-hydroxypiperidine core is also appended with a 4-aryl or 4-alkyl group of various compositions (Fig. 1). These small molecules were synthesized as previously reported [53]. The inhibitory profiles of these compounds on hTREX1 and hTREX2 that were previously established [53] are provided in Fig. 1.

### hTREX1 Inhibition in a real-time biochemical assay

We employed a fluorescence-based real-time biochemical assay to examine the 3′-to-5′ exonuclease activity of hTREX1 and its inhibition by the inhibitors described above. A fluorogenic double-stranded DNA (dsDNA) substrate used in the assay consists of a 20-nt degradable strand with a Cy3 fluorophore on the 5′ terminus, annealed to a 25-nt complementary strand with a dark quencher (Iowa Black^®^ RQ) attached to the 3′ terminus. hTREX1 progressively degrades the Cy3-labeled strand from the recessed 3′ end, which is eventually released from the quencher strand to liberate the fluorescence signal. The activity of hTREX1 was determined based on the maximal rate of fluorescence intensity increase, which reflects the rate of DNA degradation (Supplementary Fig. S1). The IC_50_ values of each inhibitor were determined by plotting relative enzyme activity against inhibitor concentrations and fitting the dose-response curve to a 4-parameter logistic model.

**Figure 2.**
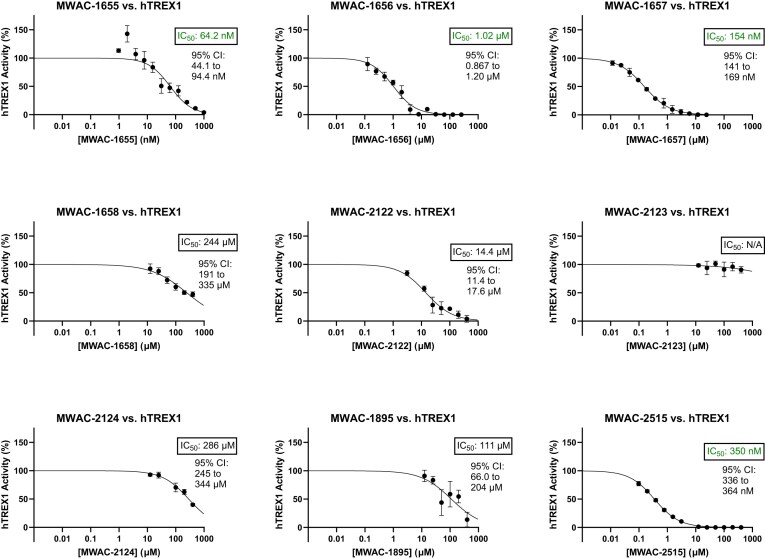
Inhibition of hTREX1 by the small molecules in **Fig. 1**. Relative hTREX1 activities in the presence of various concentrations of the small molecule inhibitors were determined in the real-time fluorescence-based exonuclease assay (representative raw kinetic traces are shown in Supplementary Fig. S1). The reaction rates at varying inhibitor concentrations were extracted using ICEKAT [57] and fitted to the four-parameter logistic model (*R*^2^ of 0.8762, 0.9656, 0.9906, and 0.9978 for MWAC-1655, MWAC-1656, MWAC-1657, and MWAC-2515, respectively). MWAC-1655, 1656, 1657, and 2515 exhibited stronger inhibition with the IC_50_ values of 64.2 nM (95% CI: 44.1–94.4), 1.02 μM (95% CI: 0.867–1.20), 154 nM (95% CI: 141–169), and 350 nM (95% CI: 336–364), respectively. MWAC-2122 inhibited TREX1 weakly, with an IC_50_ of 14.4 μM (95% CI: 11.4–17.6, *R*^2^: 0.9058). IC_50_ values are designated as “N/A” if the calculated IC_50_ exceeds the highest concentration of inhibitor tested. Results are the average of experiments performed in triplicate. The error bars show standard deviations.

The nine compounds showed varying inhibition of the hTREX1 activity consistent with their prior characterization data (Fig. 2). MWAC-1655 and 1657 were the most potent, with IC_50_ values of 64.2 and 154 nM, respectively, consistent with their near-identical structures. Surprisingly, MWAC-1656, which is identical to MWAC-1655 except for a trifluoromethyl group replacing one of the methyl groups on the pyrazole, showed over 15-fold weaker inhibition (IC_50_ = 1.02 μM). MWAC-2515, which has a carboxamide-functionalized pyridine in place of the pyrazine found in MWAC-1655, showed 5-fold weaker hTREX1 inhibition (IC_50_ = 350 nM). MWAC-2122, which has a benzonitrile modification on the pyrazine ring instead of pyrazole, was a weak hTREX1 inhibitor (IC_50_ = 14.4 μM), which is consistent with its prior characterization. The remainder of the compounds showed very weak or no inhibitory activity (Fig. 2). These collective data imply that both sides of the central 4-hydroxypiperidine are important for hTREX1 molecular recognition and subsequent inhibition.

### Selectivity of the hTREX1 inhibitors

**Figure 3.**
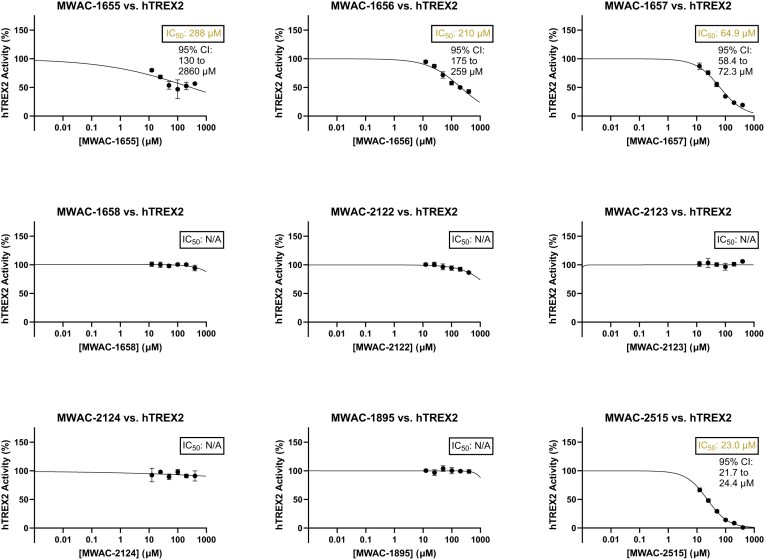
Inhibition of hTREX2 by the small molecules in **Fig. 1**. Relative hTREX2 activities in the presence of various concentrations of the small molecule inhibitors were determined in the real-time fluorescence-based exonuclease assay (representative raw kinetic traces are shown in Supplementary Fig. S1). The data processing and curve fitting were done similarly to hTREX1. The IC_50_ values for MWAC-1655, 1656, 1657, and 2515, which exhibited stronger hTREX1 inhibition (Fig. 2), were 288 μM (95% CI: 130–2860, *R*^2^: 0.7487), 210 μM (95% CI: 175–259, *R*^2^: 0.9480), 64.9 μM (95% CI: 58.4–72.3, *R*^2^: 0.9810), and 23.0 μM (95% CI: 21.7–24.4, *R*^2^: 0.9959), respectively, highlighted in yellow. These IC_50_ values are greater than those against hTREX1 by 4485-fold (MWAC-1655), 206-fold (MWAC-1656), 421-fold (MWAC-1657), and 65.7-fold (MWAC-2515), which demonstrates their high selectivity toward hTREX1. IC_50_ values are designated as “N/A” if the calculated IC_50_ exceeds the highest concentration of inhibitor tested. Results are the average of experiments performed in triplicate. The error bars show standard deviations.

Next, we tested how the nine compounds above inhibit hTREX2, a DEDDh-family DNA exonuclease with a highly homologous catalytic domain to that of hTREX1 (46% amino acid identity; Supplementary Fig. S2). In the real-time biochemical DNA degradation assay used for hTREX1, these compounds showed either very weak or no inhibition of hTREX2 (Fig. 3), consistent with their selectivity toward hTREX1 over hTREX2 reported previously [53] (Fig. 1). Even at the highest concentration tested (400 μM), only one compound, MWAC-2515, achieved complete inhibition of hTREX2 activity (IC_50_ = 23.0 μM). MWAC-1657, which was one of the most potent inhibitors of hTREX1, showed weaker inhibition of hTREX2 (IC_50_ = 64.9 μM). MWAC-1655 and 1656 showed even weaker and more incomplete inhibition of hTREX2 (IC_50_ > 200 μM), whereas the remaining compounds possessed no hTREX2 inhibitory activity. In sum, these inhibitors exhibit selectivity toward hTREX1 over hTREX2 with two orders of magnitude or greater difference in potency.

We also tested whether the nine compounds inhibit DEDDh-family exoribonucleases (ExoN) from SARS-CoV-2 and Lassa virus, which are distantly related to hTREX1 and hTREX2 [56, 61]. In a fluorescence-based real-time biochemical assay modified to monitor RNA degradation, none of the nine compounds showed any inhibition of either of the viral ExoN proteins up to 400 μM (Supplementary Figs S3 and S4). These results further confirm the high selectivity of the TREX1 inhibitors.

### Structural basis of hTREX1 inhibition

**Figure 4.**
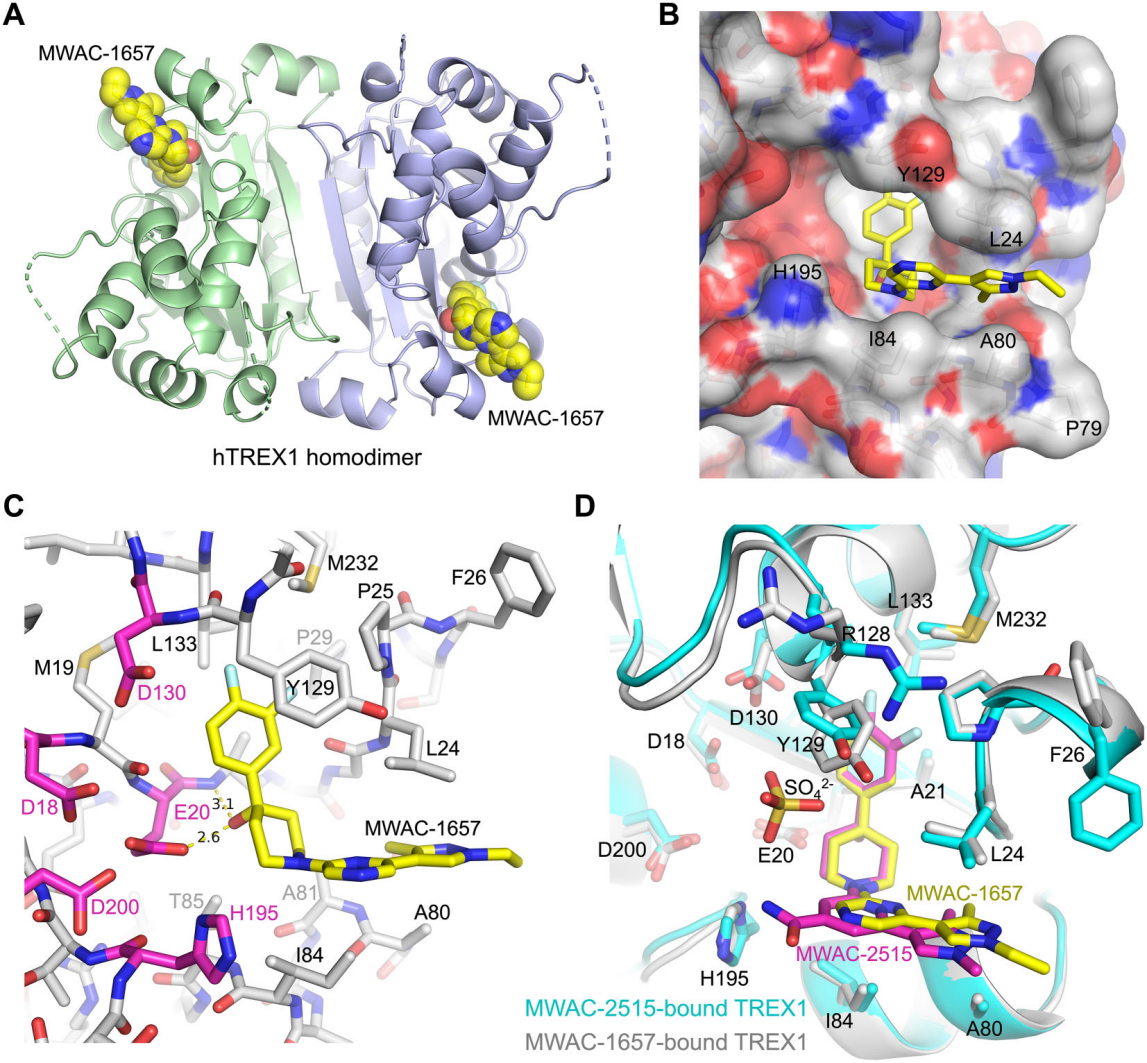
Structural basis of hTREX1 inhibition by MWAC-1657 and 2515. (**A**) Overall structure of the hTREX1 homodimer (cartoon) bound to MWAC-1657 (spheres). (**B**) The molecular surface of hTREX1 with MWAC-1657 bound in the active site pocket. This view is rotated by ∼45 degrees counterclockwise compared to the molecule in the right (slate) in panel (A). (**C**) MWAC-1657 in the hTREX1 active site and the surrounding residues shown in sticks. Yellow dashed lines indicate hydrogen bonds, with distances shown in angstroms. The conserved DEDDh residues are highlighted in magenta. (**D**) A superposition between the MWAC-1657 and 2515-bound crystal structures. The bound sulfate ion is from the MWAC-2515 structure. The views in panels (C) and (D) are slightly rotated about the vertical axis from that in panel (B). The electron density maps for both structures are shown in Supplementary Fig. S5.

To understand the mechanism of hTREX1 inhibition, we determined crystal structures of MWAC-1657 and 2515 in complex with hTREX1. The crystals of the enzyme-inhibitor complexes were obtained by co-crystallization in two different conditions, and the structures were refined to 2.36 and 1.94 Å resolution, respectively. Both structures show a symmetric homodimer of hTREX1, in which the active sites of both protein molecules show a well-defined electron density for the co-crystallized inhibitor (Fig. 4A and Supplementary Fig. S5). The two structures show a common mode of inhibitor binding to the active site. The pyrazine (1657) or pyridine (2515) ring and the attached pyrazole group adopt a planar conformation and are slotted in a hydrophobic groove formed between Leu24, Ala80, and Ile84 (Fig. 4B and D). The directly attached hydroxypiperidine ring in the chair conformation is positioned more deeply within the active site. The hydroxyl group of hydroxypiperidine makes bifurcated hydrogen bonds with the side chain of Glu20, which is one of the Mg^2+^-coordinating residues important for catalysis, and the main chain amide nitrogen atom of the neighboring Ala21 (Fig. 4C). The difluorophenyl moiety, also attached to the fourth position of the piperidine ring, is accommodated in a deep hydrophobic pocket formed by Tyr129, Pro25, Leu133, Ala21, and Leu24, where it makes a face-to-face (1657) or edge-to-face (2515) π-stacking interaction with the Tyr129 side chain (Fig. 4B and D). The unique carboxamide group of MWAC-2515 interacts with His195 and a sulfate ion bound in the active site, which may be responsible for a slight difference in the positioning of the pyrazine/pyridine and pyrazole moieties and the Tyr129 side chain orientation between the two structures (Fig. 4D). The homologated alkyl group on the pyrazole of MWAC-1657 (Me in MWAC-2515 extended to Et) is projected into the solvent.

### Inhibitor versus DNA binding modes

**Figure 5.**
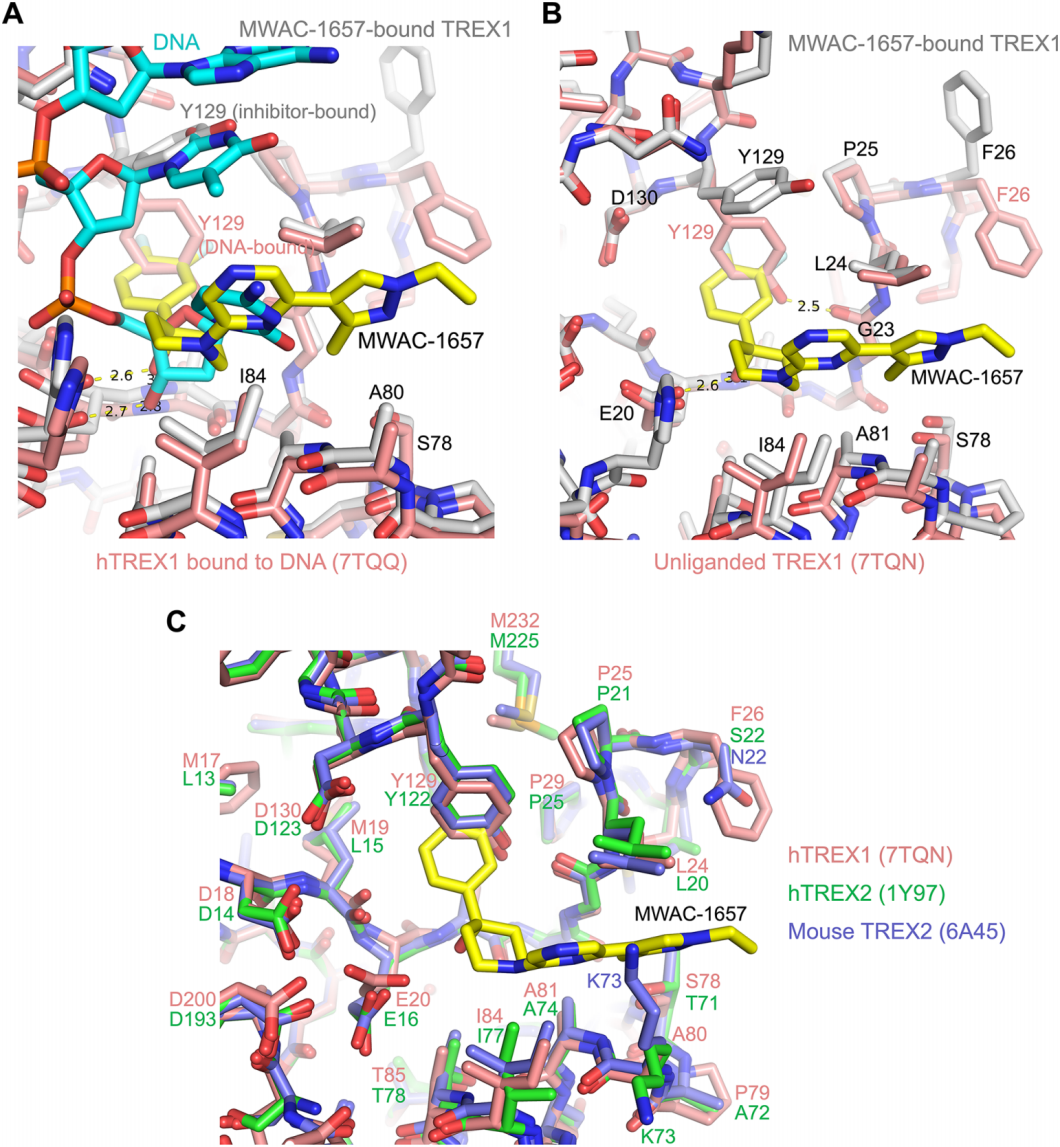
Structural comparisons of closely related three-prime repair exonucleases.(**A**) Superposition between the inhibitor-bound and DNA-bound TREX1 structures, showing DNA mimicry by the inhibitor. (**B**) Superposition between the inhibitor-bound and unliganded TREX1 structures, showing different conformations of Tyr129. (**C**) Superposition between the unliganded TREX1 and TREX2 structures. MWAC-1657 is shown as a reference to highlight that the protein conformation is not compatible with inhibitor-binding.

A superposition between the inhibitor-bound hTREX1 structures described above and the previously reported DNA-bound hTREX1 structure [8] shows that the hydroxypiperidine moiety of the inhibitor overlaps with the deoxyribose moiety of the 3′-terminal nucleotide of DNA (Fig. 5A). The 3′-OH group of DNA is positioned similarly to the hydroxyl group of the 4-hydroxypiperidine moiety of the inhibitors and hydrogen-bonded to the side chain of Glu20 and the main chain of Ala21. Furthermore, the nucleobase of the 3′-terminal DNA nucleotide overlaps with the pyrazine (1657) or pyridine (2515) ring of the inhibitors. Thus, the core of these inhibitors effectively mimics the nucleoside portion of the 3′-terminal nucleotide of the DNA substrate.

The comparison between the inhibitor *vs*. DNA-bound hTREX1 structures also shows that Tyr129 is in markedly different conformations (Fig. 5A). In the DNA-bound structure, the Tyr129 side chain takes the *trans* (χ^1^ = 180°) rotamer and stacks against the deoxyribose moiety of the penultimate nucleotide from the 3′-terminus. By contrast, the Tyr129 side chain flips into the *gauche*^–^ (χ^1^ = –60°) rotamer in the inhibitor-bound structures, which is incompatible with DNA binding due to a steric clash between the Tyr129 side chain and DNA. Importantly, the latter conformation results in the formation of the hydrophobic pocket that accommodates the difluorophenyl moiety of several of the most potent inhibitors. In the crystal structures of hTREX1 without DNA or inhibitor bound, Tyr129 was observed to be in the *trans* rotamer conformation similar to that in the DNA-bound structure [8] (Fig. 5B). The Tyr129 side chain in this conformation is stabilized by a hydrogen bond to the backbone carbonyl group of Gly23, which is likely to represent the lowest energy conformation. However, Tyr129 in this conformation would clash with the difluorophenyl group and thus would not allow the inhibitor to bind. These data suggest that the hydrophobic pocket is formed only transiently in the free enzyme, and the inhibitors specifically target and stabilize this minor conformation of the hTREX1 active site.

### Additional analogues to characterize the structure–activity properties of MWAC-1657

**Figure 6.**
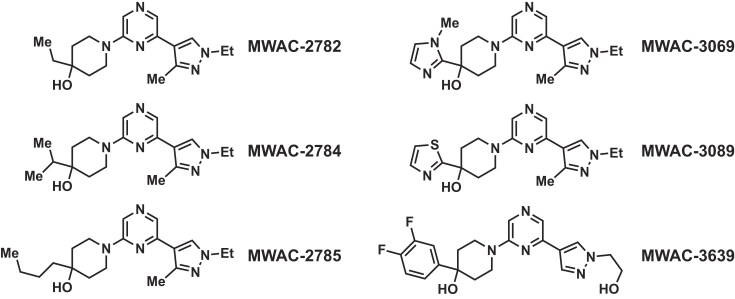
Novel analogues to characterize the structure–activity properties of MWAC-1657. (Left) Three analogues (MWAC-2782, 2784, and 2785) were synthesized to interrogate the necessity for the western (and hydrophobic) aromatic ring. (Right) MWAC-3069 and 3089 retain a smaller aromatic ring in comparison to MWAC-1657. MWAC-3639 was designed to capture additional electrostatic interactions on the ethyl moiety that projects to the solvent, as well as to enhance aqueous solubility.

**Figure 7.**
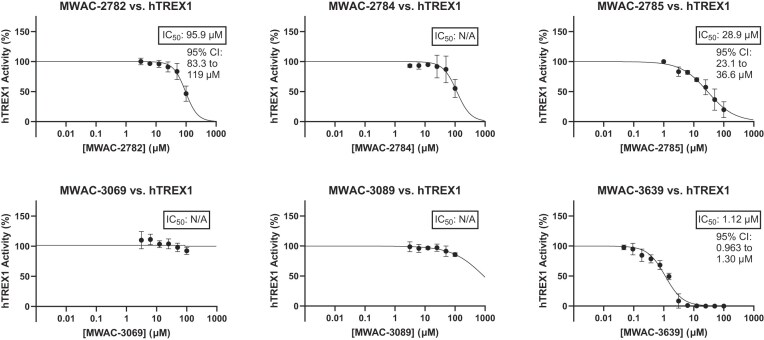
Inhibition of hTREX1 by MWAC-1657 analogues. The IC_50_ value for MWAC-3639 is 1.12 μM (95% CI: 0.963–1.30, *R*^2^: 0.9703). MWAC-2785, which features the longest alkyl chain (*n*-butyl) replacing the critical difluorophenyl moiety, exhibited an IC_50_ of 28.9 μM (95% CI: 23.1–36.6, *R*^2^: 0.911). IC_50_ values are designated as “N/A” if the calculated IC_50_ exceeds the highest concentration of inhibitor tested. Results are the average of experiments performed in triplicate. The error bars show standard deviations.

**Figure 8.**
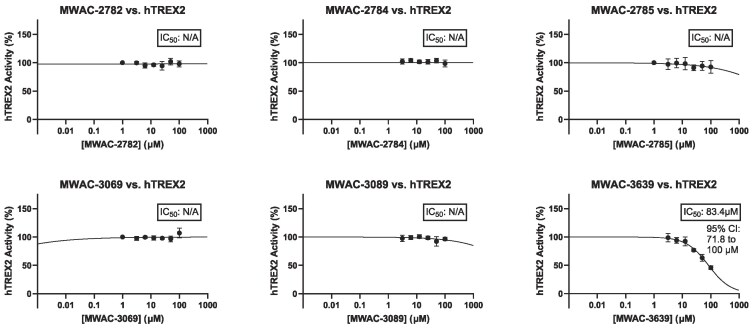
Inhibition of hTREX2 by MWAC-1657 analogues. The IC_50_ for MWAC-3639 against hTREX2 was 83.4 μM (95% CI: 71.8–100, *R*^2^: 0.9438), which is 74.5-fold greater than that against hTREX1. The other compounds showed no inhibition of hTREX2. IC_50_ values are designated as “N/A” if the calculated IC_50_ exceeds the highest concentration of inhibitor tested. Results are the average of experiments performed in triplicate. The error bars show standard deviations.

Given the key interaction made by the difluorophenyl group as revealed by the crystal structures described above, we next tested the overall necessity for this hydrophobic aromatic ring in comparison to hydrophobic alkyl modifications. Accordingly, we synthesized MWAC-2782, 2784, and 2785 that contain an ethyl, isopropyl, or butyl chain, respectively, in place of the difluorophenyl ring found in MWAC-1657 (Fig. 6 and Supporting Information). The potency of these derivatives was assessed by examining dose responses in the real-time assay, as was done for the nine parental compounds. MWAC-2782 and 2784 were only weakly inhibitory on hTREX1, showing partial (∼50%) inhibition even at the highest compound concentration (100 μM) tested (Fig. 7). The dramatic loss of potency compared to MWAC-1657 underscores the importance of the difluorophenyl moiety forming stabilizing interactions in the hydrophobic pocket of hTREX1. Interestingly, MWAC-2785, which has a longer alkyl chain than MWAC-2782 or 2784, partially rescued inhibitory activity with an IC_50_ of 28.9 μM (Fig. 7). This suggests that the butyl group makes some van der Waals or hydrophobic interaction with the hTREX1 active site, despite lacking the key aromatic group. None of these three derivatives showed inhibition of hTREX2, suggesting that their hTREX1 inhibition, albeit weak, is specific (Fig. 8).

To further explore the necessity of a western difluorophenyl ring, we synthesized MWAC-3069 and MWAC-3089, which have *N*-methylimidazole and thiazole ring systems, respectively, in place of the difluorobenzene (Fig. 6 and Supporting Information). Perhaps not unexpectedly, MWAC-3069 and 3089 were much less potent than MWAC-1657 (IC_50_ > 100 μM), which underscores the necessity for the larger arene system (Fig. 7). Notably, MWAC-2123 from the parental set of compounds showed no appreciable inhibition of hTREX1 even at the highest concentration tested (400 μM), despite being more structurally similar to MWAC-1657 with a 4-methoxy group in place of 3,4-difluoro substitutions (Fig. 2). These data, taken together, underscore the sensitivity of the western portion of the molecule to modification. Based on the observation that the ethyl group on the eastern pyrazole ring of MWAC-1657 projects into the solvent (Fig. 4), we synthesized MWAC-3639 as part of an effort to capture additional stabilizing electrostatic interactions with hTREX1 and/or to impart improved physicochemical properties onto the molecule, such as enhanced aqueous solubility (Fig. 6 and Supplementary Data). Evaluation of MWAC-3639 for hTREX1 inhibition revealed an IC_50_ of 1.12 μM, which is about 17-fold less potent than its congener (Fig. 7). In sum, these collective data point towards key hydrophobic interactions on both termini of MWAC-1657 as being involved in favorable stabilizing interactions with hTREX1.

## Discussion

Human TREX1 and TREX2 share a highly similar active site. Most hTREX1 residues that make direct contact with the inhibitors in our structures are conserved between the two enzymes, including Tyr129 of hTREX1, which is responsible for the key conformational change (Supplementary Fig. S2). This raises a question about how the inhibitors achieve high selectivity for hTREX1 over hTREX2 (Figs 2 and 3). A superposition of the two structures shows that there are, however, differences between the two proteins that may underlie better binding of the inhibitors to hTREX1 (Fig. 5C). For instance, Ala80, adjacent to the hydrophobic groove that accommodates the terminal pyrazole moiety of most inhibitors (Figs 1 and 4), is replaced by Lys73 in hTREX2. The Lys73 side chain on the hTREX2 surface is likely to be flexible, and it could potentially project into the hydrophobic groove as observed in the mouse TREX2 crystal structure [62] (blue in Fig. 5C, PDB ID: 6A45) to interfere with the binding of the inhibitors or DNA. In the exonuclease activity assays, an approximately 10-fold higher concentration of hTREX2 was required to achieve an equivalent level of activity to hTREX1, possibly reflecting a weaker DNA-binding affinity of hTREX2. Additionally, Arg128 of hTREX1 neighboring Tyr129 is replaced by Asp121 in hTREX2. Our hTREX1–MWAC-2515 structure suggests that the Arg128 side chain may play a role in facilitating the conformational change of Tyr129 by interacting with its side chain hydroxyl group (Supplementary Fig. S5B), to potentially compensate for the loss of a hydrogen bond to Gly23 observed in the unliganded hTREX1 structure (Fig. 5B). Other differences could also modulate protein structural dynamics to affect the binding of the inhibitors, such as Met19 deep in the hydrophobic pocket of hTREX1 being replaced by Leu15 in hTREX2, and Phe26/Ser27/Gln28 from a short 3_10_ helix of hTREX1 flanking the hydrophobic groove being replaced by Ser22/Val23/Glu24 in hTREX2 (Fig. 5C and Supplementary Fig. S2). Future mutagenesis studies are required to test these hypotheses and understand the roles of unique hTREX1 residues in inhibitor binding.

Despite the hTREX1–inhibitor structures showing limited hydrogen bond interactions (represented by MWAC-1657 and 2515), they are highly selective for hTREX1 and do not appreciably inhibit the distantly related viral ExoN enzymes. The bifurcated hydrogen bonds between the hydroxyl group of the 4-hydroxypiperidine core and Glu20 from the conserved DEDDh motif and the main chain amide nitrogen atom of the neighboring Ala21 anchor the inhibitor to the core of the hTREX1 active site, whereas the rest of the enzyme-inhibitor contact is mostly hydrophobic interactions or stacking of aromatic moieties (Fig. 4B–D). The inhibitors fit in a narrow cleft that accommodates the 3′-terminal base of a DNA substrate on one side, and in a deep pocket generated by the conformational change of Tyr129 on the other side. Hypothetical placement of MWAC-1657 in the active site of SARS-CoV-2 and LASV ExoN based on structural superposition of the conserved DEDDh catalytic residues suggests that not only the inhibitor would make a steric clash, but the active sites of these enzymes are also not compatible with taking an alternative conformation like hTREX1 to accommodate the difluorophenyl moiety (Supplementary Fig. S6). The insertion of the difluorophenyl group of the inhibitors into the transiently formed deep pocket is essential for potency, as shown by the dramatic loss of inhibitory activities of the derivatives lacking this moiety (Fig. 6; see MWAC-2782, 2784, 2785, 3069, 3089). The newly synthesized analogues do not provide an improvement in potency; rather, they support our hypothesis that the mechanism of inhibition relies heavily on the western aryl ring (difluorobenzene) and confirm our structural model. Thus, the potency and selectivity of these compounds against hTREX1 are likely to be attributable to shape complementarity with distinct active site features, as well as specifically targeting a transient and unique conformation of the active site. Although it may not be surprising that almost no inhibition was observed on the viral ExoNs, given their dsRNA preference and differences in active site landscape, these biochemical data are important in demonstrating that the mechanistic reasoning behind compound inhibition is not explained by the conserved DEDDh motif.

In conclusion, this work provides a detailed molecular understanding of how small molecule inhibitors engage the hTREX1 active site and bind in a unique conformation distinct from previous TREX1 structures. Our work highlights the importance of protein dynamics in the selective targeting of an enzyme and may help further hTREX1 inhibitor development.

## Supplementary Material

ugag033_Supplemental_Files

## Data Availability

Atomic coordinates and structure factors have been deposited in the RCSB Protein Data Bank with accession DOIs: https://doi.org/10.2210/pdb9zg1/pdb and https://doi.org/10.2210/pdb9zg2/pdb for the hTREX1/MWAC-1657 and hTREX1/MWAC2515 structures, respectively. All other data are available from the authors upon request.
