# Supplementary material for: Selective targeting of human TREX1 exonuclease by small molecule inhibitors is mediated by a conformational switch
Source: NAR Mol Med. 2026 Jun 19;3(3):ugag033. doi: 10.1093/narmme/ugag033 (PMC13335479; doi:10.1093/narmme/ugag033)
Supplement: ugag033_Supplemental_Files [file ugag033_supplemental_files.zip › 250626214715_TREX1_Chemistry_SI_Final.pdf]

Supporting Information

Selective targeting of human TREX1 exonuclease by small molecule inhibitors is mediated by a conformational switch

Patricia C. Hernandez[1,2,3], Rahul Dadabhau Kardile[2,3,4], Ke Shi[1,2,3], Nicholas H. Moeller[1,2,3], Joseph A. Rollie[1,2,3], Daniel A. Harki[2,3,4,*], Hideki Aihara[1,2,3,*]

[1] Department of Biochemistry, Molecular Biology and Biophysics, University of Minnesota, Minneapolis, Minnesota, USA, 55455
[2] Institute for Molecular Virology, University of Minnesota, Minneapolis, Minnesota, USA, 55455
[3] Masonic Cancer Center, University of Minnesota, Minneapolis, Minnesota, USA, 55455
[4] Department of Medicinal Chemistry, University of Minnesota, Minneapolis, Minnesota, USA, 55455

* Corresponding authors: aihar001@umn.edu, daharki@umn.edu

Table of Contents

Synthesis Procedures and Characterization……..…………………………………….……….. S2

NMR Spectra (^{1}H, ^{13}C & ^{19}F)……………….……....…………………………….……..…….. S14

HPLC Purity Traces…………...…………………………………………………….………. S58

Reference..……………………………………...……………………………………… S72

Synthesis procedures and compound characterization:

Unless otherwise noted, chemicals were used as received and reactions were stirred with a Teflon-coated stir bar. Glassware was not dried unless noted. Tetrahydrofuran (THF) was dried by passage over a column of activated alumina using a solvent purification system (MBraun). 2,6-dichloropyrazine was purchased from Ambeed, 1,4-dioxa-8-azaspiro[4.5]decane was purchased from Oakwood Chemicals, and the boronic acid pinacol esters were purchased from Enamine. Reactions were monitored using thin layer chromatography with EMD Chemicals Silica Gel 60 F_{254} glass plates (250 µm thickness) and visualized with UV irradiation at either 254 nm or 365 nm. Flash chromatography was performed with a Teledyne-Isco CombiFlash NextGen instrument, equipped with both a UV-Vis detector and an ELSD using Redisep Rf High Performance silica gel columns (Teledyne-Isco). ^{1}H NMR (500 MHz), ^{13}C NMR (126 MHz) and ^{19}F NMR (471 MHz) were collected on a Bruker Advance NMR spectrometer at room temperature. NMR chemical shifts (δ) are in parts per million (ppm) and recorded relative to TMS (0.05% v/v, δ = 0.0) or residual solvent signal (δ = 7.26 ppm for $CDCl_3$, 2.50 ppm for DMSO-d_6) for ^{1}H NMR and the solvent signal for ^{13}C NMR (δ = 77.0 for $CDCl_3$, or 49.0 ppm for CD_3OD). HRMS data were collected on a Velos Pro (Thermo) mass analyzer at 240,000 resolution with electrospray ionization in positive mode. Reverse-phase purification was performed on a ACCQ Prep HP 150 instrument [column: Agilent Zorbax SB-C18 (21.2 x 250 mm, 7 µm pore)]. Mobile phase A = H_2O, mobile phase B = CH_3CN, flow rate = 30 mL/min. Elution: 90:10 A:B for 0-2 min, gradient to 30:70 A:B from 2-22 min, gradient to 5:95 A:B from 22 min to 28 min, isocratic at 5:95 A:B from 28-30 min.

4-ethyl-1-(6-(1-ethyl-3-methyl-1*H*-pyrazol-4-yl)pyrazin-2-yl)piperidin-4-ol (MWAC-2782):

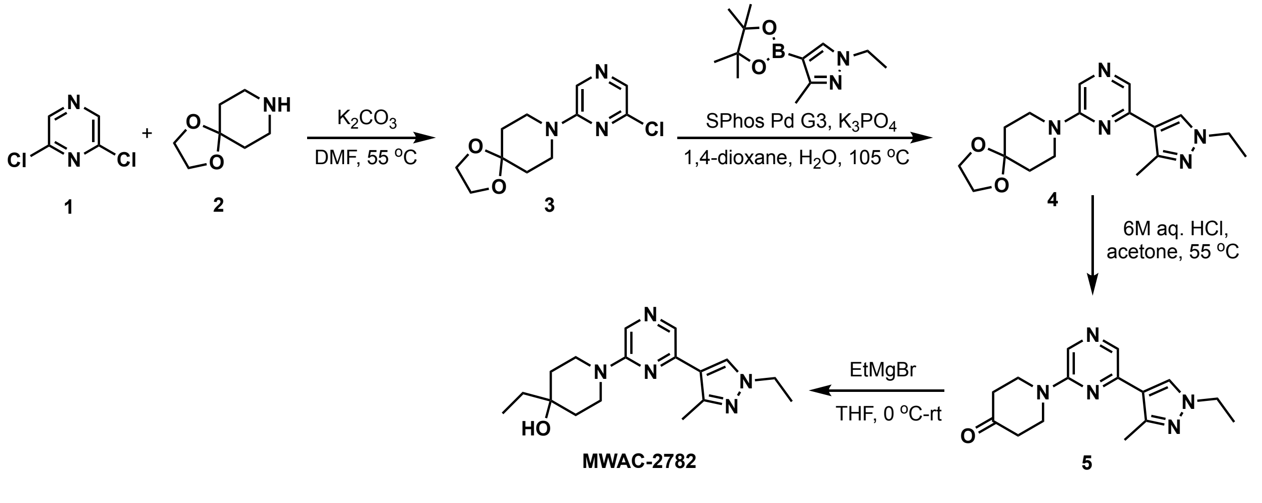

8-(6-chloropyrazin-2-yl)-1,4-dioxa-8-azaspiro[4.5]decane (3): 2,6-dichloropyrazine (5.00 g, 33.6 mmol), 1,4-dioxa-8-azaspiro[4.5]decane (5.29 g, 33.9 mmol), and potassium carbonate (9.28 g, 67.1 mmol) were combined in DMF (15 mL) and heated to 55 °C for 16h. The reaction was cooled to room temperature and diluted with water (10 mL) and extracted with EtOAc (3 x 20 mL). The combined organic

layer was washed with brine (5 mL) and dried over Na_2SO_4, filtered, and concentrated *in vacuo*. The crude product was purified by silica gel flash chromatography (0-100% EtOAc in hexanes). The desired product **3** was obtained as off-white solid (7.22 g, 84%).

^{1}H NMR (500 MHz, $CDCl_3$): δ 7.99 (s, 1H), 7.69 (s, 1H), 3.99 (s, 4H), 3.74 – 3.71 (m, 4H), 1.77 – 1.75 (m, 4H).

^{13}C NMR (126 MHz, $CDCl_3$): δ 153.7, 146.6, 130.2, 127.8, 106.9, 64.5, 42.8, 34.4.

HRMS: $C_{11}H_{15}ClN_3O_2$ Calc′d $[M+H]^+$: 256.0853; Found: 256.0847.

8-(6-(1-ethyl-3-methyl-1*H*-pyrazol-4-yl)pyrazin-2-yl)-1,4-dioxa-8-azaspiro[4.5]decane (4): 8-(6-chloropyrazin-2-yl)-1,4-dioxa-8-azaspiro[4.5]decane (**3**, 1.00 g, 3.91 mmol), 1-ethyl-3-methyl-4-(4,4,5,5-tetramethyl-1,3,2-dioxaborolan-2-yl)-1*H*-pyrazole (1.02 g, 4.30 mmol), (2-dicyclohexylphosphino-2′,6′-dimethoxybiphenyl) [2-(2′-amino-1,1′-biphenyl)]palladium(II) methanesulfonate (S-Phos Pd G3, 91.5 mg, 117 μmol), and potassium phosphate tribasic (2.49 g, 11.7 mmol) were combined in 1,4-dioxane (10 mL) and water (2 mL) and the mixture was heated to 105 °C under N_2 for 16 hours. The reaction was diluted with EtOAc (30 mL) and filtered through a pad of celite eluting with EtOAc. The eluent was concentrated *in vacuo*, and the residue was purified by silica gel chromatography (0-100% EtOAc in hexanes) to afford **4** as white solid (1.10 g, 85%).

^{1}H NMR (500 MHz, $CDCl_3$): δ 8.01 (s, 1H), 7.95 (s, 1H), 7.78 (s, 1H), 4.13 (q, *J* = 7.3 Hz, 2H), 4.00 (s, 4H), 3.77 – 3.75 (m, 4H), 2.54 (s, 3H), 1.80 – 1.78 (m, 4H), 1.50 (t, *J* = 7.3 Hz, 3H).

^{13}C NMR (126 MHz, $CDCl_3$): δ 153.6, 147.2, 145.9, 129.4, 128.6, 127.0, 117.7, 107.3, 64.4, 46.9, 43.0, 34.3, 15.5, 14.5.

HRMS: $C_{17}H_{24}N_5O_2$ Calc′d $[M+H]^+$: 330.1930; Found: 330.1924.

1-(6-(1-ethyl-3-methyl-1*H*-pyrazol-4-yl)pyrazin-2-yl)piperidin-4-one (5): 8-(6-(1-ethyl-3-methyl-1*H*-pyrazol-4-yl)pyrazin-2-yl)-1,4-dioxa-8-azaspiro[4.5]decane (**4**) (1.10 g, 3.34 mmol) was dissolved in acetone (15 mL) and aqueous HCl (6 M; 3.34 mL, 20.0 mmol) was added. The reaction was stirred at 55 °C for 36 h. The solvent was then evaporated under vacuum, and the crude reaction was neutralized with aqueous NaOH (6 M NaOH in water) and extracted with EtOAc (3 x 30 mL). The combined organic layer was washed with brine (20 mL), dried over Na_2SO_4 and purified by silica gel chromatography (0-100% EtOAc in hexanes) to afford **5** (801 mg, 84%) as off-white solid.

^{1}H NMR (500 MHz, CDCl$_3$): δ 8.08 (s, 1H), 7.99 (s, 1H), 7.78 (s, 1H), 4.11 (q, J = 7.3 Hz, 2H), 3.96 (t, J = 6.0 Hz, 4H), 2.54 – 2.52 (m, 7H), 1.48 (t, J = 7.3 Hz, 3H).

^{13}C NMR (126 MHz, CDCl$_3$): δ 207.4, 152.8, 147.1, 146.1, 130.1, 128.5, 126.4, 117.2, 46.8, 43.9, 15.4, 14.4.

HRMS: $C_{15}H_{20}N_5O$ Calc'd [M+H]$^+$: 286.1668; Found: 286.1661.

4-ethyl-1-(6-(1-ethyl-3-methyl-1*H*-pyrazol-4-yl)pyrazin-2-yl)piperidin-4-ol (MWAC-2782): 1-(6-(1-ethyl-3-methyl-1*H*-pyrazol-4-yl)pyrazin-2-yl)piperidin-4-one (**5**) (100 mg, 0.35 mmol) was dissolved in anhydrous THF (2 mL) and cooled to 0 °C. Ethyl magnesium bromide (1M in THF, 701 µL, 0.70 mmol) was added dropwise under N_2. The reaction was allowed to warm to room temperature and stirred for 16 hours. After completion, reaction mixture was cooled to 0 °C (ice bath) and carefully quenched with saturated aqueous NH_4Cl solution (5 mL) then extracted with EtOAc (3 x 20 mL). The combined organic layer was washed with brine (10 mL), dried over Na_2SO_4 and concentrated *in vacuo*. The crude product was purified by silica gel chromatography (0-10% MeOH in DCM) and then reverse phase C18 column chromatography (10-95% MeCN in water). The fractions containing product were combined and lyophilized to afford **MWAC-2782** as white solid (16.1 mg, 15%).

^{1}H NMR (500 MHz, CDCl$_3$): δ 7.97 (s, 1H), 7.93 (s, 1H), 7.78 (s, 1H), 4.13 (q, J = 7.3 Hz, 2H), 4.08 (dt, J = 13.0 Hz, 3.4 Hz, 2H), 3.41 – 3.36 (m, 2H), 2.54 (s, 3H), 1.66 – 1.64 (m, 4H), 1.54 – 1.48 (m, 6H), 0.94 (t, J = 7.5 Hz, 3H).

^{13}C NMR (126 MHz, CDCl$_3$): δ 154.0, 147.2, 145.9, 129.0, 128.6, 127.0, 117.8, 70.0, 46.9, 40.9, 35.8, 35.4, 33.0, 29.7, 15.5, 14.5, 7.0.

HRMS: $C_{17}H_{26}N_5O$ Calc'd [M+H]$^+$: 316.2137; Found: 316.2123.

1-(6-(1-ethyl-3-methyl-1*H*-pyrazol-4-yl)pyrazin-2-yl)-4-isopropylpiperidin-4-ol (MWAC-2784):

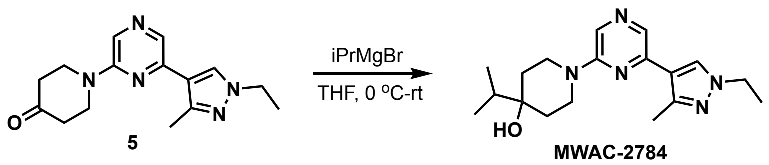

1-(6-(1-ethyl-3-methyl-1*H*-pyrazol-4-yl)pyrazin-2-yl)piperidin-4-one (**5**) (50.0 mg, 0.175 mmol) was dissolved in anhydrous THF (3 mL) and cooled to 0 °C. Isopropylmagnesium bromide (0.75 M in THF, 467 µL, 0.350 mmol) was added dropwise under nitrogen. The reaction was allowed to warm to room temperature and stirred for 16 hours. The reaction mixture was then cooled to 0 °C (ice bath) and carefully

quenched with saturated aqueous NH_4Cl (5 mL) then extracted with EtOAc (3 x 20 mL). The combined organic layer was washed with brine (10 mL), dried over Na_2SO_4 and concentrated *in vacuo*. The crude product was purified by silica gel chromatography (0-10% MeOH in DCM) and then reverse phase C18 column chromatography (10-95% MeCN in water). The fractions containing product were combined and lyophilized to afford **MWAC-2784** as white solid (9.40 mg, 16%).

^{1}H NMR (500 MHz, $CDCl_3$): δ 7.97 (s, 1H), 7.93 (s, 1H), 7.79 (s, 1H), 4.20 (dt, *J* = 13.1 Hz, 3.4 Hz, 2H), 4.14 (q, *J* = 7.3 Hz, 2H), 3.32 – 3.27 (m, 2H), 2.55 (s, 3H), 1.71 – 1.61 (m, 5H), 1.50 (t, *J* = 7.5 Hz, 3H), 0.94 (d, *J* = 7.0 Hz, 6H).

^{13}C NMR (126 MHz, $CDCl_3$): δ 154.1, 147.3, 146.2, 128.6, 128.4, 126.4, 117.7, 71.8, 46.9, 40.8, 38.0, 33.5, 16.4, 15.5, 14.5.

HRMS: $C_{18}H_{28}N_5O$ Calc'd $[M+H]^+$: 330.2294; Found: 330.2280.

4-butyl-1-(6-(1-ethyl-3-methyl-1*H*-pyrazol-4-yl)pyrazin-2-yl)piperidin-4-ol (MWAC-2785):

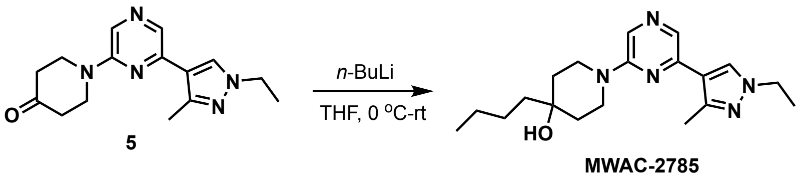

1-(6-(1-ethyl-3-methyl-1*H*-pyrazol-4-yl)pyrazin-2-yl)piperidin-4-one (**5**) (50.0 mg, 0.175 mmol) was dissolved in anhydrous THF (3 mL) and cooled to 0 °C. *n*-Butyllithium (2.5 M in hexane, 105 µL, 0.263 mmol) was added dropwise under nitrogen. The reaction was allowed to warm to room temperature and stirred for 16 hours. The reaction mixture was then cooled to 0 °C (ice bath) and carefully quenched with saturated aqueous NH_4Cl solution (5 mL) then extracted with EtOAc (3 x 20 mL). The combined organic layer was washed with brine (10 mL), dried over Na_2SO_4 and concentrated *in vacuo*. Crude product was purified by silica gel chromatography (0-10% MeOH in DCM) and further purified by reverse phase C18 column chromatography (10-95% MeCN in water). The fractions containing product were combined and lyophilized to afford **MWAC-2785** as white solid (15.1 mg, 25%).

^{1}H NMR (500 MHz, $CDCl_3$): δ 7.98 (s, 1H), 7.93 (s, 1H), 7.78 (s, 1H), 4.13 (q, *J* = 7.3 Hz, 2H), 4.09 – 4.05 (m, 2H), 3.42 – 3.36 (m, 2H), 2.54 (s, 3H), 1.68 – 1.65 (m, 4H), 1.51 – 1.48 (m, 5H), 1.38 – 1.30 (m, 5H), 0.91 (t, *J* = 6.9 Hz, 3H).

^{13}C NMR (126 MHz, $CDCl_3$): δ 154.1, 147.2, 146.1, 128.7, 128.6, 126.7, 117.7, 69.9, 46.9, 42.8, 40.9, 36.2, 24.9, 23.1, 15.5, 14.5, 14.0.

HRMS: $C_{19}H_{30}N_5O$ Calc'd $[M+H]^+$: 344.2450; Found: 344.2437.

1-(6-(1-ethyl-3-methyl-1*H*-pyrazol-4-yl)pyrazin-2-yl)-4-(1-methyl-1*H*-imidazol-2-yl)piperidin-4-ol (MAWC-3069):

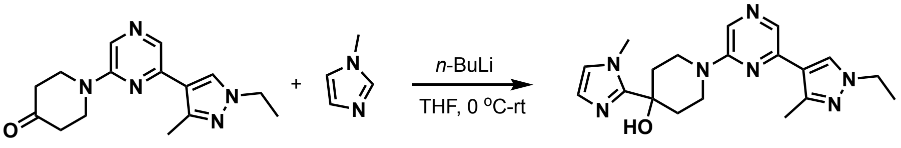

5

MWAC-3069

1-methyl-1*H*-imidazole (86.3 mg, 1.05 mmol) was dissolved in anhydrous THF (1 mL) and cooled to -78 °C. *n*-Butyllithium (2.5 M in hexane, 449 μL, 1.12 mmol) was added dropwise and the reaction mixture was stirred for 20 min at the same temperature. Then, anhydrous THF (1 mL) solution of 1-(6-(1-ethyl-3-methyl-1*H*-pyrazol-4-yl)pyrazin-2-yl)piperidin-4-one (**5**, 100 mg, 0.350 mmol) was added dropwise under nitrogen. The reaction was allowed to warm to room temperature and stirred for 4 hours. The reaction mixture was then cooled to 0 °C and carefully quenched with saturated aqueous NH_4Cl solution (5 mL) then extracted with ethyl acetate (3 x 50 mL). The combined organic layer was washed with brine (30 mL), dried over Na_2SO_4 and concentrated *in vacuo*. Crude product was purified by silica gel chromatography (0-10% MeOH in DCM) and further purified by reverse phase C18 column chromatography (10-95% MeCN in water). The fractions containing product were combined and lyophilized to afford **MWAC-3069** as pale yellow solid (9.30 mg, 7%).

^{1}H NMR (500 MHz, $CDCl_3$): δ 7.97 (s, 1H), 7.92 (s, 1H), 7.78 (s, 1H), 6.87 (s, 1H), 6.83 (s, 1H), 4.16 – 4.11 (m, 4H), 3.87 (s, 3H), 3.56 (t, *J* = 11.3 Hz, 2H), 2.53 (s, 3H), 2.32 – 2.25 (m, 2H), 2.02 – 2.01 (m, 2H), 1.50 (t, *J* = 7.3 Hz, 3H).

^{13}C NMR (126 MHz, $CDCl_3$): δ 154.0, 150.6, 147.2, 146.1, 129.1, 128.6, 126.6, 125.4, 123.3, 117.7, 69.7, 46.9, 40.7, 35.5, 35.0, 15.5, 14.5.

HRMS: $C_{19}H_{26}N_7O$ Calc'd $[M+H]^+$: 368.2199; Found: 368.2190.

1-(6-(1-ethyl-3-methyl-1*H*-pyrazol-4-yl)pyrazin-2-yl)-4-(thiazol-2-yl)piperidin-4-ol (MWAC-3089):

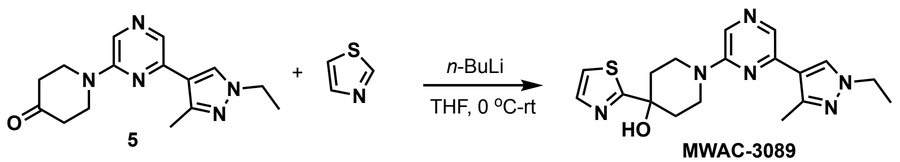

5

MWAC-3089

1,3-thiazole (89.5 mg, 1.05 mmol) was dissolved in anhydrous THF (1 mL) and cooled to -78 °C. *n*-Butyllithium (2.5 M in hexane, 449 µL, 1.12 mmol) was added dropwise and the reaction mixture was stirred for 20 min at the same temperature. Then, an anhydrous THF (1 mL) solution of 1-(6-(1-ethyl-3-methyl-1*H*-pyrazol-4-yl)pyrazin-2-yl)piperidin-4-one (**5**, 100 mg, 0.350 mmol) was added dropwise under nitrogen. The reaction was allowed to warm to room temperature and stirred for 4 hours. The reaction mixture was then cooled to 0 °C (ice bath) and carefully quenched with saturated aqueous NH_4Cl solution (5 mL) then extracted with EtOAc (3 x 50 mL). The combined organic layer was washed with brine (30 mL), dried over Na_2SO_4 and concentrated *in vacuo*. Crude product was purified by silica gel chromatography (0-10% MeOH in DCM) and further purified by reverse phase C18 column chromatography (10-95% MeCN in water). The fractions containing product were combined and lyophilized to afford **MWAC-3089** as off-white solid (15.2 mg, 11%).

^{1}H NMR (500 MHz, $CDCl_3$): δ 8.01 (s, 1H), 7.97 (s, 1H), 7.79 (s, 1H), 7.72 (d, *J* = 3.1 Hz, 1H), 7.30 (d, *J* = 3.2 Hz, 1H), 4.28 (d, *J* = 13.3 Hz, 2H), 4.14 (q, *J* = 7.3 Hz, 2H), 3.57 – 3.51 (m, 2H), 3.4 (bs, 1H), 2.54 (s, 3H), 2.26 (td, *J* = 13.3 Hz, 4.6 Hz, 2H), 2.02 – 1.99 (m, 2H), 1.50 (t, *J* = 7.3 Hz, 3H).

^{13}C NMR (126 MHz, $CDCl_3$): δ 177.8, 153.9, 147.3, 146.2, 142.2, 129.0, 128.7, 126.6, 119.3, 117.7, 72.6, 46.9, 40.9, 37.8, 15.5, 14.5.

HRMS: $C_{18}H_{23}N_6OS$ Calc'd $[M+H]^+$: 371.1654; Found: 371.1639.

4-(3,4-difluorophenyl)-1-(6-(1-(2-hydroxyethyl)-1*H*-pyrazol-4-yl)pyrazin-2-yl)piperidin-4-ol (MWAC-3639):

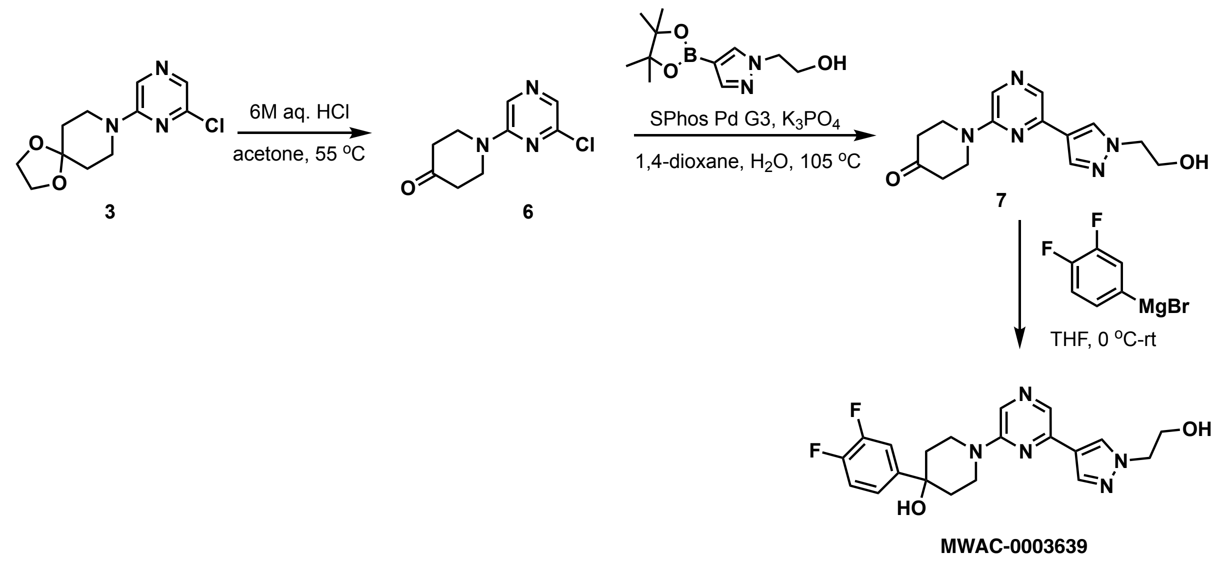

1-(6-chloropyrazin-2-yl)piperidin-4-one (6): 8-(6-chloropyrazin-2-yl)-1,4-dioxa-8-azaspiro[4.5]decane (**3**) (3.25 g, 12.7 mmol) was dissolved in acetone (20 mL) and aqueous HCl solution (6 M HCl in water, 12.7 mL, 76.3 mmol) was added. The reaction was stirred at 55 °C for 36 h. The solvent was then evaporated, and the crude reaction was neutralized with aqueous NaOH (6 M NaOH in water) and extracted with EtOAc (2 x 100 mL). The combined organic layer was washed with brine (50 mL), dried over Na_2SO_4 and purified by silica gel chromatography (0-100% EtOAc in hexanes) to afford **6** (2.25 g, 83%) as an off-white solid.

^{1}H NMR (500 MHz, $CDCl_3$): δ 8.07 (s, 1H), 7.89 (s, 1H), 3.96 (t, *J* = 6.2 Hz, 4H), 2.58 (t, *J* = 5.7 Hz, 4H).

^{13}C NMR (126 MHz, $CDCl_3$): δ 206.7, 153.1, 146.7, 131.4, 127.5, 43.5, 40.2.

1-(6-(1-(2-hydroxyethyl)-1*H*-pyrazol-4-yl)pyrazin-2-yl)piperidin-4-one (7): 1-(6-chloropyrazin-2-yl)piperidin-4-one (**6**): (500 mg, 2.36 mmol), 2-(4-(4,4,5,5-tetramethyl-1,3,2-dioxaborolan-2-yl)-1*H*-pyrazol-1-yl)ethan-1-ol (562 mg, 4.72 mmol), (2-dicyclohexylphosphino-2′,6′-dimethoxybiphenyl) [2-(2′-amino-1,1′-biphenyl)]palladium(II) methanesulfonate (S-Phos Pd G3, 55.3 mg, 70.9 μmol), and potassium phosphate tribasic (1.50 g, 7.09 mmol) were combined in 1,4-dioxane (5 mL) and water (1 mL) and the resulting mixture was heated to 105 °C under an atmosphere of nitrogen for 16 hour. The reaction was diluted with EtOAc (30 mL) and filtered through a pad of celite eluting with EtOAc. The eluent was concentrated, and the residue was purified by silica gel chromatography (0-100% EtOAc in hexanes) to afford **7** (550 mg, 81%) as off-white solid.

^{1}H NMR (500 MHz, $CDCl_3$): δ 8.12 (s,1H), 8.04 (s,1H), 8.00 (s, 1H), 7.97 (s, 1H), 4.31 (t, *J* = 4.9 Hz, 2H), 4.05 (t, *J* = 4.6 Hz, 2H), 4.00 (t, *J* = 6.1H, 4H), 2.98 (bs, 1H), 2.57 (t, *J* = 6.1 Hz, 4H).

^{13}C NMR (126 MHz, $CDCl_3$): δ 207.5, 153.0, 144.6, 138.1, 129.5, 129.1, 127.5, 120.7, 61.7, 54.2, 43.8, 40.5.

HRMS: $C_{15}H_{18}N_5O_2$ Calc′d $[M+H]^+$: 288.1460; Found: 288.1453.

4-(3,4-difluorophenyl)-1-(6-(1-(2-hydroxyethyl)-1*H*-pyrazol-4-yl)pyrazin-2-yl)piperidin-4-ol (MWAC-3639): 1-(6-(1-(2-hydroxyethyl)-1*H*-pyrazol-4-yl)pyrazin-2-yl)piperidin-4-one (**7**) (212 mg, 0.73 mmol) was dissolved in anhydrous THF (3 mL) and cooled to 0 °C. Then, freshly prepared (3,4-difluorophenyl)magnesium bromide (~2 M in THF, 737 μL, 1.47 mmol) was added dropwise under nitrogen. The reaction was allowed to warm to room temperature and stirred overnight. The reaction mixture was then cooled to 0 °C and carefully quenched with saturated aqueous NH_4Cl solution (5 mL) then extracted with ethyl acetate (3 x 20 mL). The combined organic layer was washed with brine (10

mL), dried over Na_2SO_4 and concentrated *in vacuo*. Crude product was purified by silica gel chromatography (0-10% MeOH in DCM) and further purified by reverse phase C18 column chromatography (10-95% MeCN in water). The fractions containing product were combined and lyophilized to afford **MWAC-3639** as white solid (25.5 mg, 9%).

^{1}H NMR (500 MHz, $CDCl_3$): δ 8.02 (s, 1H), 7.98 (s, 1H), 7.96 (s, 1H), 7.36 – 7.32 (m, 1H), 7.18 – 7.11 (m, 2H), 4.35 – 4.29 (m, 4H), 4.05 – 4.03 (m, 2H), 3.41 (t, *J* = 12.3 Hz, 2H), 2.08 (td, *J* = 13.4 Hz, 4.6 Hz, 2H), 1.86 – 1.84 (m, 4H).

^{13}C NMR (126 MHz, $CDCl_3$): δ 154.1, 149.8 (dd, J_{C-F} = 248.2 Hz, 100.8 Hz),145.1, 144.6, 138.1, 129.2, 128.1, 127.4, 120.9, 120.5 (d, J_{C-F} = 6.3 Hz), 117.2 (d, J_{C-F} = 63.9 Hz), 114.1 (d, J_{C-F} = 68.6 Hz), 71.2, 61.7, 54.2, 40.7, 37.7.

^{19}F NMR (471 MHz, $CDCl_3$): δ -136.9, -139.8.

HRMS: $C_{20}H_{22}F_2N_5O_2$ Calc'd $[M+H]^+$: 402.1742; Found: 402.1726.

4-(3,4-difluorophenyl)-1-(6-(1,3-dimethyl-1*H*-pyrazol-4-yl)pyrazin-2-yl)piperidin-4-ol (MWAC-1655):

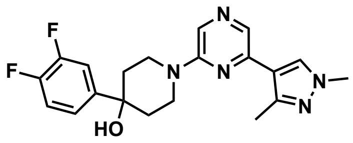

MWAC-1655

Prepared according to a reported procedure[1]. Mass spectrometry data is consistent with reported data.

^{1}H NMR (500 MHz, $CDCl_3$): δ 8.02 (s,1H), 7.99 (s,1H), 7.77 (s, 1H), 7.36 – 7.31 (m, 1H), 7.20 – 7.11 (m, 2H), 4.32 (dt, *J* = 13.2 Hz, 2.2 Hz, 2H), 3.88 (s, 3H), 3.43 (td, *J* = 13.5 Hz, 2.5 Hz, 2H), 2.55 (s, 3H), 2.10 (td, *J* = 13.4 Hz, 4.8 Hz, 2H), 1.86 – 1.83 (m, 2H), 1.76 (s, 1H).

^{13}C NMR (126 MHz, $CDCl_3$): δ 153.9, 146.6 (d, J_{C-F} = 197.8 Hz), 145.2, 130.3, 129.5, 127.1, 120.5 (d, J_{C-F} = 3.7 Hz), 120.4 (d, J_{C-F} = 3.9 Hz), 118.0, 117.1 (d, J_{C-F} = 17.6 Hz), 114.15 (d, J_{C-F} = 18.9 Hz), 71.3, 40.9, 38.8, 37.6, 14.5.

^{19}F NMR (471 MHz, $CDCl_3$): δ -137.0, -139.9.

HRMS: $C_{20}H_{22}F_2N_5O$ Calc'd $[M+H]^+$: 386.1792 ; Found: 386.1778.

4-(3,4-difluorophenyl)-1-(6-(1-methyl-3-(trifluoromethyl)-1*H*-pyrazol-4-yl)pyrazin-2-yl)piperidin-4-ol (MWAC-1656):

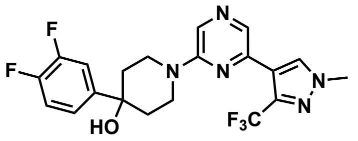

MWAC-1656

Prepared according to a reported procedure[1]. Mass spectrometry data is consistent with reported data.

^{1}H NMR (500 MHz, $CDCl_3$): δ 8.08 (s,1H), 8.03 (s,1H), 7.84 (s, 1H), 7.35 – 7.31 (m, 1H), 7.19 – 7.10 (m, 2H), 4.36 – 4.33 (m, 2H), 4.00 (s, 3H), 3.43 (td, *J* = 12.0 Hz, 1.3 Hz, 2H), 2.10 (td, *J* = 13.4 Hz, 4.6 Hz, 2H), 1.85 – 1.831 (m, 3H).

^{13}C NMR (126 MHz, $CDCl_3$): δ 153.7, 149.8 (dd, J_{C-F} = 248.2 Hz, 100.8 Hz), 145.1, 142.6, 139.2 (d, J_{C-F} = 37.8 Hz), 131.7, 129.8, 128.9, 121.2 (q, J_{C-F} = 269.6 Hz), 120.5 (d, J_{C-F} = 2.5 Hz), 120.4 (d, J_{C-F} = 3.7 Hz), 119.6, 117.1 (d, *J* = 17.1 Hz), 114.0 (d, *J* = 18.9 Hz), 71.3, 40.7, 39.7, 37.6.

^{19}F NMR (471 MHz, $CDCl_3$): δ -60.0, -137.0, -139.9.

HRMS: $C_{20}H_{19}F_5N_5O$ Calc'd $[M+H]^+$: 440.1510; Found: 440.1493.

4-(3,4-difluorophenyl)-1-(6-(1-ethyl-3-methyl-1*H*-pyrazol-4-yl)pyrazin-2-yl)piperidin-4-ol (MWAC-1657):

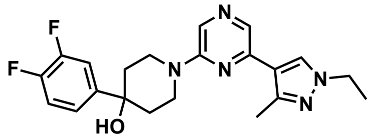

MWAC-1657

Prepared according to a reported procedure[1]. Mass spectrometry data is consistent with reported data.

^{1}H NMR (500 MHz, $CDCl_3$): δ 8.03 (s,1H), 7.98 (s,1H), 7.80 (s, 1H), 7.36 – 7.32 (m, 1H), 7.20 – 7.11 (m, 2H), 4.32 (dt, *J* = 15.0 Hz, 2.3 Hz, 2H), 4.14 (q, *J* = 7.3 Hz, 2H), 3.43 (td, *J* = 13.0 Hz, 2.6 Hz, 2H), 2.56 (s, 3H), 2.10 (td, *J* = 13.4 Hz, 4.7 Hz, 2H), 1.86 – 1.83 (m, 2H), 1.75 (s, 1H), 1.51 (t, *J* = 7.3 Hz, 3H).

^{13}C NMR (126 MHz, $CDCl_3$): δ 153.9, 147.2, 146.0, 129.5, 128.6, 127.0, 120.5 (d, *J* = 3.7 Hz), 120.4 (d, *J* = 5.0 Hz), 117.7, 117.1 (d, *J* = 17.1 Hz), 114.0 (d, *J* = 18.2 Hz), 71.3, 46.9, 40.9, 37.7, 15.5, 14.5.

^{19}F NMR (471 MHz, $CDCl_3$): δ -137.0, -139.9.

HRMS: $C_{21}H_{24}F_2N_5O$ Calc′d $[M+H]^+$: 400.1949; Found: 400.1935.

1-(6-(1-methyl-1*H*-pyrazol-4-yl)pyrazin-2-yl)-4-phenethylpiperidin-4-ol (MWAC-1658):

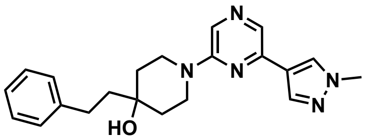

MWAC-1658

Prepared according to a reported procedure[1]. Mass spectrometry data is consistent with reported data.

^{1}H NMR (500 MHz, $CDCl_3$): δ 8.02 (s,1H), 7.96 (s,1H), 7.95 (s, 1H), 7.87 (s, 1H), 7.30 – 7.27 (m, 2H), 7.21 – 7.17 (m, 3H), 4.12 (dt, *J* = 12.7 Hz, 3.4 Hz, 2H), 3.96 (s, 3H), 3.43 – 3.38 (m, 2H), 2.77 – 2.73 (m, 2H), 1.86 – 1.82 (m, 2H), 1.75 – 1.74 (m, 4H), 1.29 (s, 1H).

^{13}C NMR (126 MHz, $CDCl_3$): δ 154.1, 144.5, 142.0, 137.6, 128.9, 128.5, 128.4, 128.3, 127.8, 125.9, 121.3, 66.9, 44.9, 40.7, 39.1, 36.3, 29.2.

HRMS: $C_{21}H_{26}N_5O$ Calc′d $[M+H]^+$: 364.2137; Found: 364.2125.

4-(4-fluorophenyl)-1-(pyridin-2-yl)piperidin-4-ol (MWAC-1895):

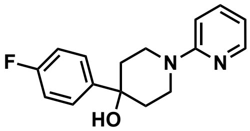

MWAC-1895

Prepared according to a reported procedure[1]. Mass spectrometry data is consistent with reported data.

^{1}H NMR (500 MHz, $CDCl_3$): δ 8.20 – 8.19 (m,1H), 7.50 – 7.45 (m,3H), 7.03 (t, *J* = 9.0 Hz, 2H), 6.72 (d, *J* = 8.5 Hz, 1H), 6.62- 6.60 (m, 1H), 4.24 – 4.20 (m, 2H), 3.36 (td, *J* = 13.0 Hz, 2.5 Hz, 2H), 2.12 (td, *J* = 13.0 Hz, 4.5 Hz, 2H), 1.86 – 1.82 (m, 2H), 1.61 (s, 1H).

^{13}C NMR (126 MHz, $CDCl_3$): δ 161.9 (d, $J_{C\text{-}F}$ = 245.7 Hz), 159.3, 148.0, 143.9 (d, $J_{C\text{-}F}$ = 2.5 Hz), 137.5, 126.2 (d, $J_{C\text{-}F}$ = 8.8 Hz), 115.1 (d, $J_{C\text{-}F}$ = 21.4 Hz), 112.9, 107.2, 71.5, 41.6, 37.9.

^{19}F NMR (471 MHz, $CDCl_3$): δ -115.8.

HRMS: $C_{21}H_{24}F_2N_5O$ Calc′d $[M+H]^+$: 273.1403; Found: 273.1392.

2-(6-(4-(4-fluorophenyl)-4-hydroxypiperidin-1-yl)pyrazin-2-yl)benzonitrile (MWAC-2122):

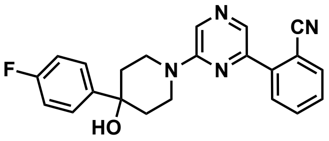

MWAC-2122

Prepared according to a reported procedure[1]. Mass spectrometry data is consistent with reported data.

^{1}H NMR (500 MHz, CDCl$_3$): δ 8.21 (s, 1H), 8.17 (s,1H), 7.84 – 7.79 (m, 2H), 7.67 (t, *J* = 7.7 Hz, 1H), 7.54 – 7.46 (m, 3H), 7.03 (t, *J* = 8.7 Hz, 2H), 4.50 – 4.44 (m, 2H), 3.55 (td, *J* = 13.0 Hz, 2.3 Hz, 2H), 2.16 (td, *J* = 13.4 Hz, 4.7 Hz, 2H), 1.92 – 1.76 (m, 3H).

^{13}C NMR (126 MHz, CDCl$_3$): δ 162.2 (d, J_{C-F} = 245.9 Hz), 153.5, 148.0, 143.6 (d, J_{C-F} = 3.0 Hz), 140.7, 135.1, 132.6, 129.7, 129.7, 129.4, 129.0 (d, J_{C-F} = 13.7 Hz), 126.38, 126.32, 118.9, 115.2 (d, J_{C-F} = 21.5 Hz), 111.5, 71.4, 40.9, 37.9.

^{19}F NMR (471 MHz, CDCl$_3$): δ -115.5.

HRMS: $C_{22}H_{20}FN_4O$ Calc'd [M+H]$^+$: 375.1621; Found: 375.1607.

1-(6-(1*H*-pyrazol-1-yl)pyrazin-2-yl)-4-(4-methoxyphenyl)piperidin-4-ol (MWAC-2123):

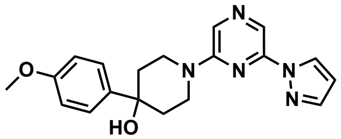

MWAC-2123

Prepared according to a reported procedure[1]. Mass spectrometry data is consistent with reported data.

^{1}H NMR (500 MHz, CDCl$_3$): δ 8.51 (s, 1H), 8.43 (d, *J* = 2.5 Hz, 1H), 8.06 (s, 1H), 7.74 (s,1H), 7.42 (d, J = 8.8 Hz, 2H), 6.89 (d, *J* = 8.8 Hz, 2H), 6.46 – 6.45 (m, 1H), 4.30 – 4.27 (m, 2H), 3.80 (s, 3H), 3.51 (td, *J* = 12.9 Hz, 2.6 Hz, 2H), 2.11 (td, *J* = 13.43, 4.7 Hz, 2H), 1.93 – 1.90 (m, 2H), 1.82 (bs, 1H).

^{13}C NMR (126 MHz, CDCl$_3$): δ 158.8, 152.8, 145.2, 142.4, 139.8, 127.5, 127.1, 125.6, 121.0, 113.8, 107.7, 71.1, 55.3, 41.0, 37.7.

HRMS: $C_{19}H_{22}N_5O_2$ Calc'd [M+H]$^+$: 352.1773; Found: 352.1761.

N-(3-(dimethylamino)propyl)-6-(4-(4-fluorophenyl)-4-hydroxypiperidin-1-yl)pyrazine-2-carboxamide (MWAC- 2124):

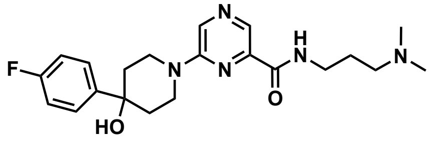

MWAC-2124

Prepared according to a reported procedure[1]. Mass spectrometry data is consistent with reported data.

^{1}H NMR (500 MHz, $CDCl_3$): δ 8.58 (s, 1H), 8.28 (s,1H), 7.48 – 7.45 (m, 2H), 7.05 – 7.01 (m, 2H), 4.30 – 4.28 (m, 2H), 3.53 – 3.45 (m, 4H), 2.67 (bs, 1H), 2.40 (t, J = 6.3 Hz, 2H), 2.19 (s, 6H), 2.08 (td, J = 13.4 Hz, 4.7 Hz, 2H), 1.91 – 1.89 (m, 2H), 1.76 – 1.71 (m, 2H).

^{13}C NMR (126 MHz, $CDCl_3$): δ 164.0, 161.9 (d, $J_{C\text{-}F}$ = 246.5 Hz), 152.8, 143.7 (d, $J_{C\text{-}F}$ = 3.1 Hz), 141.5, 133.4, 131.3, 126.2 (d, $J_{C\text{-}F}$ = 8.1 Hz), 115.2 (d, $J_{C\text{-}F}$ = 21.5 Hz), 70.9, 58.8, 45.6, 40.7, 39.1, 37.7, 26.3.

^{19}F NMR (471 MHz, $CDCl_3$): δ -115.4.

HRMS: $C_{21}H_{29}FN_5O_2$ Calc′d $[M+H]^+$: 402.2305; Found: 402.2291.

2-(4-(3,4-difluorophenyl)-4-hydroxypiperidin-1-yl)-6-(1,3-dimethyl-1*H*-pyrazol-4-yl)isonicotinamide (MWAC-2515):

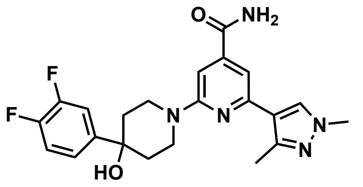

MWAC-2515

Prepared according to a reported procedure[1]. Mass spectrometry data is consistent with reported data.

^{1}H NMR (500 MHz, DMSO-d_6): δ 8.12 (s, 1H), 8.08 (s, 1H), 7.53 – 7.48 (m, 2H), 7.37 – 7.28 (m, 2H), 7.18 (s, 1H), 7.02 (s, 1H), 5.30 (s, 1H), 4.29 (d J = 12.6 Hz, 2H), 3.78 (s, 3H), 3.28 (t, J = 12.6 Hz, 2H), 2.43 (s, 3H), (td, J = 13.1 Hz, 4.3 Hz, 2H), 1.69 – 1.67 (m, 2H).

^{13}C NMR (126 MHz, CD_3OD): δ 171.6, 160.7, 153.3, 150.7 (dd, $J_{C\text{-}F}$ = 245.7, 124.7 Hz), 148.3, 148.2 (d, $J_{C\text{-}F}$ = 3.7 Hz), 144.7, 132.5, 122.3 (d, $J_{C\text{-}F}$ = 3.7 Hz), 122.2 (d, $J_{C\text{-}F}$ = 2.5 Hz), 121.8, 117.8 (d, $J_{C\text{-}F}$ = 17.6 Hz), 107.9, 103.0, 72.0, 42.7, 38.6, 38.5, 14.6.

^{19}F NMR (471 MHz, DMSO- d_6): δ -139.0, -142.3.

HRMS: $C_{22}H_{24}F_2N_5O_2$ Calc′d $[M+H]^+$: 428.1898; Found: 428.1880.

NMR Spectra

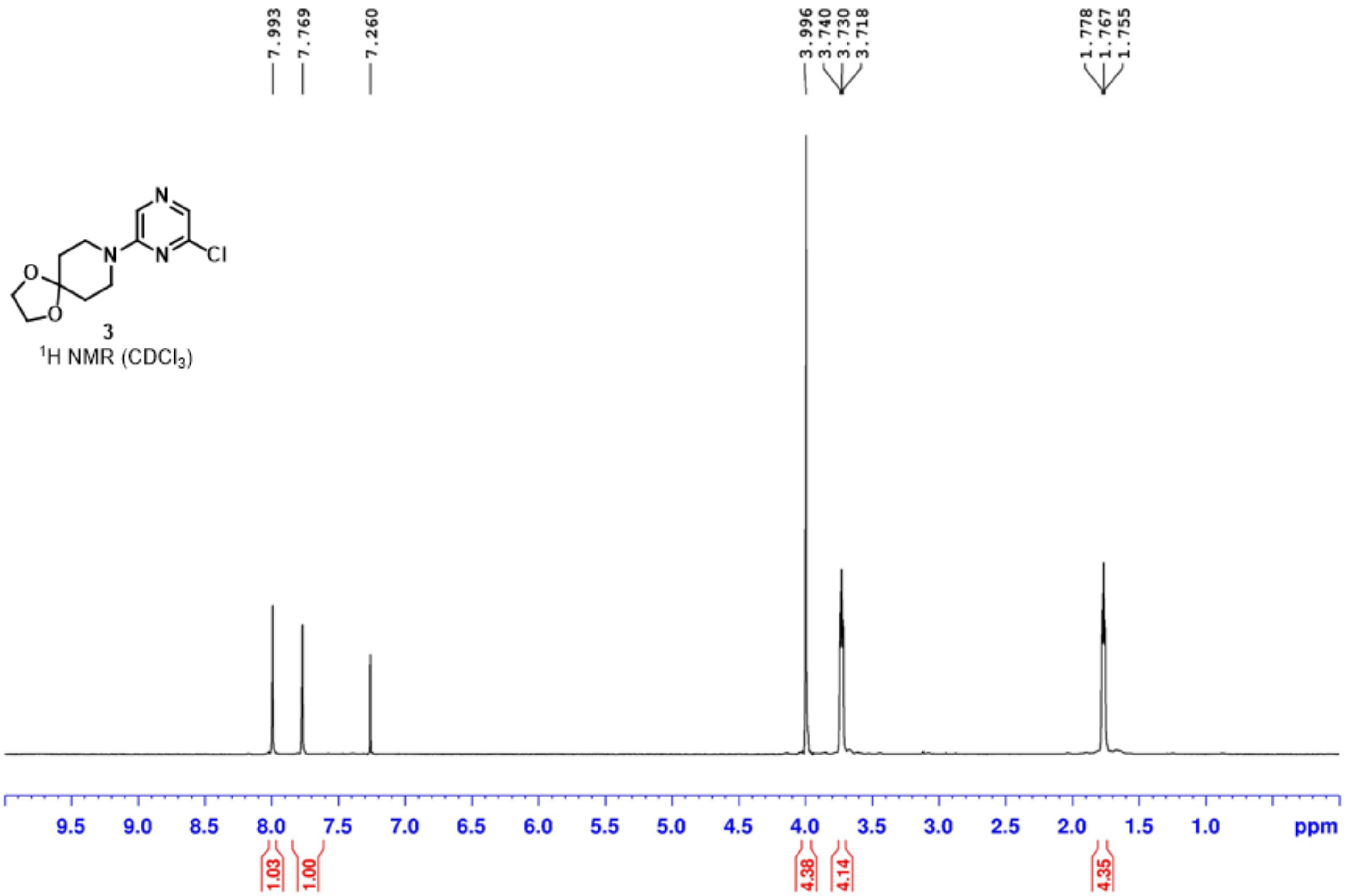

7.993
7.769
7.260
3.996
3.740
3.730
3.718
1.778
1.767
1.755
N
N
Cl
O
O
N
3
^{1}H NMR ($CDCl_3$)
9.5 9.0 8.5 8.0 7.5 7.0 6.5 6.0 5.5 5.0 4.5 4.0 3.5 3.0 2.5 2.0 1.5 1.0 ppm
1.03
1.00
4.38
4.14
4.35

3

^{13}C NMR ($CDCl_3$)

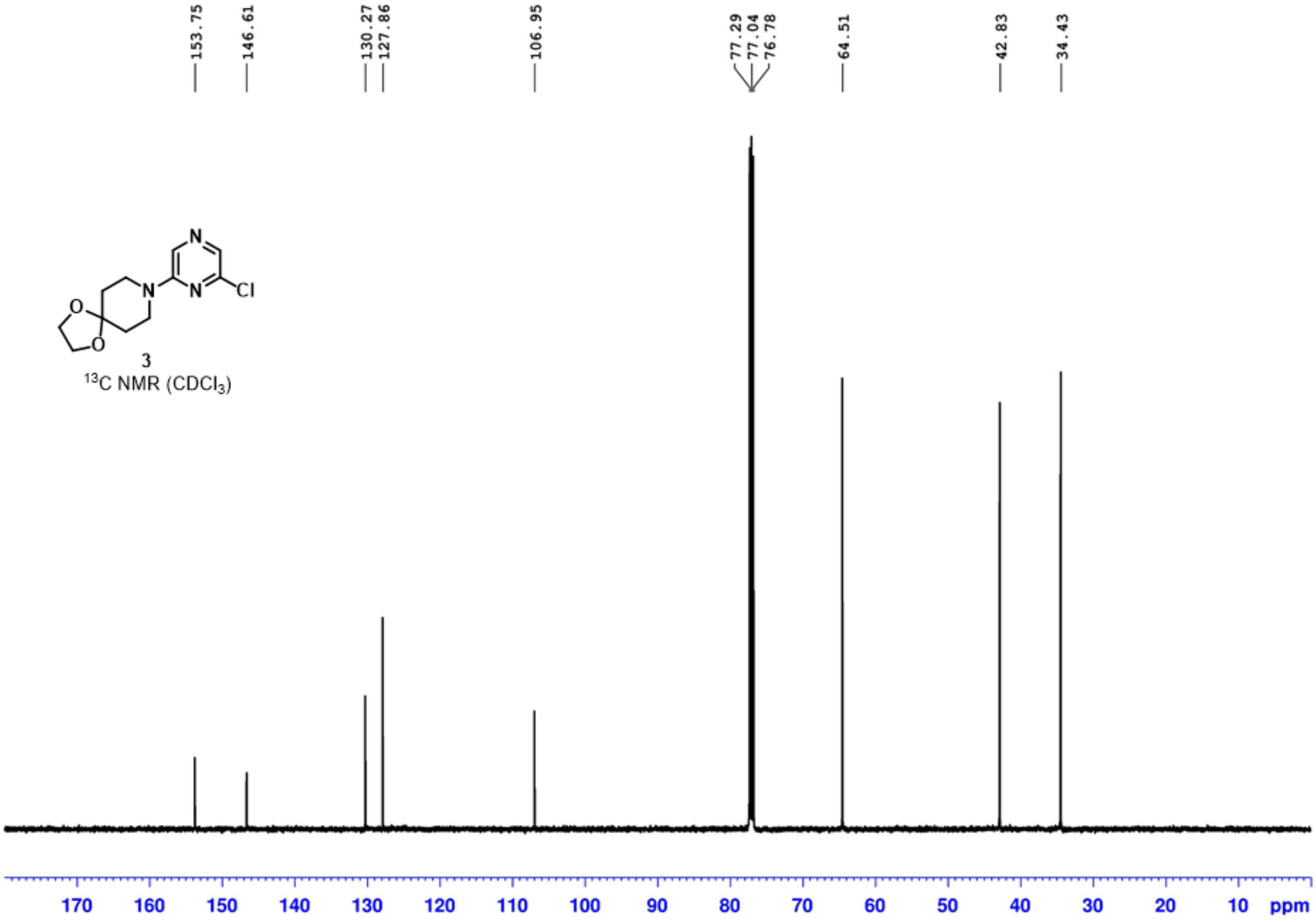

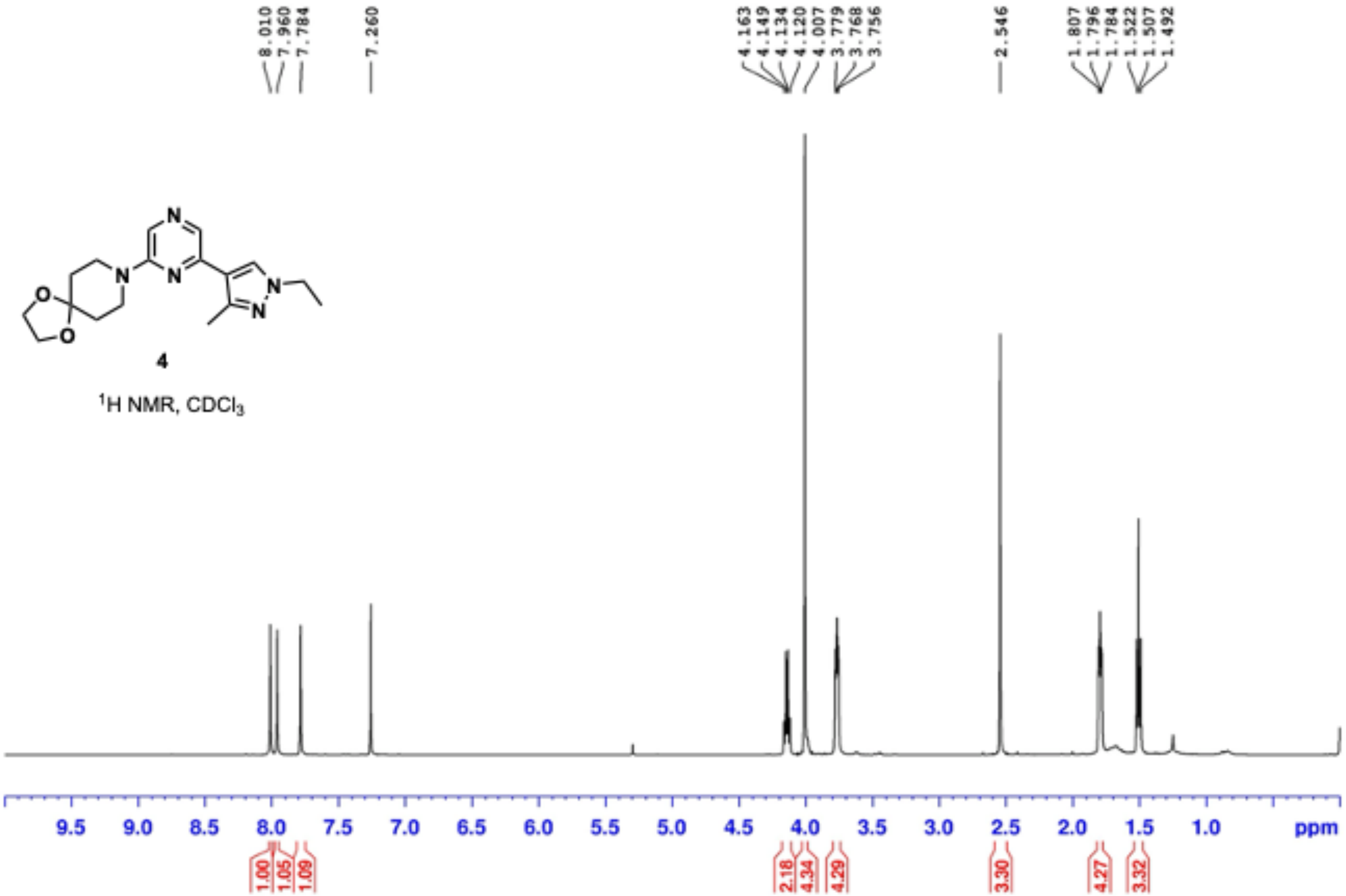
4
^{1}H NMR, CDCl$_3$
8.010
7.960
7.784
7.260
4.163
4.149
4.134
4.120
4.007
3.779
3.768
3.756
2.546
1.807
1.796
1.784
1.522
1.507
1.492
9.5
9.0
8.5
8.0
7.5
7.0
6.5
6.0
5.5
5.0
4.5
4.0
3.5
3.0
2.5
2.0
1.5
1.0
ppm
1.00
1.05
1.09
2.18
4.34
4.29
3.30
4.27
3.32

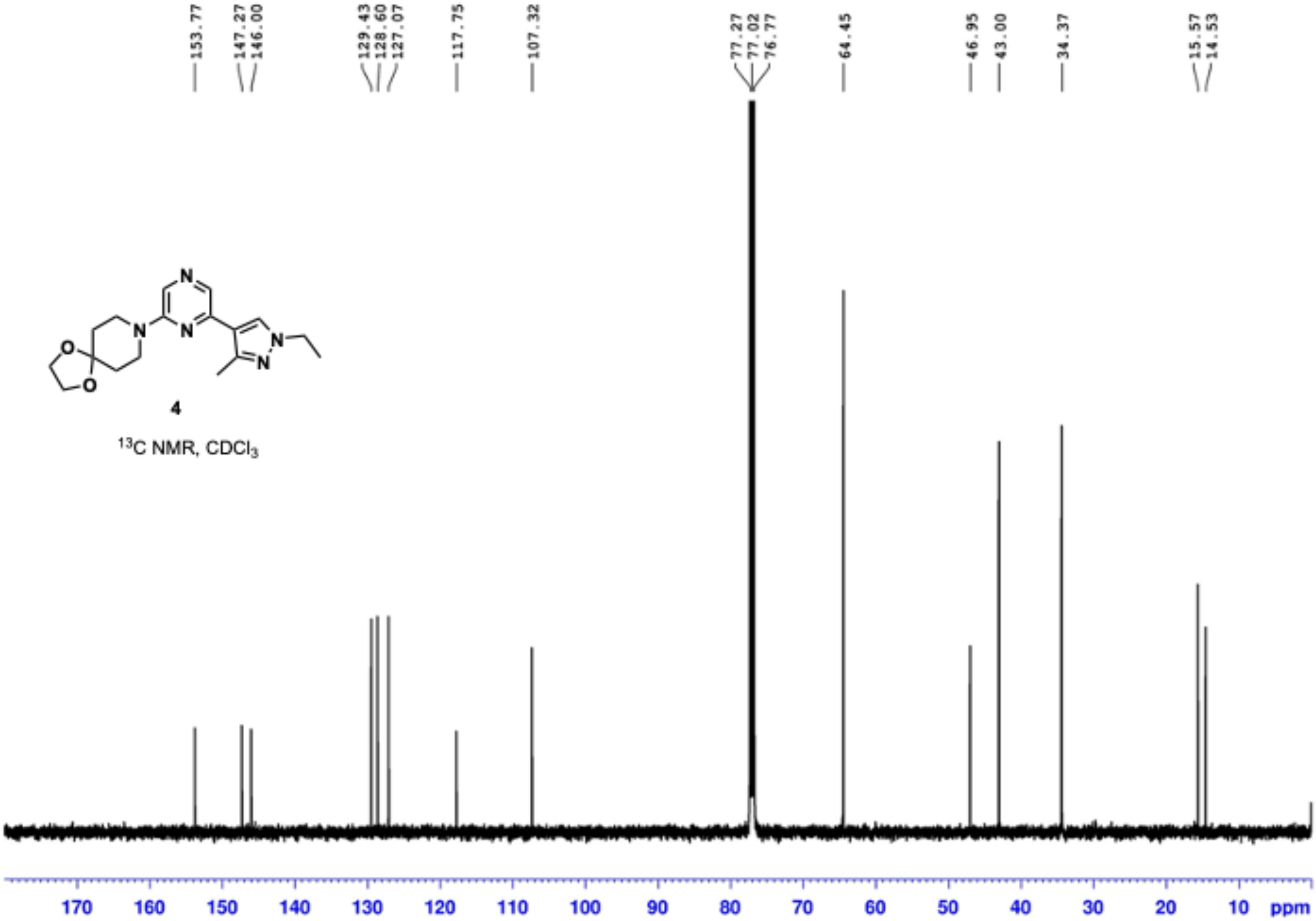
153.77
147.27
146.00
129.43
128.60
127.07
117.75
107.32
77.27
77.02
76.77
64.45
46.95
43.00
34.37
15.57
14.53
4
^{13}C NMR, $CDCl_3$
170 160 150 140 130 120 110 100 90 80 70 60 50 40 30 20 10 ppm

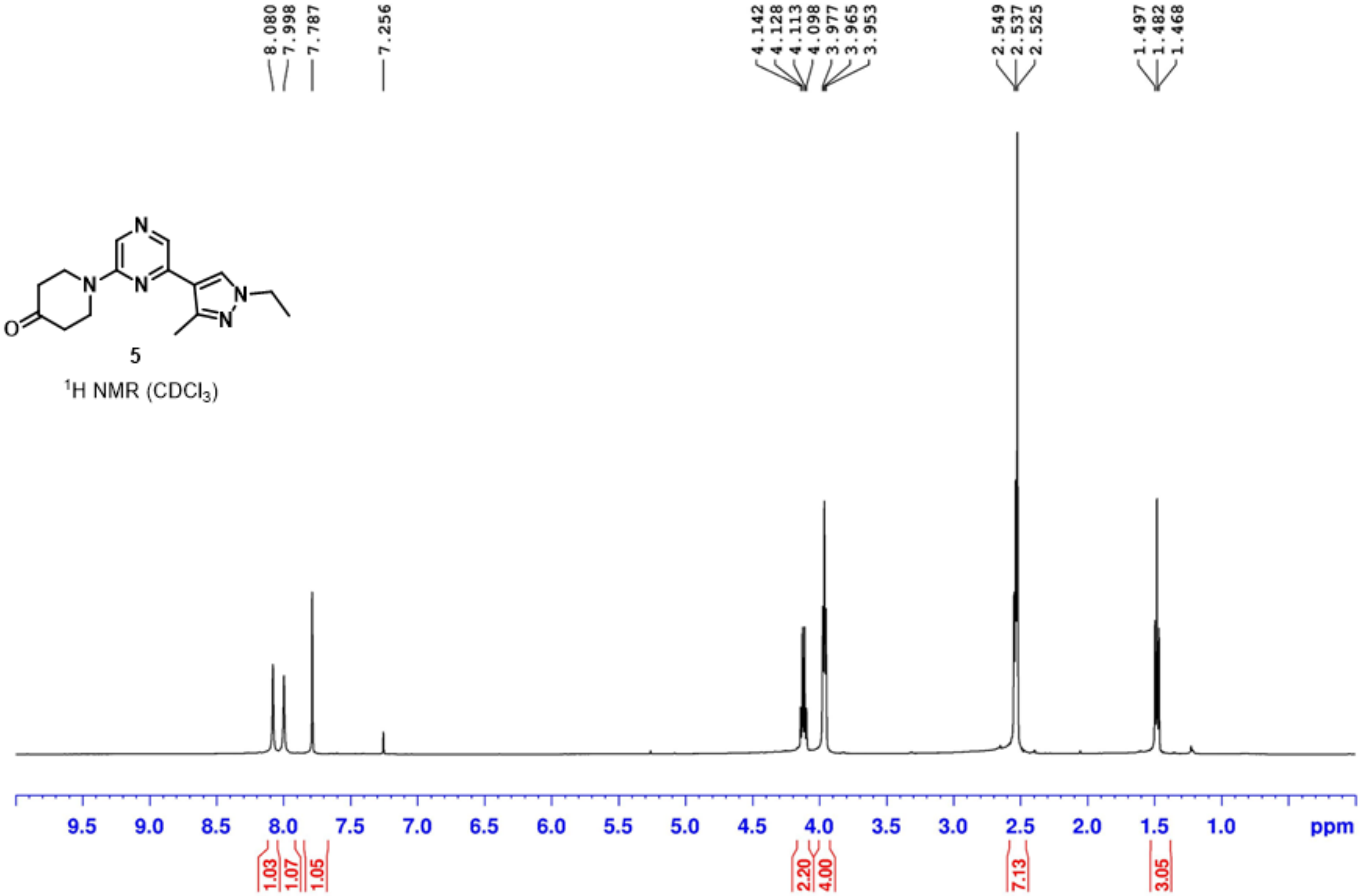

5

^{1}H NMR ($CDCl_3$)

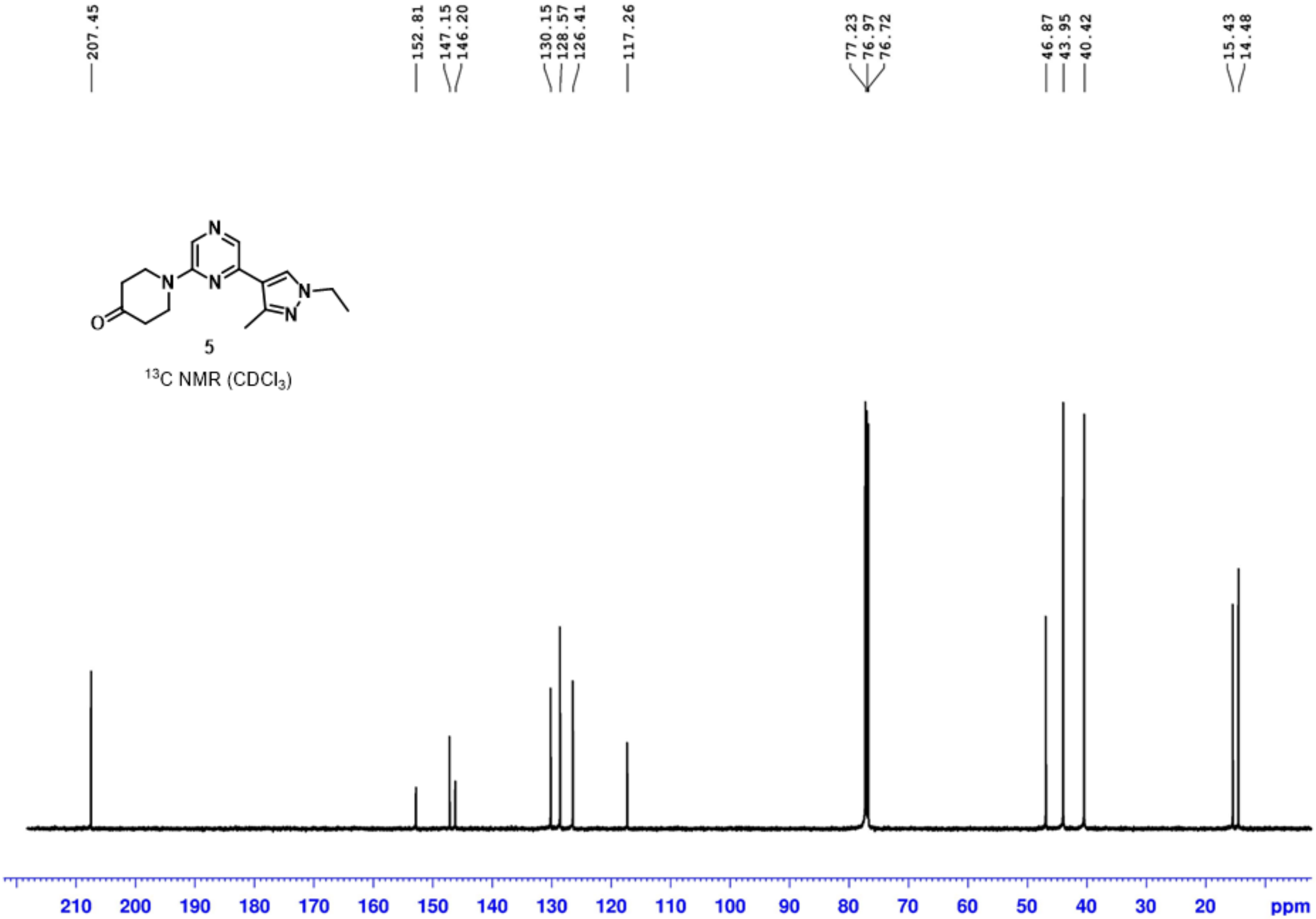

5

^{13}C NMR ($CDCl_3$)

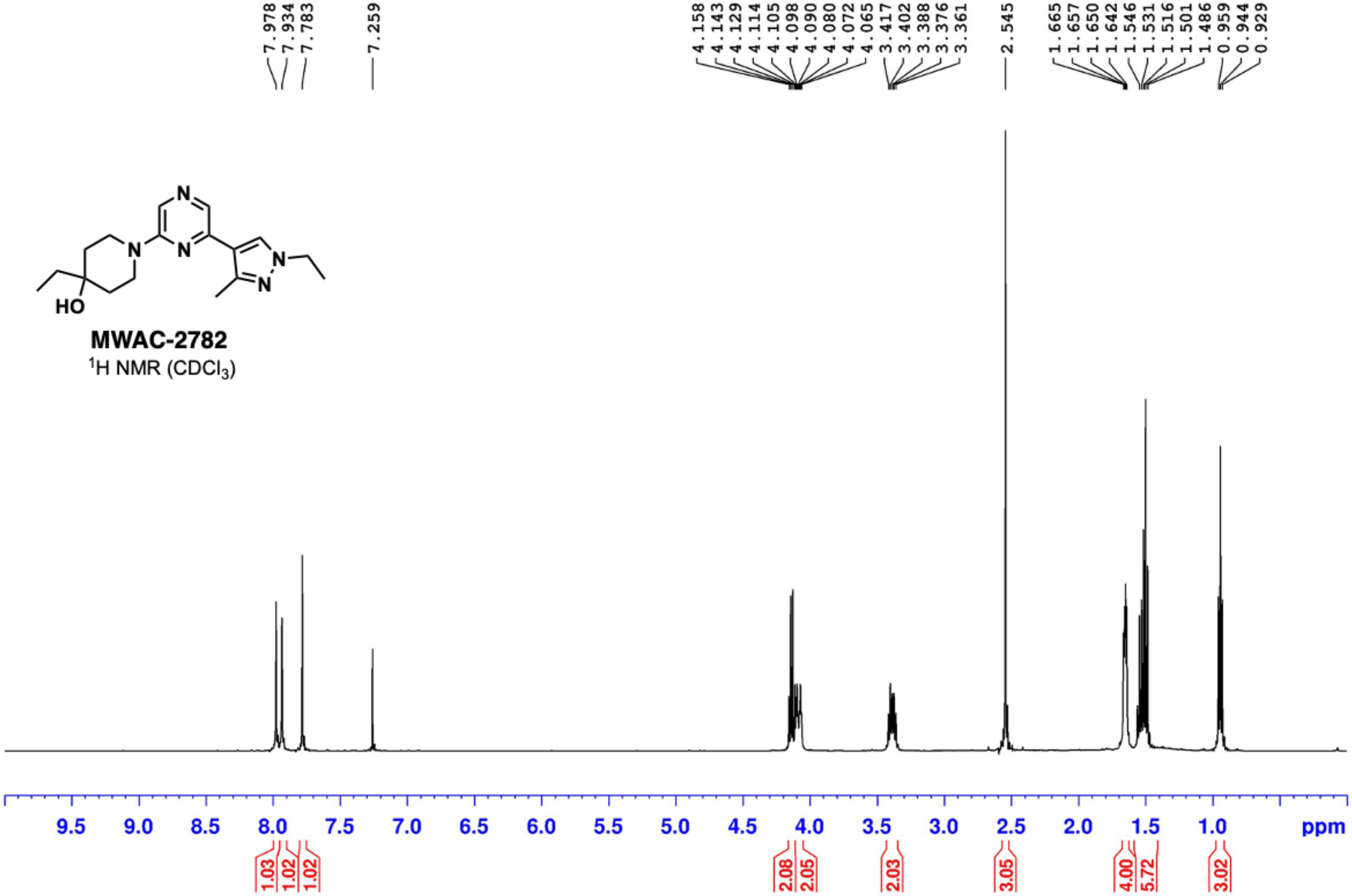
MWAC-2782
1H NMR (CDCl3)
HO
N
7.978
7.934
7.783
7.259
4.158
4.143
4.129
4.114
4.105
4.098
4.090
4.080
4.072
4.065
3.417
3.402
3.388
3.376
3.361
2.545
1.665
1.657
1.650
1.642
1.546
1.531
1.516
1.501
1.486
0.959
0.944
0.929
9.5 9.0 8.5 8.0 7.5 7.0 6.5 6.0 5.5 5.0 4.5 4.0 3.5 3.0 2.5 2.0 1.5 1.0 ppm
1.03
1.02
1.02
2.08
2.05
2.03
3.05
4.00
5.72
3.02

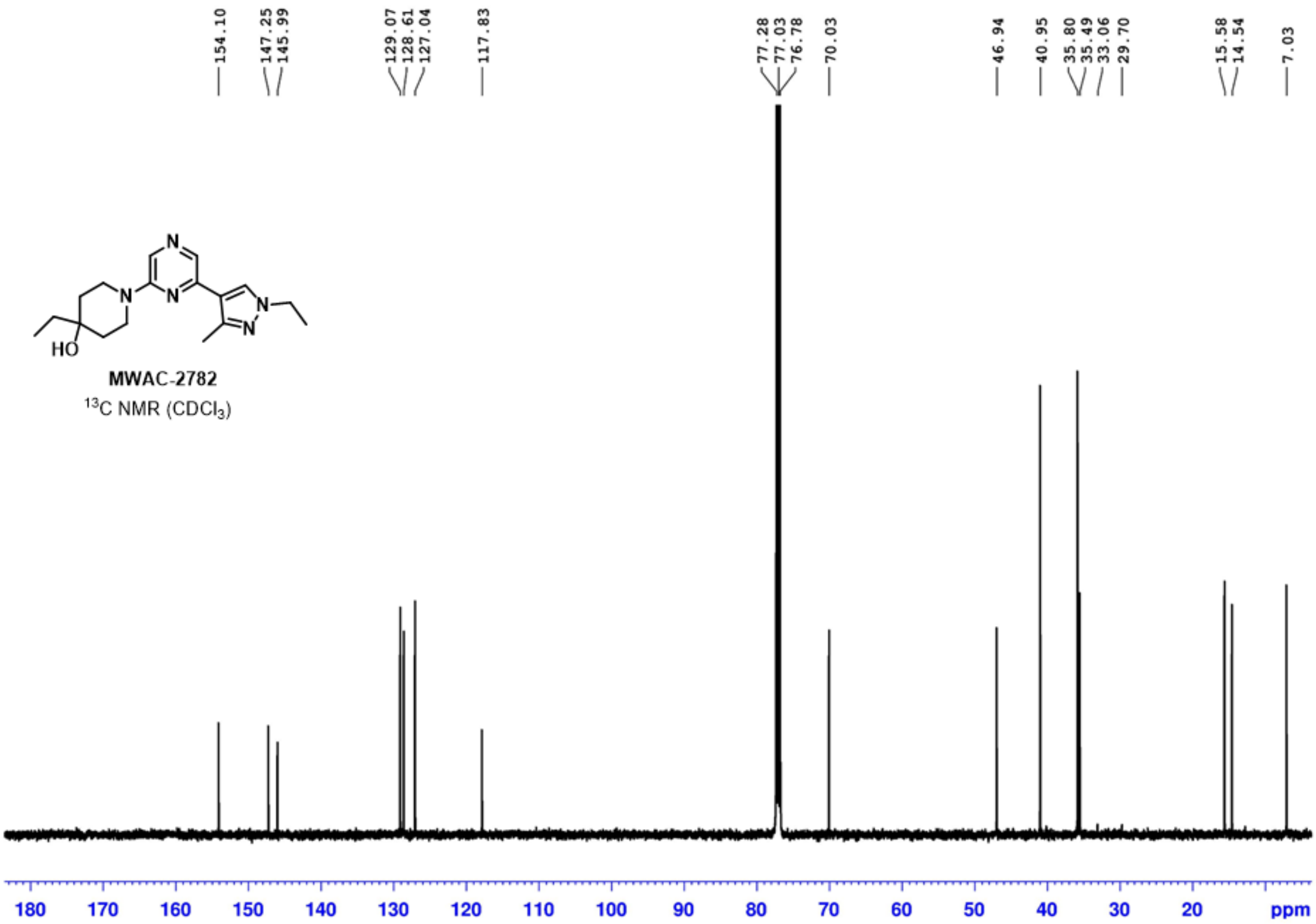
154.10
147.25
145.99
129.07
128.61
127.04
117.83
77.28
77.03
76.78
70.03
46.94
40.95
35.80
35.49
33.06
29.70
15.58
14.54
7.03
N
N
N
N
N
HO
MWAC-2782
^{13}C NMR ($CDCl_3$)
180
170
160
150
140
130
120
110
100
90
80
70
60
50
40
30
20
ppm

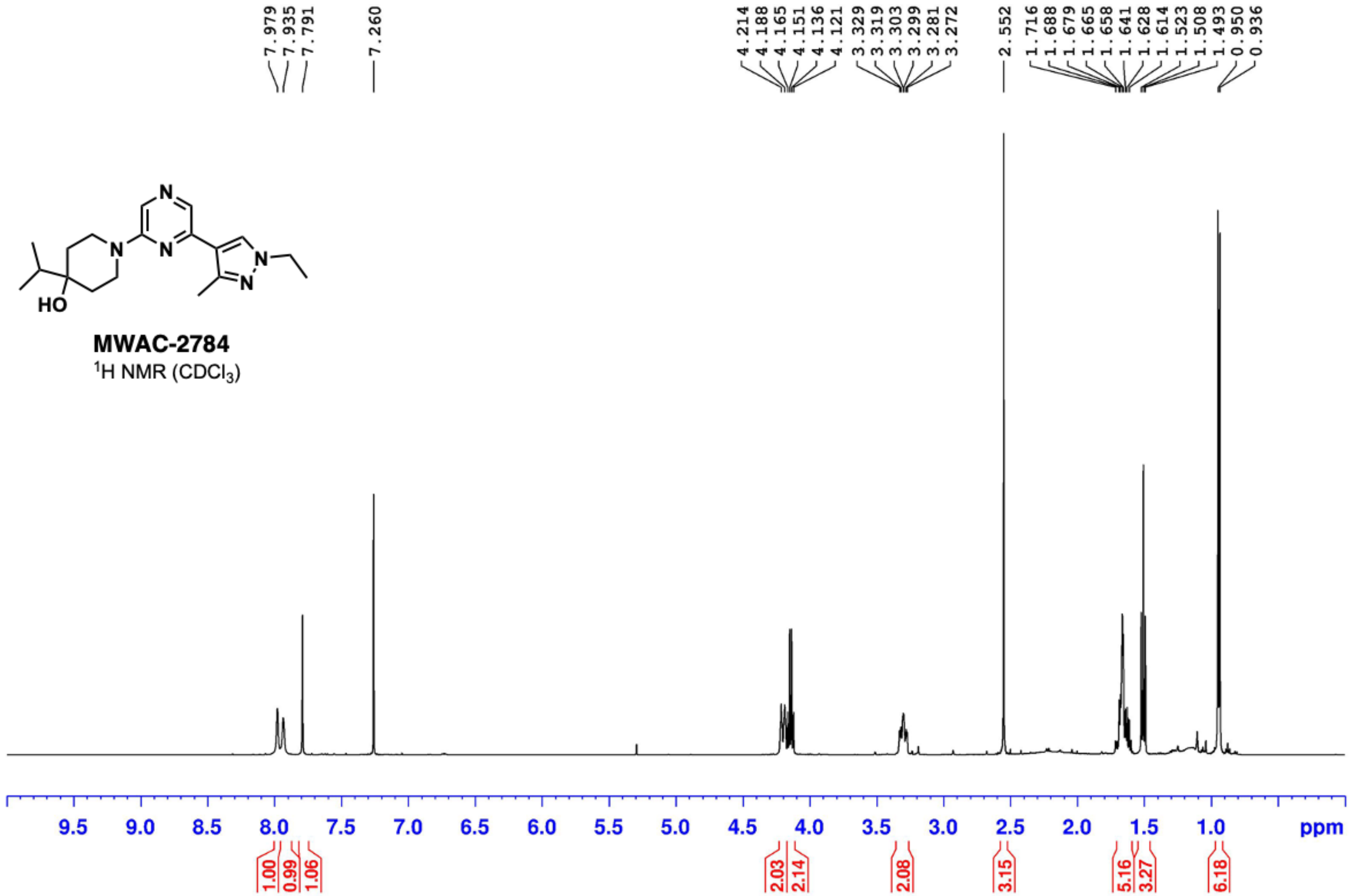

MWAC-2784

^{1}H NMR ($CDCl_3$)

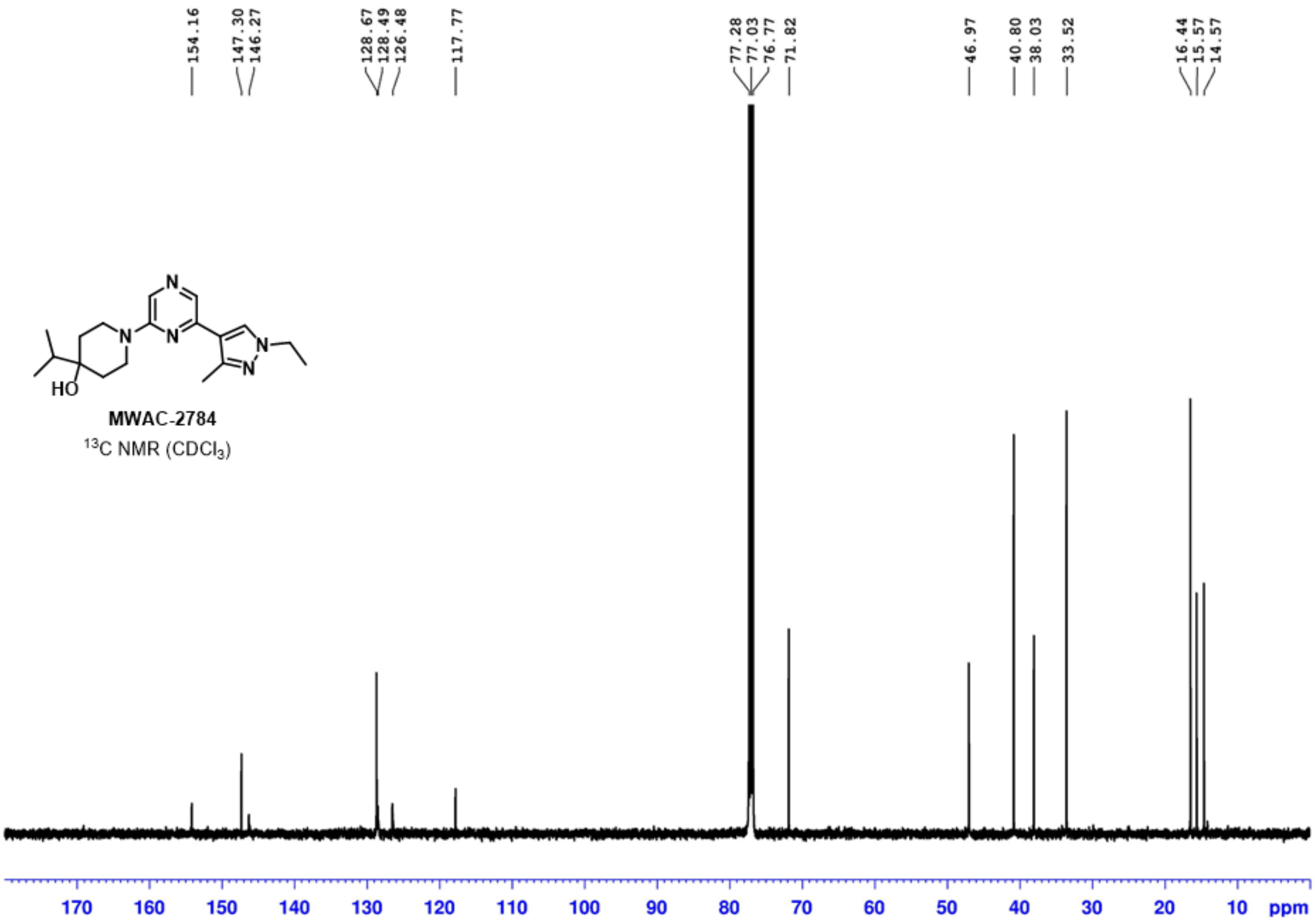

154.16
147.30
146.27
128.67
128.49
126.48
117.77
77.28
77.03
76.77
71.82
46.97
40.80
38.03
33.52
16.44
15.57
14.57
N
N
N
N
N
HO
MWAC-2784
^{13}C NMR ($CDCl_3$)
170 160 150 140 130 120 110 100 90 80 70 60 50 40 30 20 10 ppm

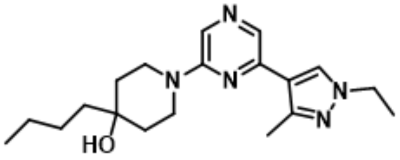

MWAC-2785

^{1}H NMR ($CDCl_3$)

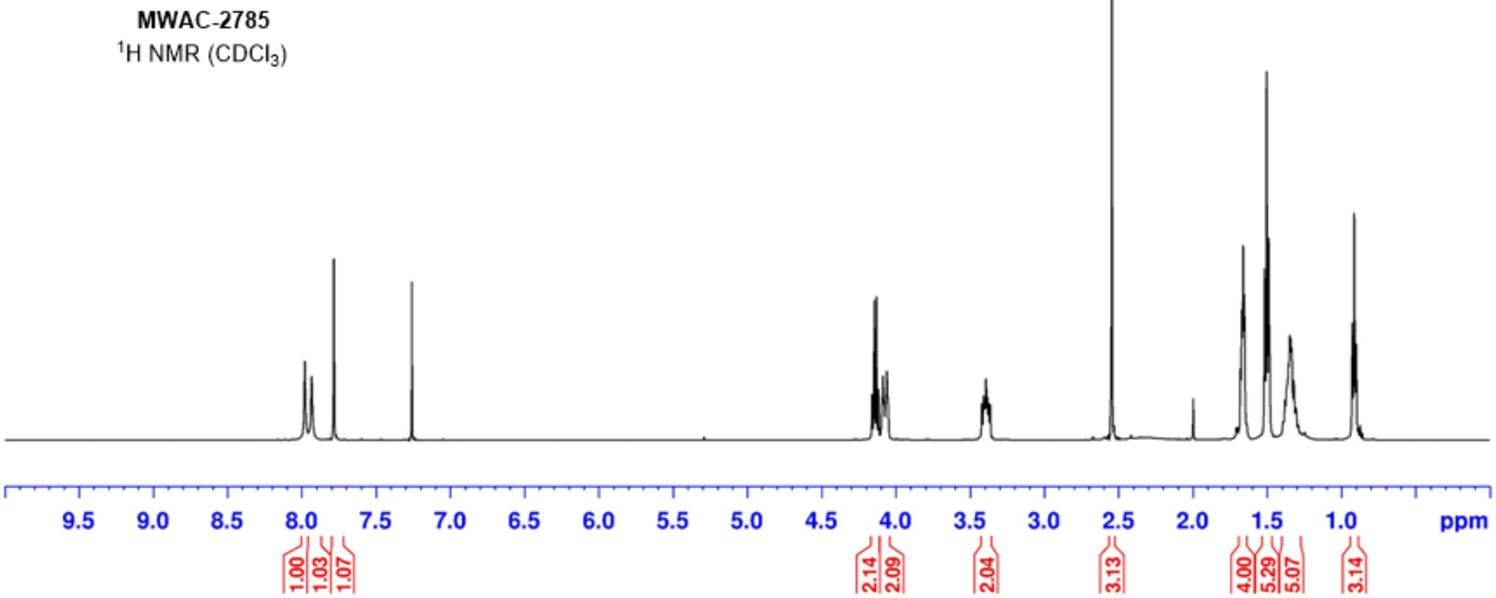

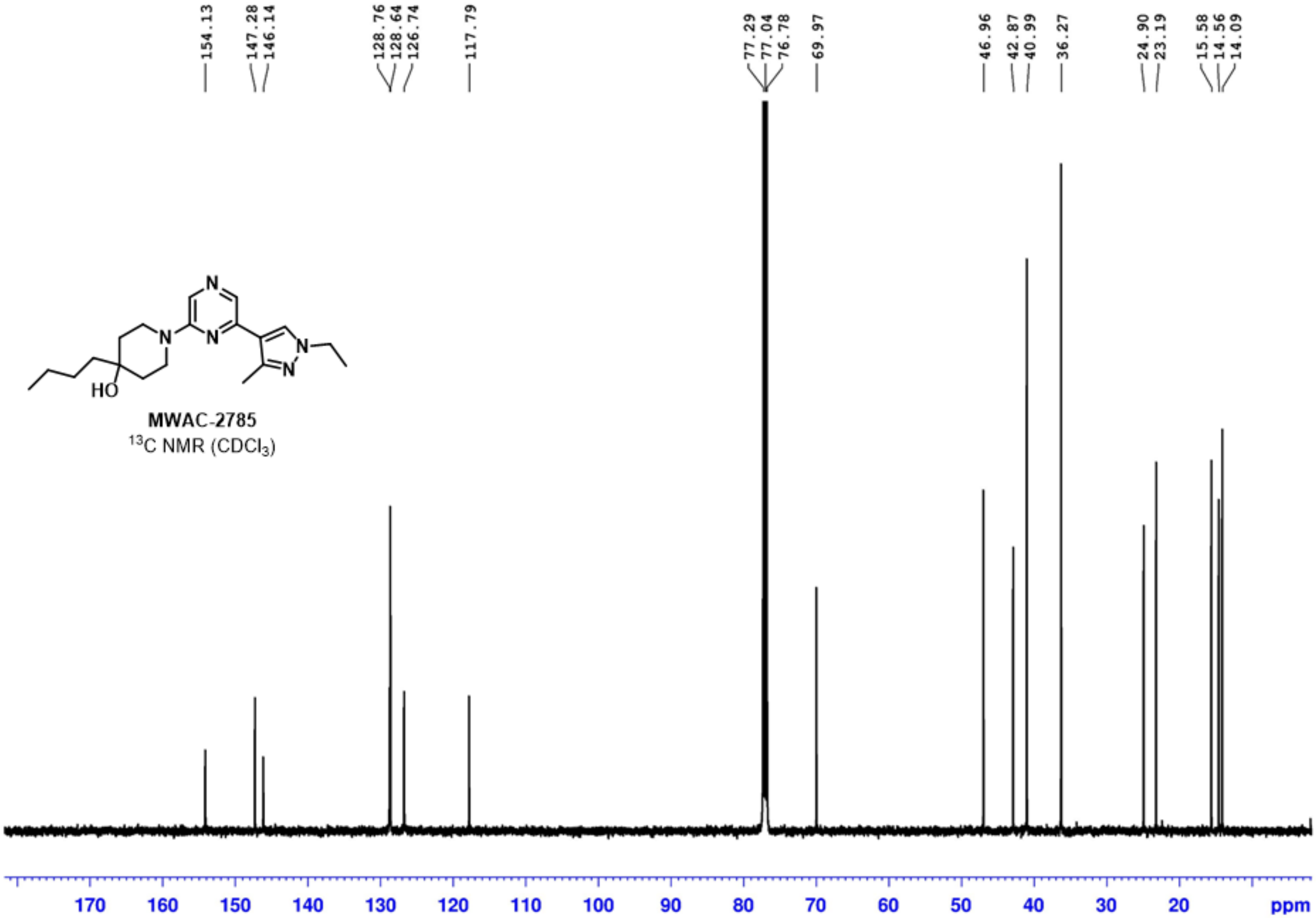
154.13
147.28
146.14
128.76
128.64
126.74
117.79
77.29
77.04
76.78
69.97
46.96
42.87
40.99
36.27
24.90
23.19
15.58
14.56
14.09
MWAC-2785
13C NMR (CDCl3)
170
160
150
140
130
120
110
100
90
80
70
60
50
40
30
20
ppm

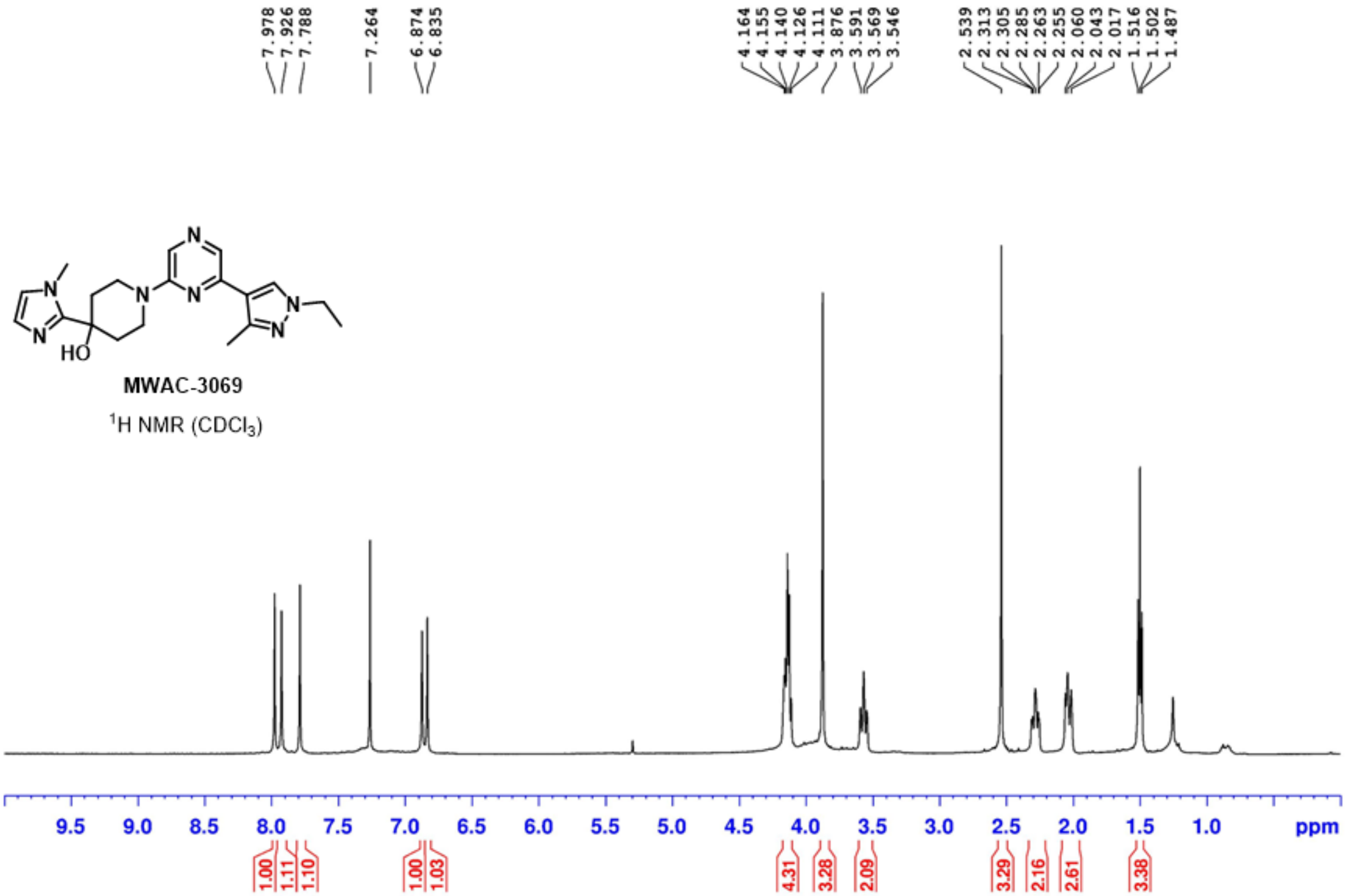
7.978
7.926
7.788
7.264
6.874
6.835
4.164
4.155
4.140
4.126
4.111
3.876
3.591
3.569
3.546
2.539
2.313
2.305
2.285
2.263
2.255
2.060
2.043
2.017
1.516
1.502
1.487
MWAC-3069
1H NMR (CDCl3)
9.5 9.0 8.5 8.0 7.5 7.0 6.5 6.0 5.5 5.0 4.5 4.0 3.5 3.0 2.5 2.0 1.5 1.0 ppm
1.00 1.11 1.10 1.00 1.03 4.31 3.28 2.09 3.29 2.16 2.61 3.38

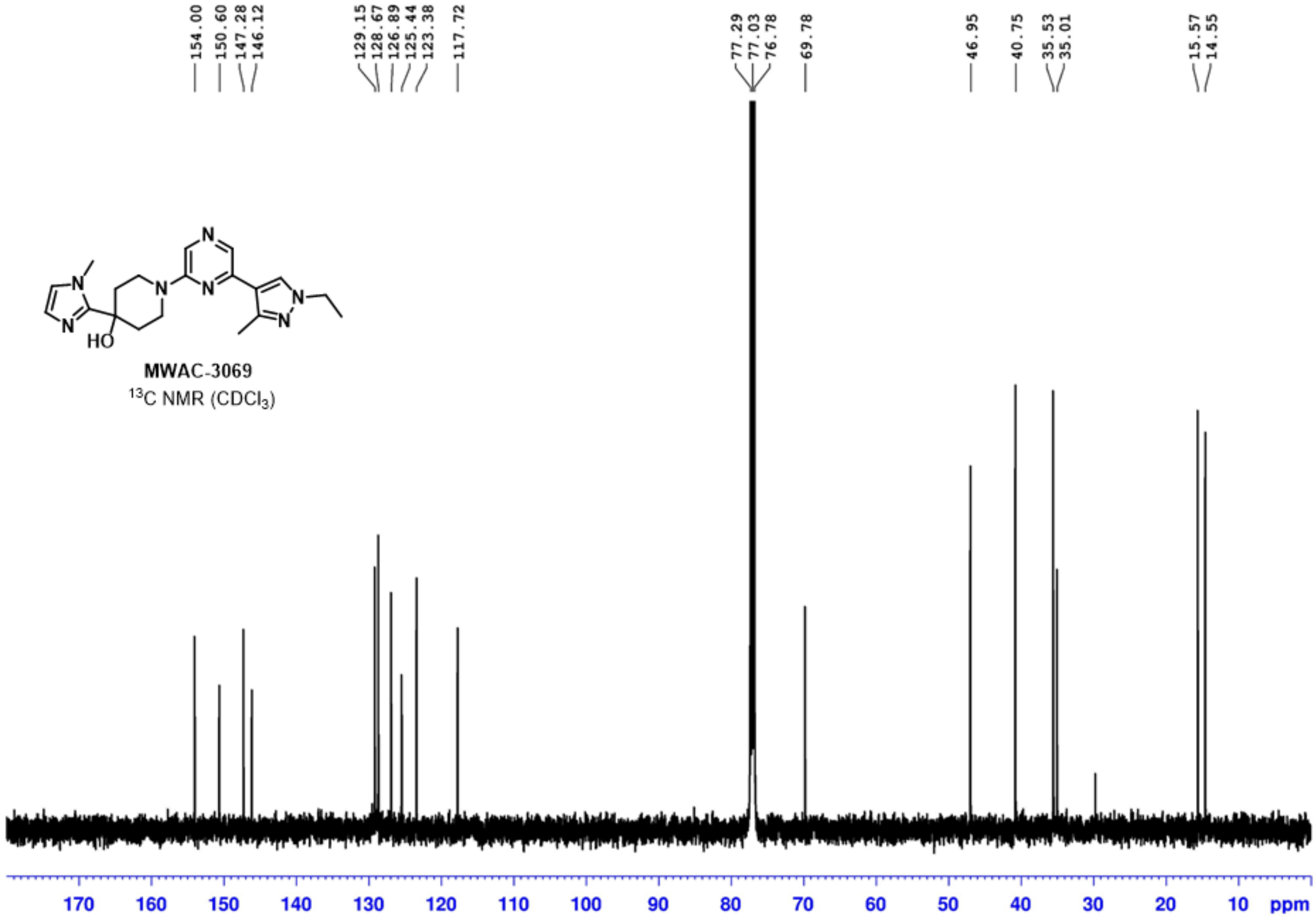

MWAC-3069

^{13}C NMR ($CDCl_3$)

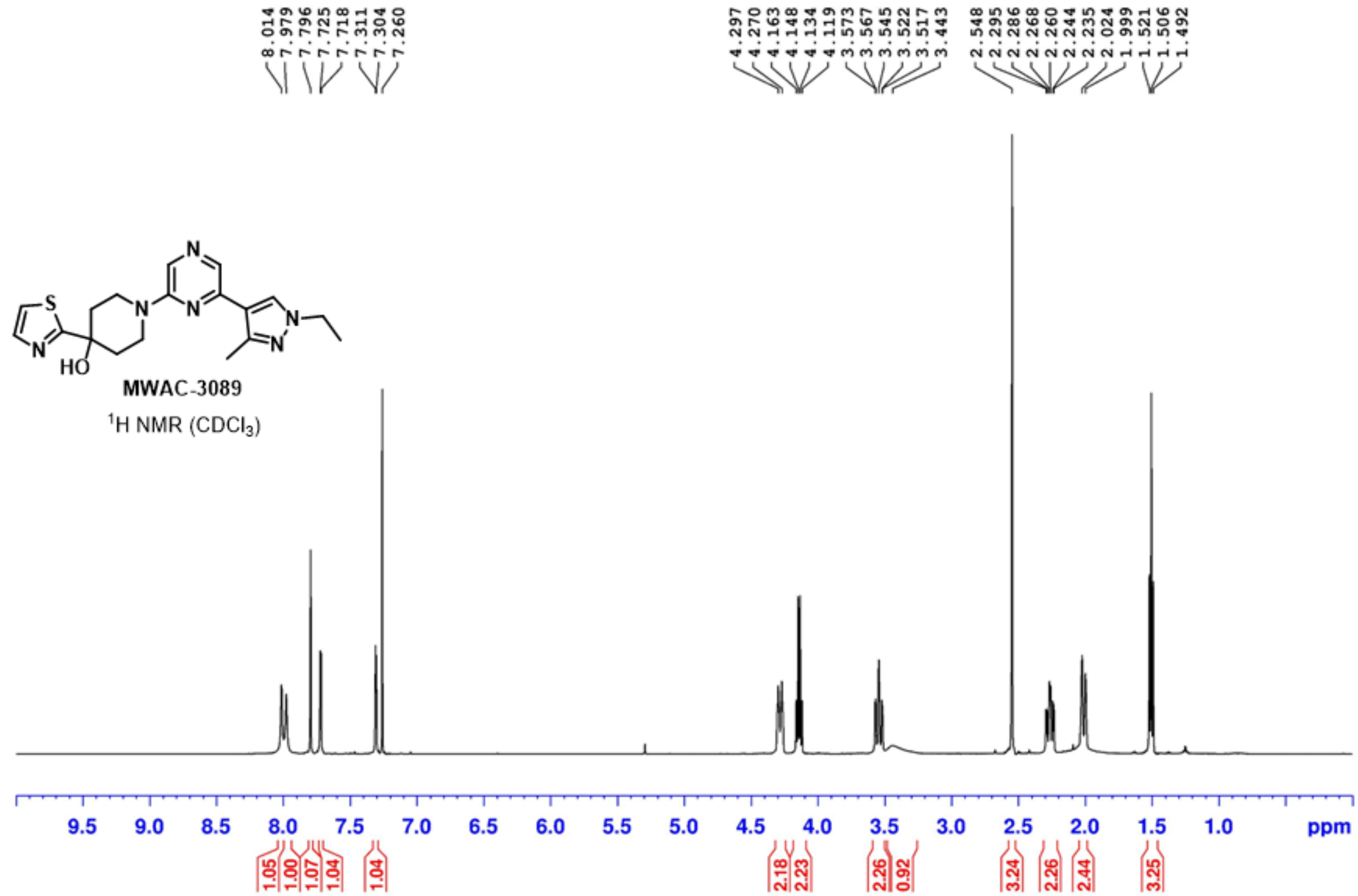
8.014
7.979
7.796
7.725
7.718
7.311
7.304
7.260
4.297
4.270
4.163
4.148
4.134
4.119
3.573
3.567
3.545
3.522
3.517
3.443
2.548
2.295
2.286
2.268
2.260
2.244
2.235
2.024
1.999
1.521
1.506
1.492
MWAC-3089
1H NMR (CDCl3)
9.5
9.0
8.5
8.0
7.5
7.0
6.5
6.0
5.5
5.0
4.5
4.0
3.5
3.0
2.5
2.0
1.5
1.0
ppm
1.05
1.00
1.07
1.04
1.04
2.18
2.23
2.26
0.92
3.24
2.26
2.44
3.25

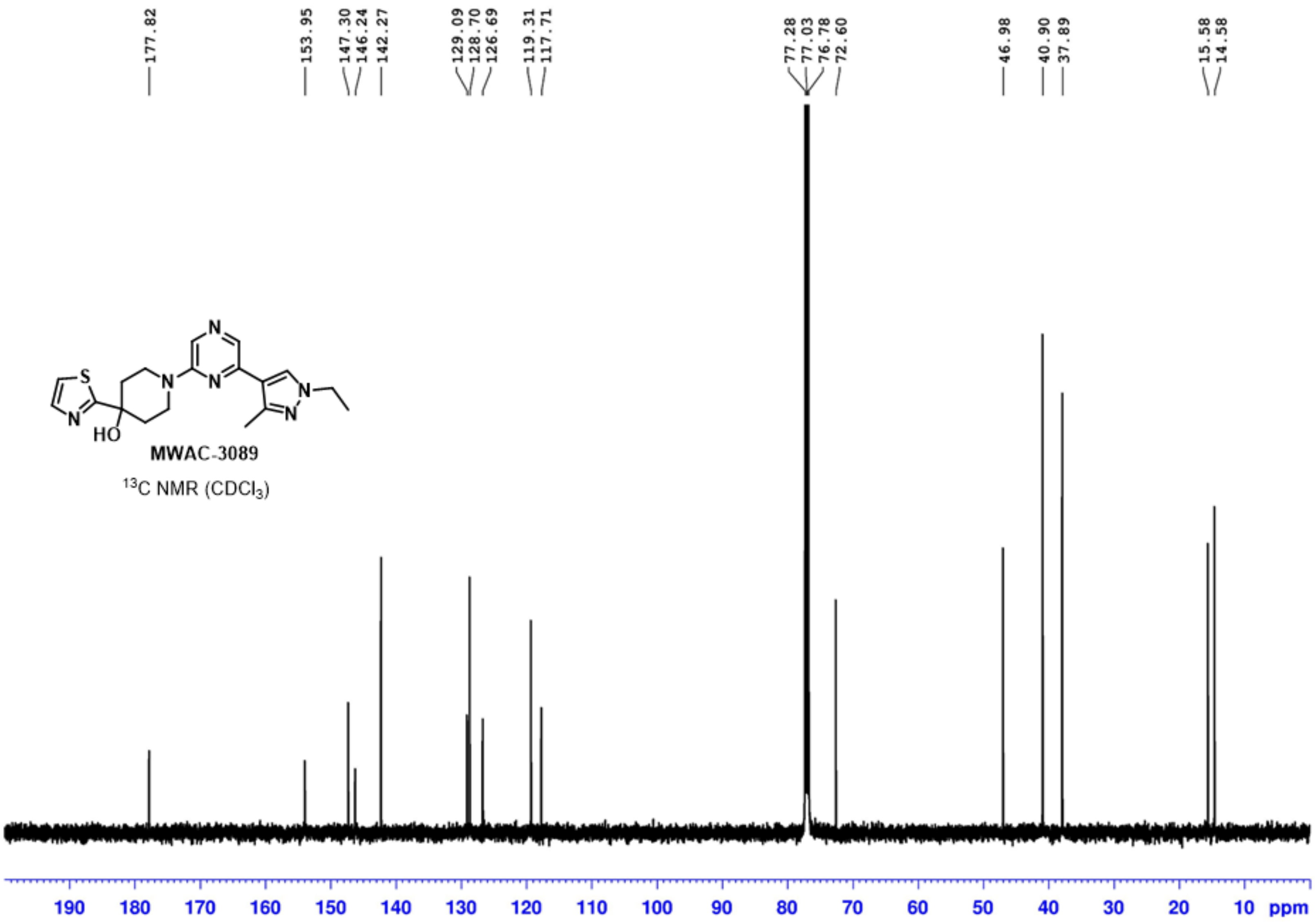
177.82
153.95
147.30
146.24
142.27
129.09
128.70
126.69
119.31
117.71
77.28
77.03
76.78
72.60
46.98
40.90
37.89
15.58
14.58
MWAC-3089
13C NMR (CDCl3)
190
180
170
160
150
140
130
120
110
100
90
80
70
60
50
40
30
20
10
ppm

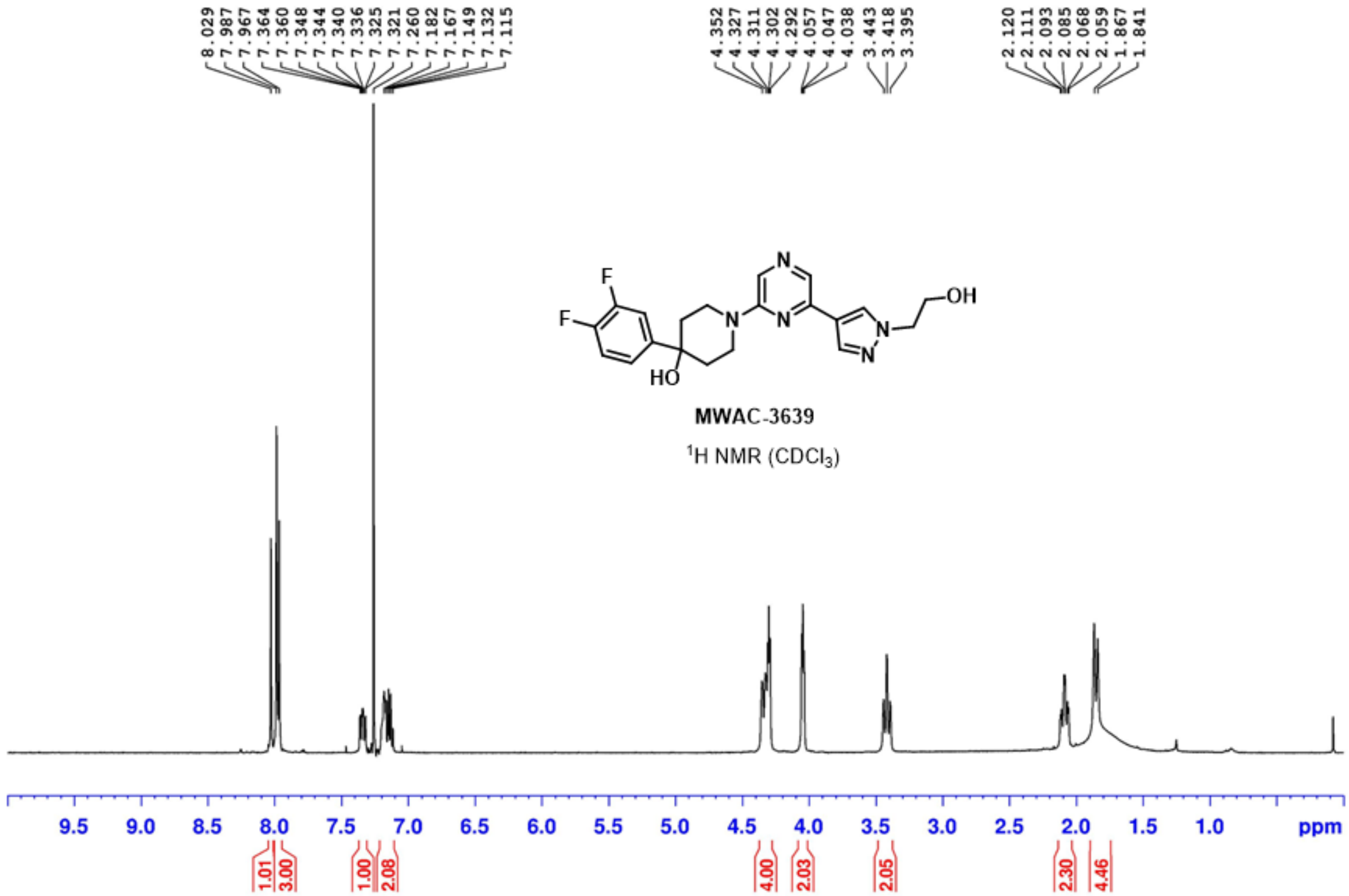
8.029
7.987
7.967
7.364
7.360
7.348
7.344
7.340
7.336
7.325
7.321
7.260
7.182
7.167
7.149
7.132
7.115
4.352
4.327
4.311
4.302
4.292
4.057
4.047
4.038
3.443
3.418
3.395
2.120
2.111
2.093
2.085
2.068
2.059
1.867
1.841
F
F
N
N
N
N
N
OH
HO
MWAC-3639
^{1}H NMR ($CDCl_3$)
9.5
9.0
8.5
8.0
7.5
7.0
6.5
6.0
5.5
5.0
4.5
4.0
3.5
3.0
2.5
2.0
1.5
1.0
ppm
1.01
3.00
1.00
2.08
4.00
2.03
2.05
2.30
4.46

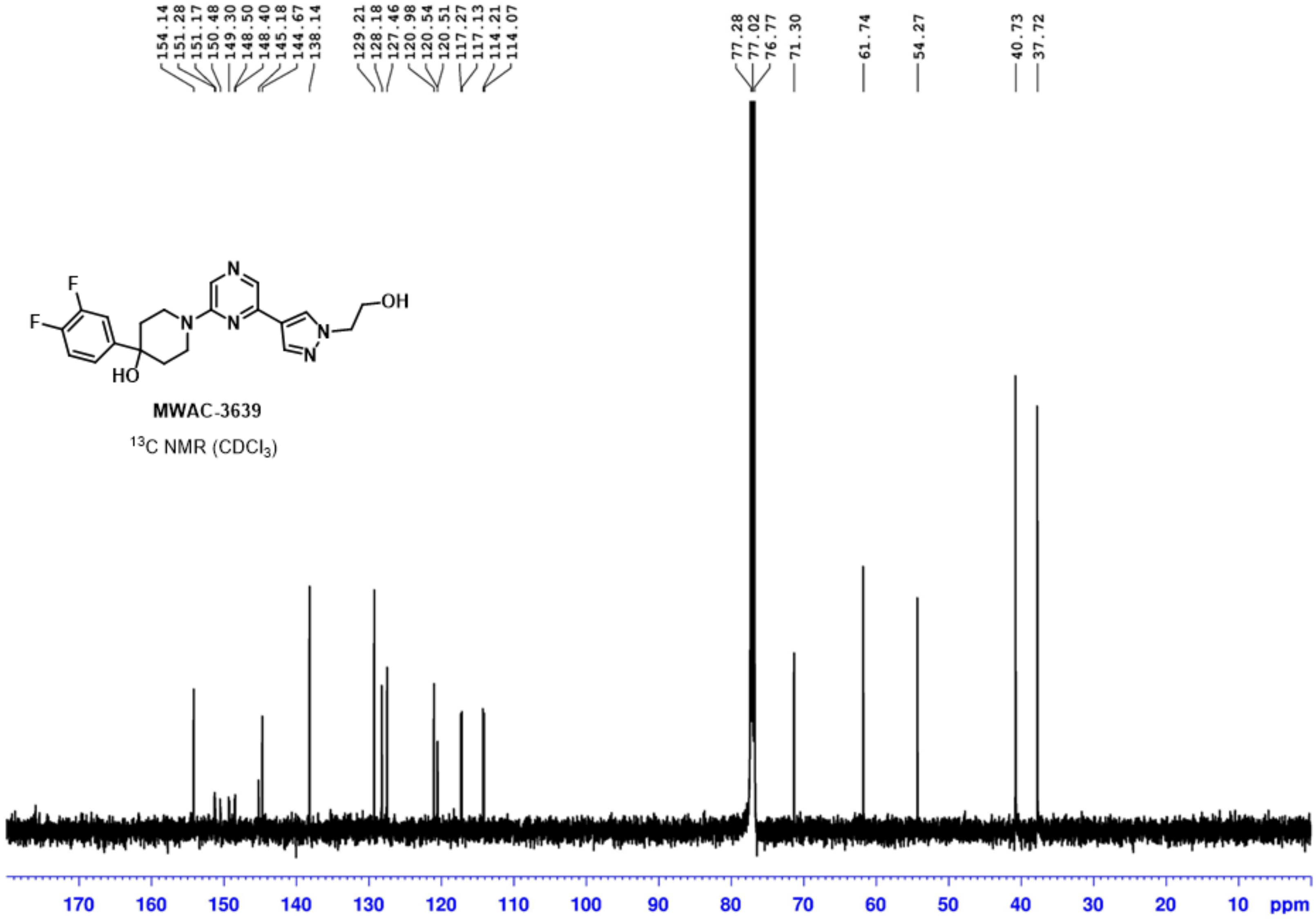
154.14
151.28
151.17
150.48
149.30
148.50
148.40
145.18
144.67
138.14
129.21
128.18
127.46
120.98
120.54
120.51
117.27
117.13
114.21
114.07
77.28
77.02
76.77
71.30
61.74
54.27
40.73
37.72
F
F
HO
N
N
N
N
N
OH
MWAC-3639
13C NMR (CDCl3)
170
160
150
140
130
120
110
100
90
80
70
60
50
40
30
20
10
ppm

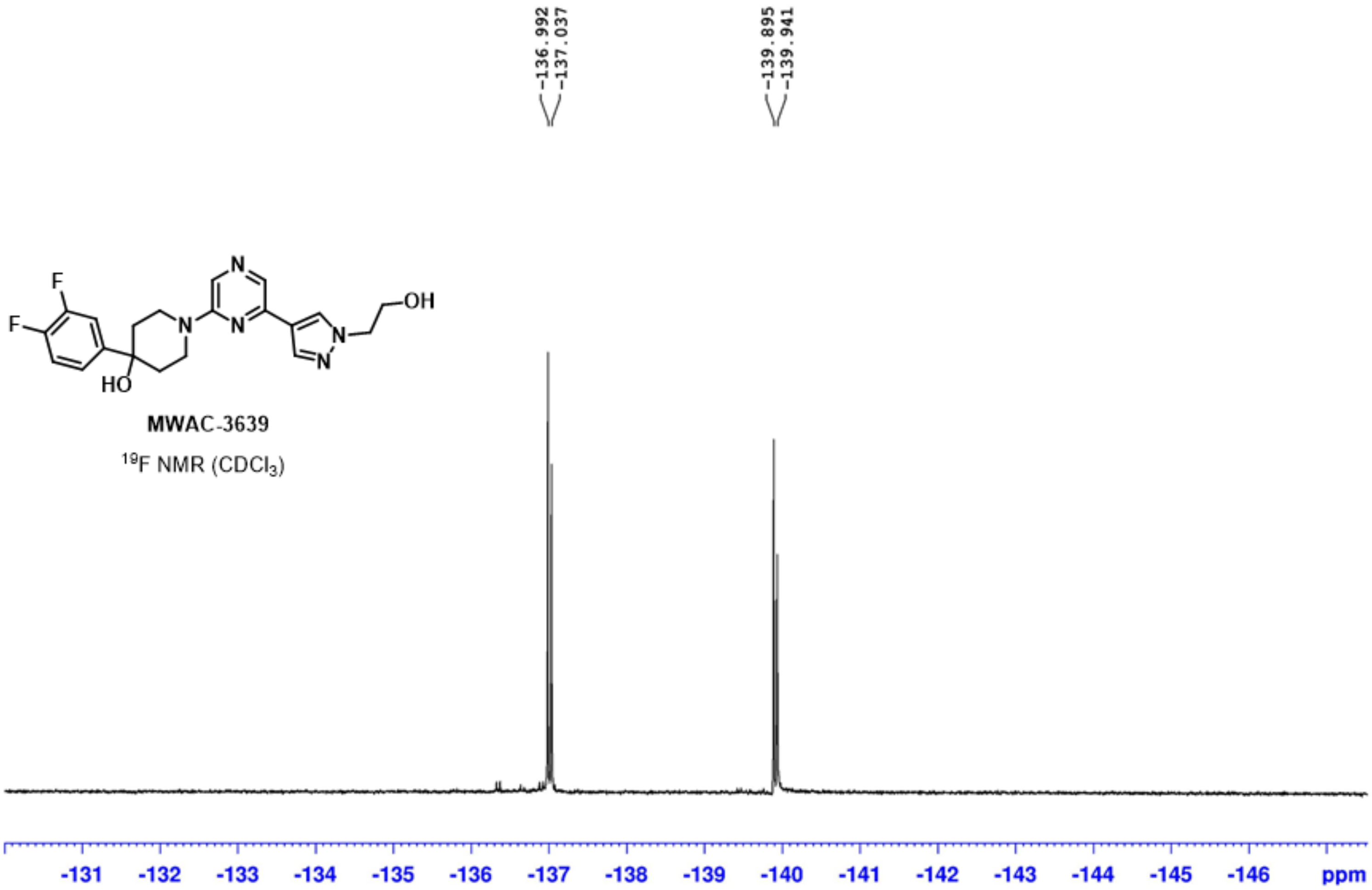
-136.992
-137.037
-139.895
-139.941
F
F
N
N
N
N
N
OH
HO
MWAC-3639
19F NMR (CDCl3)
-131
-132
-133
-134
-135
-136
-137
-138
-139
-140
-141
-142
-143
-144
-145
-146
ppm

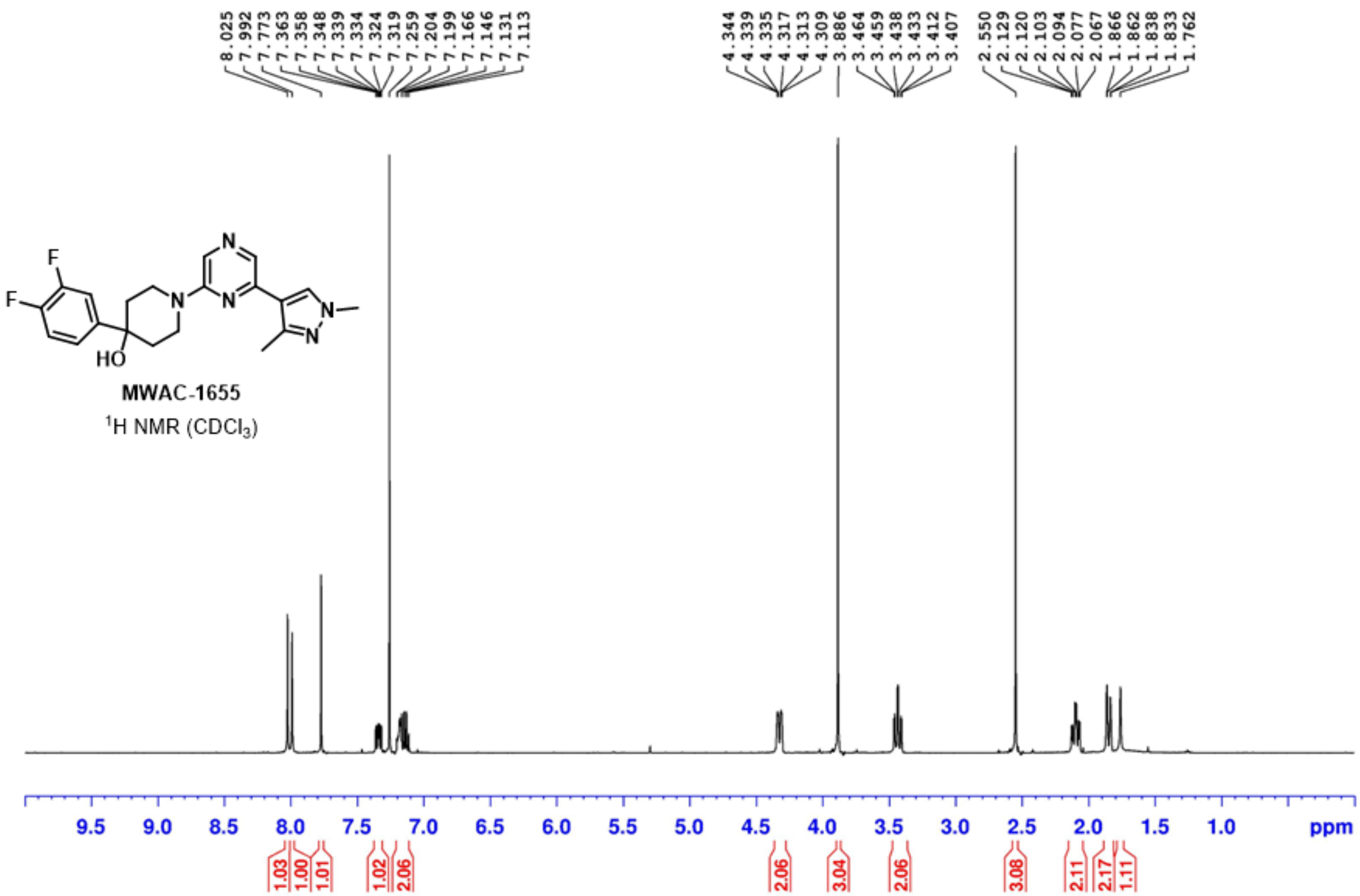

MWAC-1655
1H NMR (CDCl3)
8.025
7.992
7.773
7.363
7.358
7.348
7.339
7.334
7.324
7.319
7.259
7.204
7.199
7.166
7.146
7.131
7.113
4.344
4.339
4.335
4.317
4.313
4.309
3.886
3.464
3.459
3.438
3.433
3.412
3.407
2.550
2.129
2.120
2.103
2.094
2.077
2.067
1.866
1.862
1.838
1.833
1.762
9.5
9.0
8.5
8.0
7.5
7.0
6.5
6.0
5.5
5.0
4.5
4.0
3.5
3.0
2.5
2.0
1.5
1.0
ppm
1.03
1.00
1.01
1.02
2.06
2.06
3.04
2.06
3.08
2.11
2.17
1.11

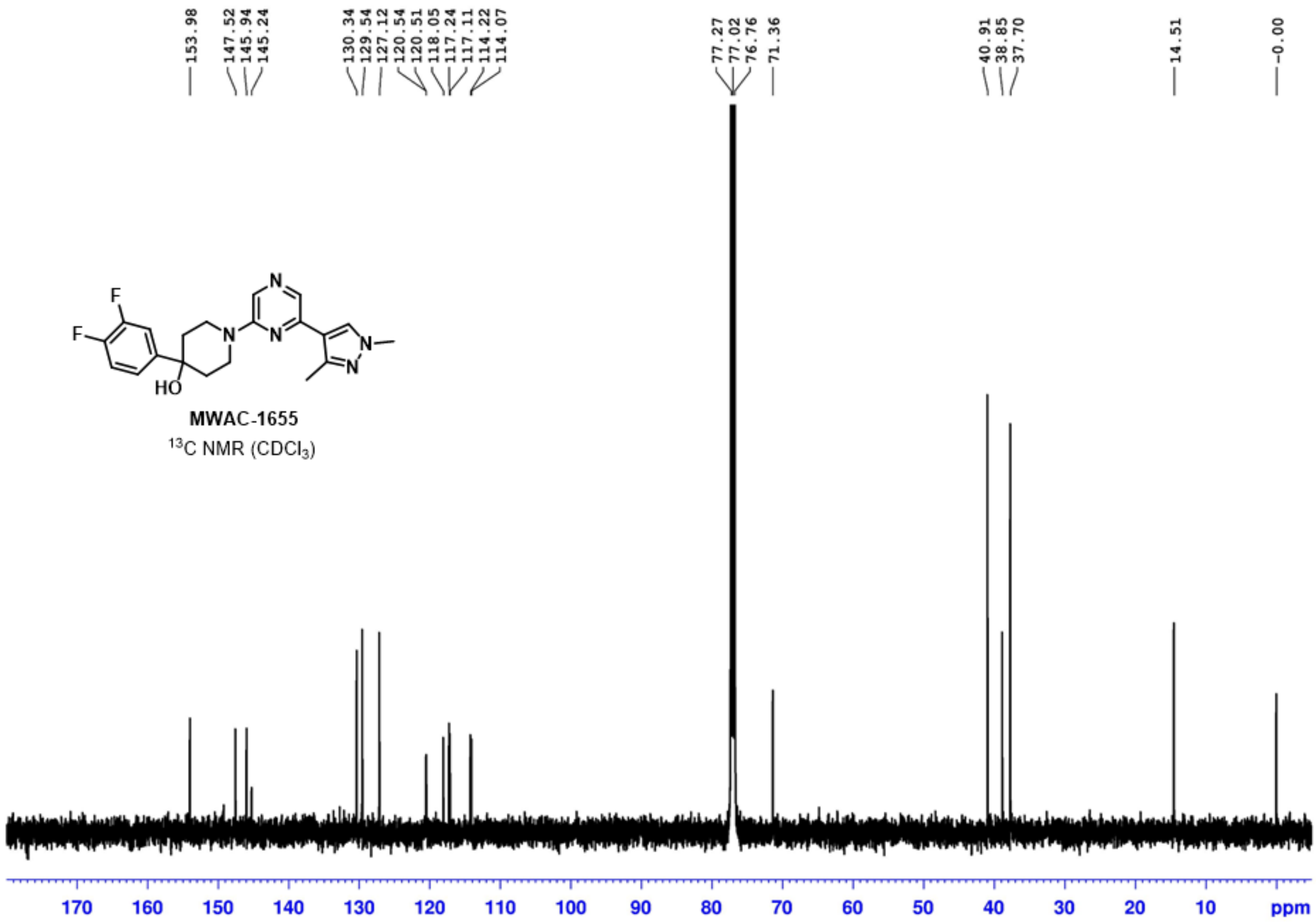
153.98
147.52
145.94
145.24
130.34
129.54
127.12
120.54
120.51
118.05
117.24
117.11
114.22
114.07
77.27
77.02
76.76
71.36
40.91
38.85
37.70
14.51
-0.00
F
F
N
N
N
N
N
HO
MWAC-1655
13C NMR (CDCl3)
170
160
150
140
130
120
110
100
90
80
70
60
50
40
30
20
10
ppm

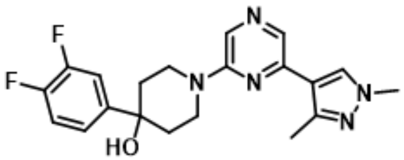

MWAC-1655

^{19}F NMR ($CDCl_3$)

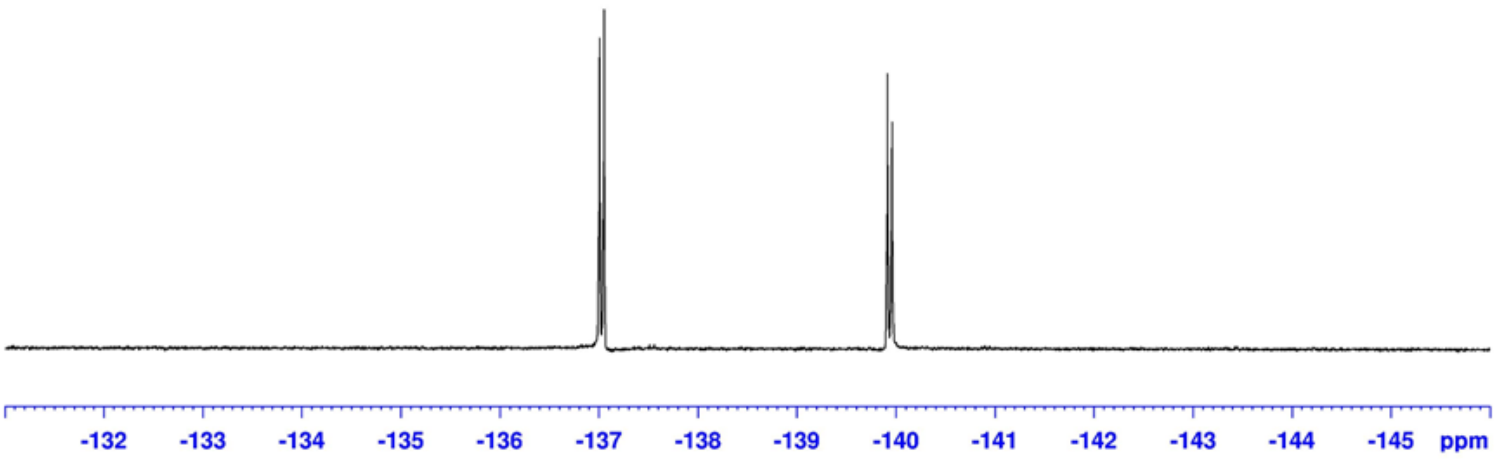

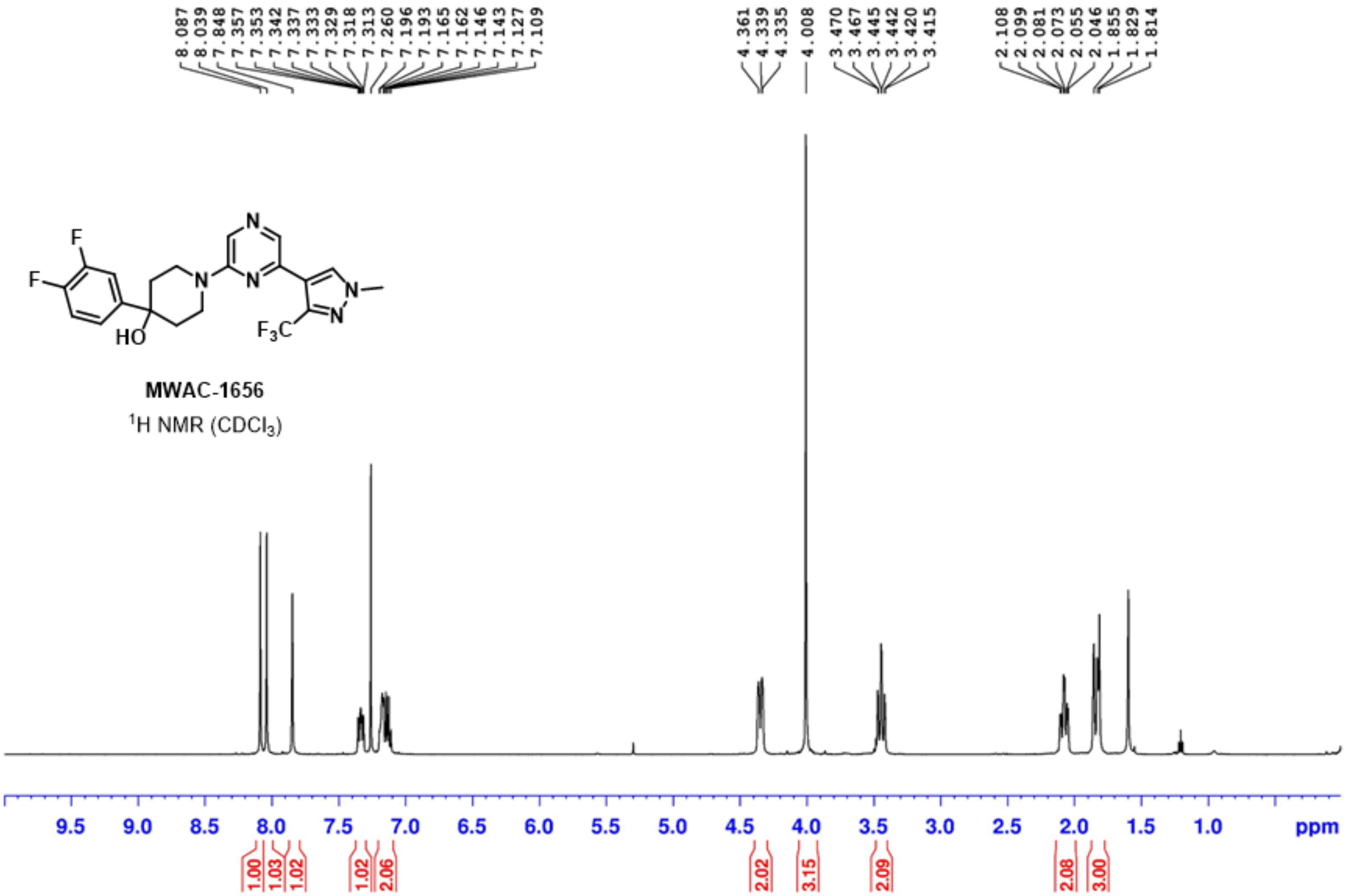
MWAC-1656
^{1}H NMR ($CDCl_3$)
8.087 8.039 7.848 7.357 7.353 7.342 7.337 7.333 7.329 7.318 7.313 7.260 7.196 7.193 7.165 7.162 7.146 7.143 7.127 7.109
4.361 4.339 4.335 4.008 3.470 3.467 3.445 3.442 3.420 3.415
2.108 2.099 2.081 2.073 2.055 2.046 1.855 1.829 1.814
9.5 9.0 8.5 8.0 7.5 7.0 6.5 6.0 5.5 5.0 4.5 4.0 3.5 3.0 2.5 2.0 1.5 1.0 ppm
1.00 1.03 1.02 1.02 2.06 2.02 3.15 2.09 2.08 3.00

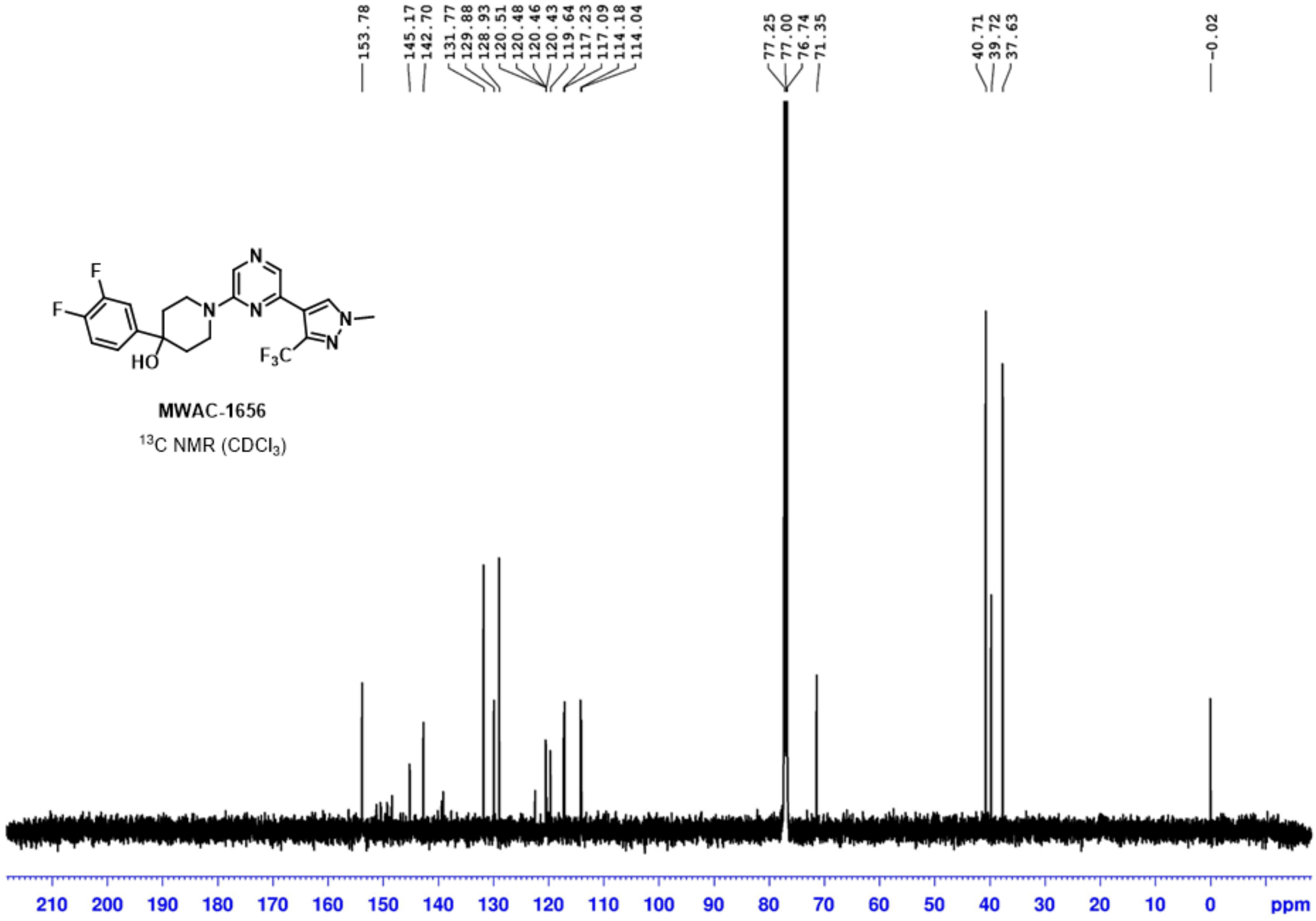

MWAC-1656

^{13}C NMR ($CDCl_3$)

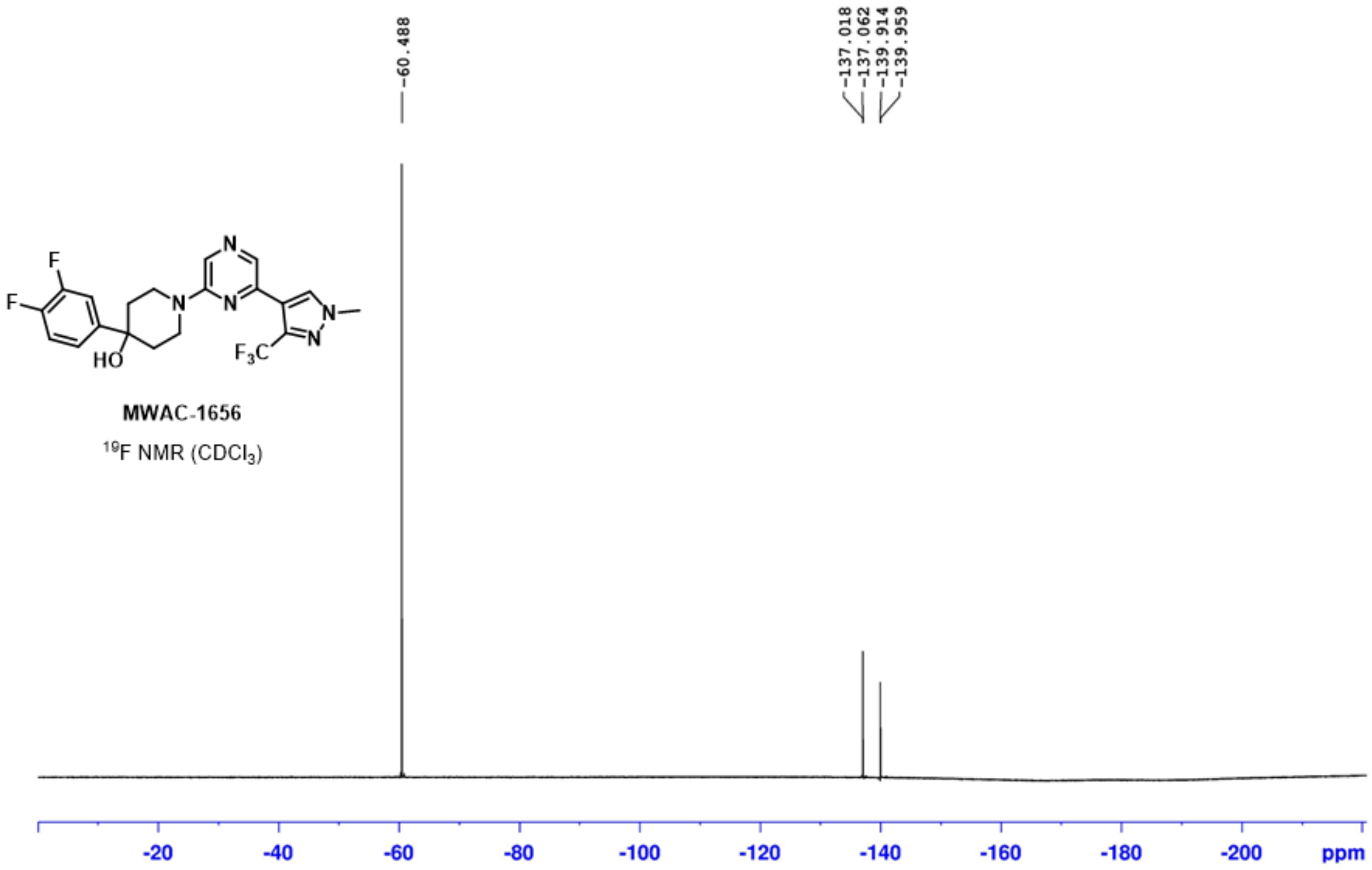

MWAC-1656

^{19}F NMR ($CDCl_3$)

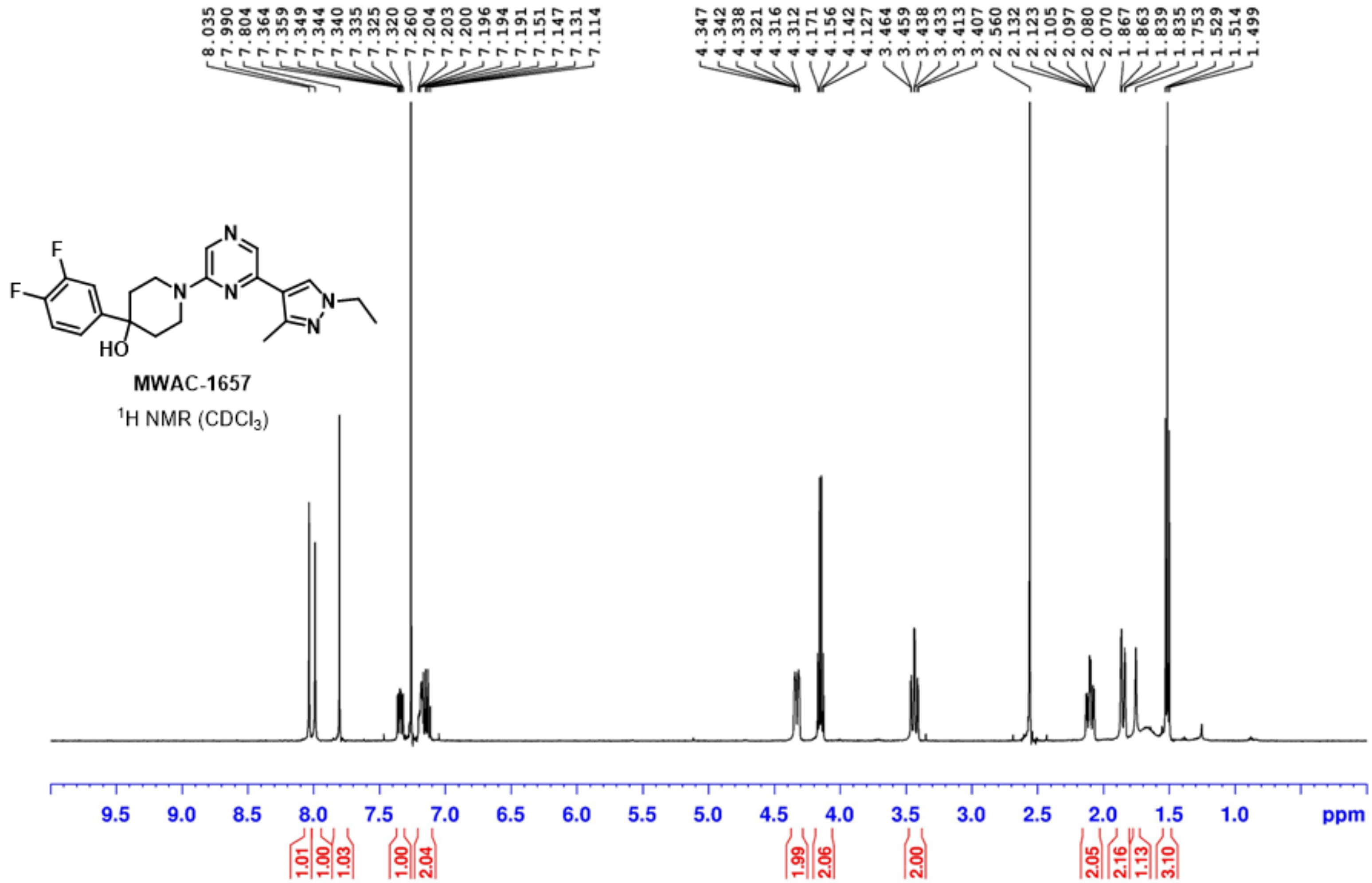

8.035 7.990 7.804 7.364 7.359 7.349 7.344 7.340 7.335 7.325 7.320 7.260 7.204 7.203 7.200 7.196 7.194 7.191 7.151 7.147 7.131 7.114
4.347 4.342 4.338 4.321 4.316 4.312 4.171 4.156 4.142 4.127 3.464 3.459 3.438 3.433 3.413 3.407 2.560 2.132 2.123 2.105 2.097 2.080 2.070 1.867 1.863 1.839 1.835 1.753 1.529 1.514 1.499
F
F
N
N
N
N
N
HO
MWAC-1657
^{1}H NMR ($CDCl_3$)
9.5 9.0 8.5 8.0 7.5 7.0 6.5 6.0 5.5 5.0 4.5 4.0 3.5 3.0 2.5 2.0 1.5 1.0 ppm
1.01 1.00 1.03 1.00 2.04 1.99 2.06 2.00 2.05 2.16 1.13 3.10

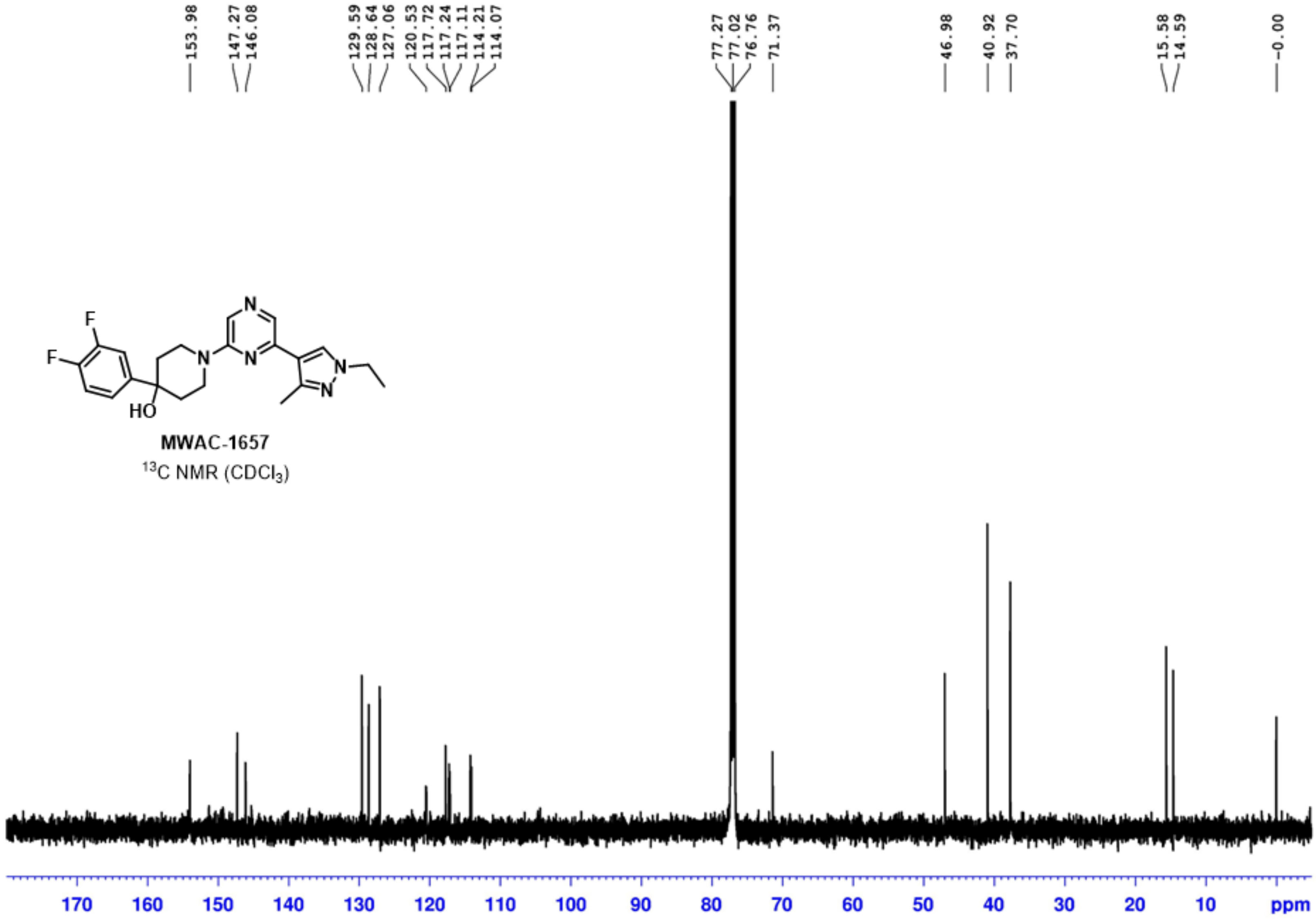
153.98
147.27
146.08
129.59
128.64
127.06
120.53
117.72
117.24
117.11
114.21
114.07
77.27
77.02
76.76
71.37
46.98
40.92
37.70
15.58
14.59
-0.00
F
F
N
N
N
N
N
HO
MWAC-1657
^{13}C NMR ($CDCl_3$)
170
160
150
140
130
120
110
100
90
80
70
60
50
40
30
20
10
ppm

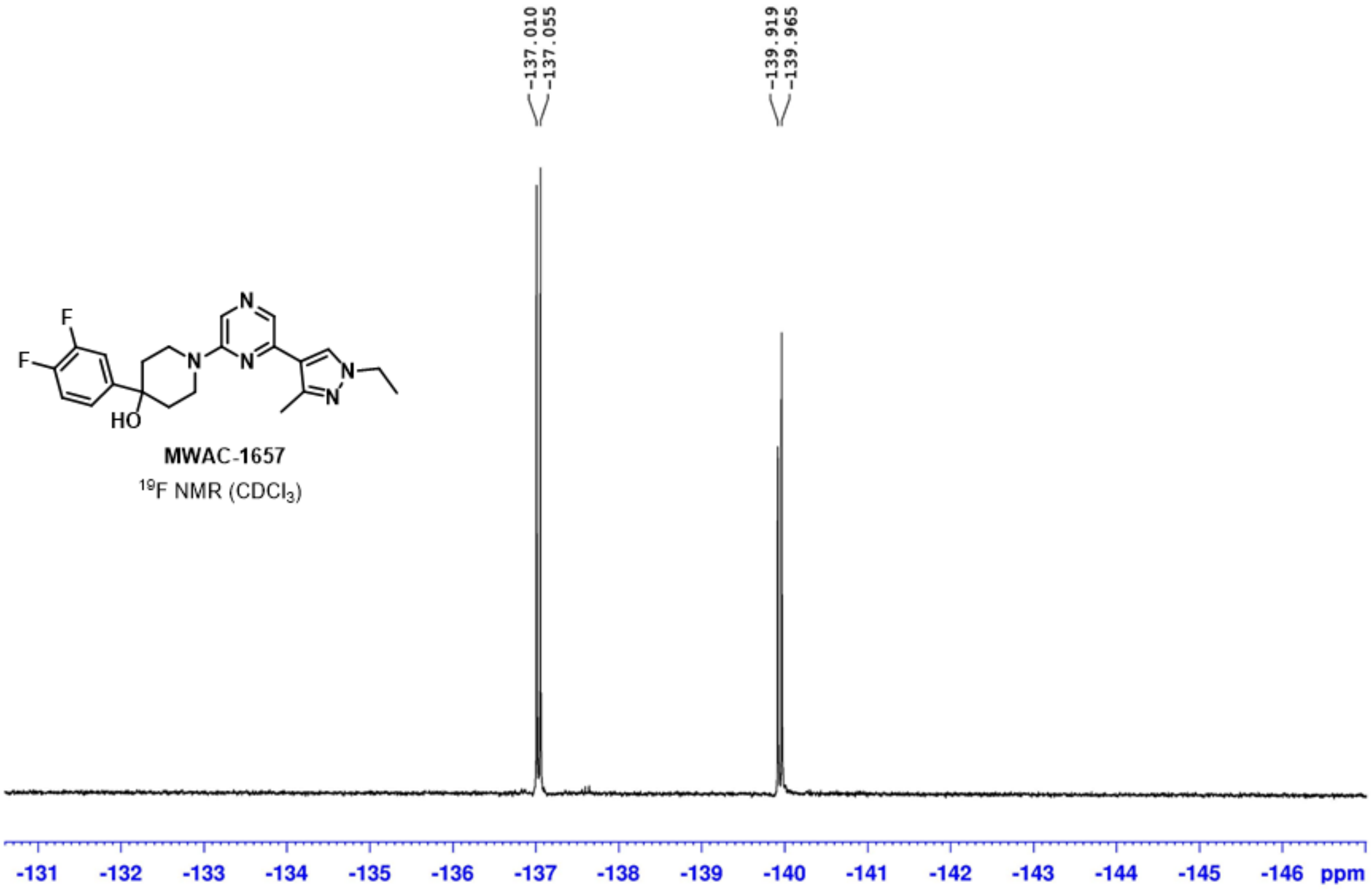

-137.010
-137.055
-139.919
-139.965
MWAC-1657
19F NMR (CDCl3)
-131 -132 -133 -134 -135 -136 -137 -138 -139 -140 -141 -142 -143 -144 -145 -146 ppm

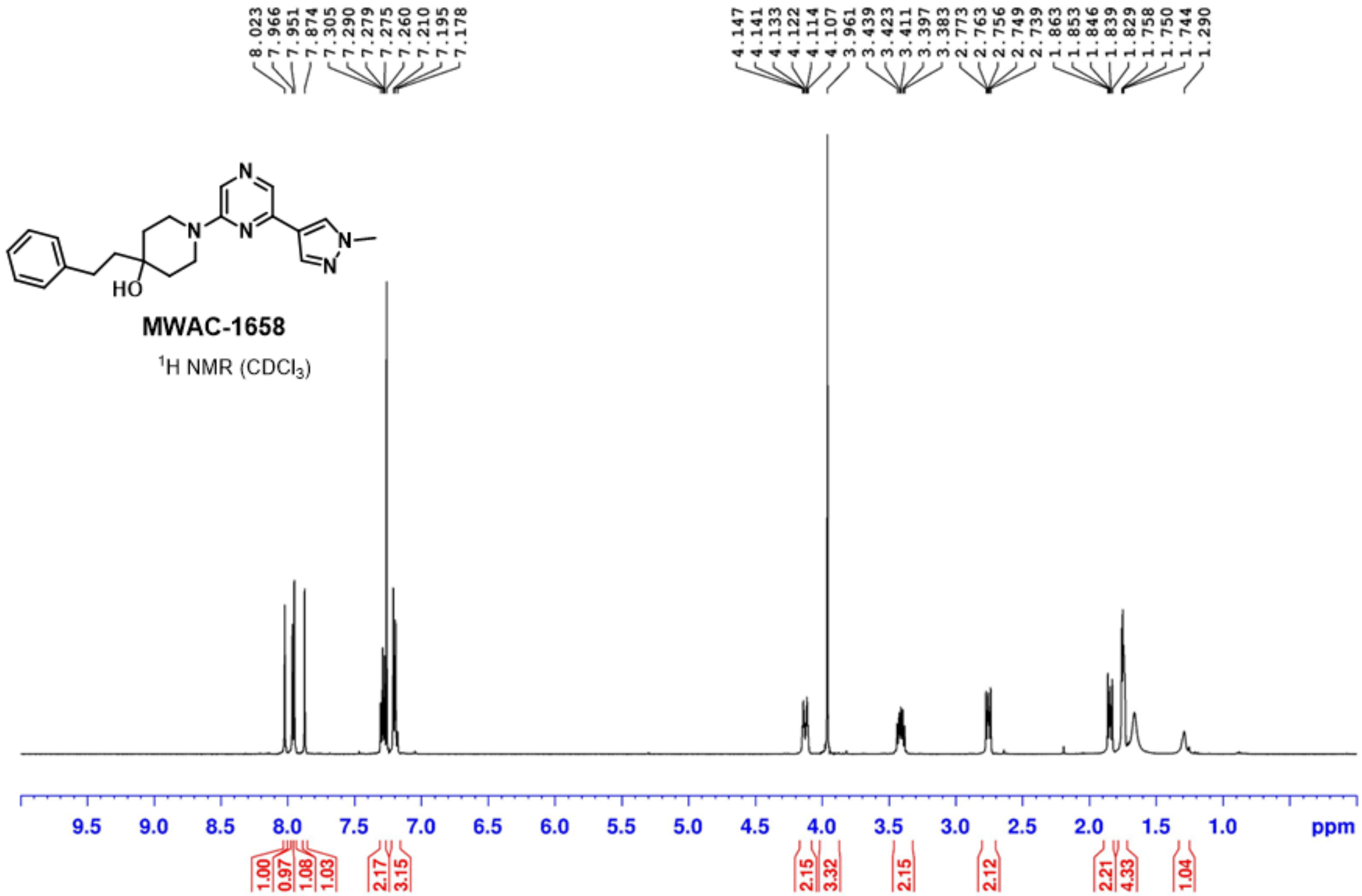
8.023
7.966
7.951
7.874
7.305
7.290
7.279
7.275
7.260
7.210
7.195
7.178
4.147
4.141
4.133
4.122
4.114
4.107
3.961
3.439
3.423
3.411
3.397
3.383
2.773
2.763
2.756
2.749
2.739
1.863
1.853
1.846
1.839
1.829
1.758
1.750
1.744
1.290
N
N
N
N
N
HO
MWAC-1658
1H NMR (CDCl3)
9.5 9.0 8.5 8.0 7.5 7.0 6.5 6.0 5.5 5.0 4.5 4.0 3.5 3.0 2.5 2.0 1.5 1.0 ppm
1.00
0.97
1.08
1.03
2.17
3.15
2.15
3.32
2.15
2.12
2.21
4.33
1.04

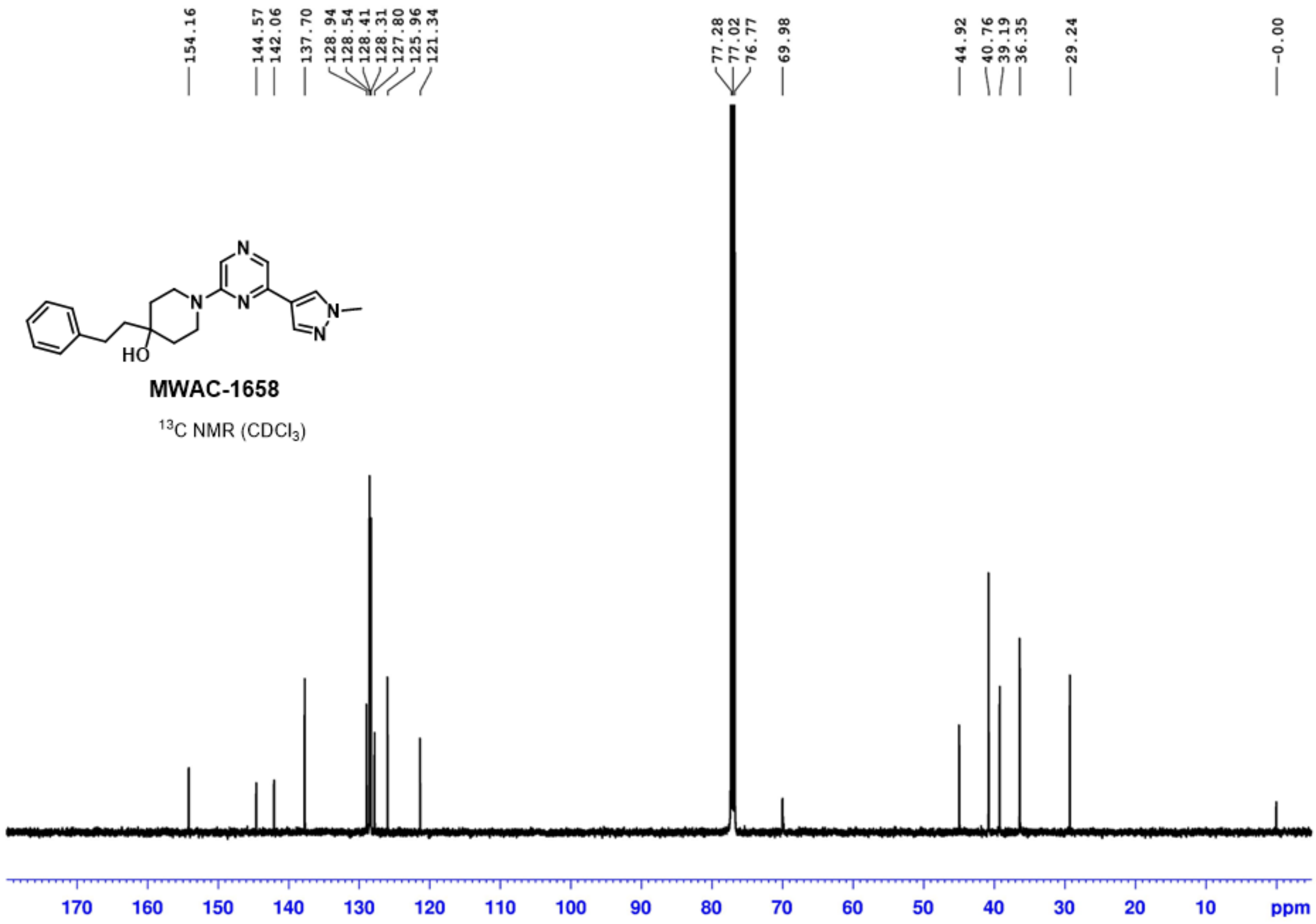
154.16
144.57
142.06
137.70
128.94
128.54
128.41
128.31
127.80
125.96
121.34
77.28
77.02
76.77
69.98
44.92
40.76
39.19
36.35
29.24
-0.00
N
N
N
N
N
HO
MWAC-1658
13C NMR (CDCl3)
170
160
150
140
130
120
110
100
90
80
70
60
50
40
30
20
10
ppm

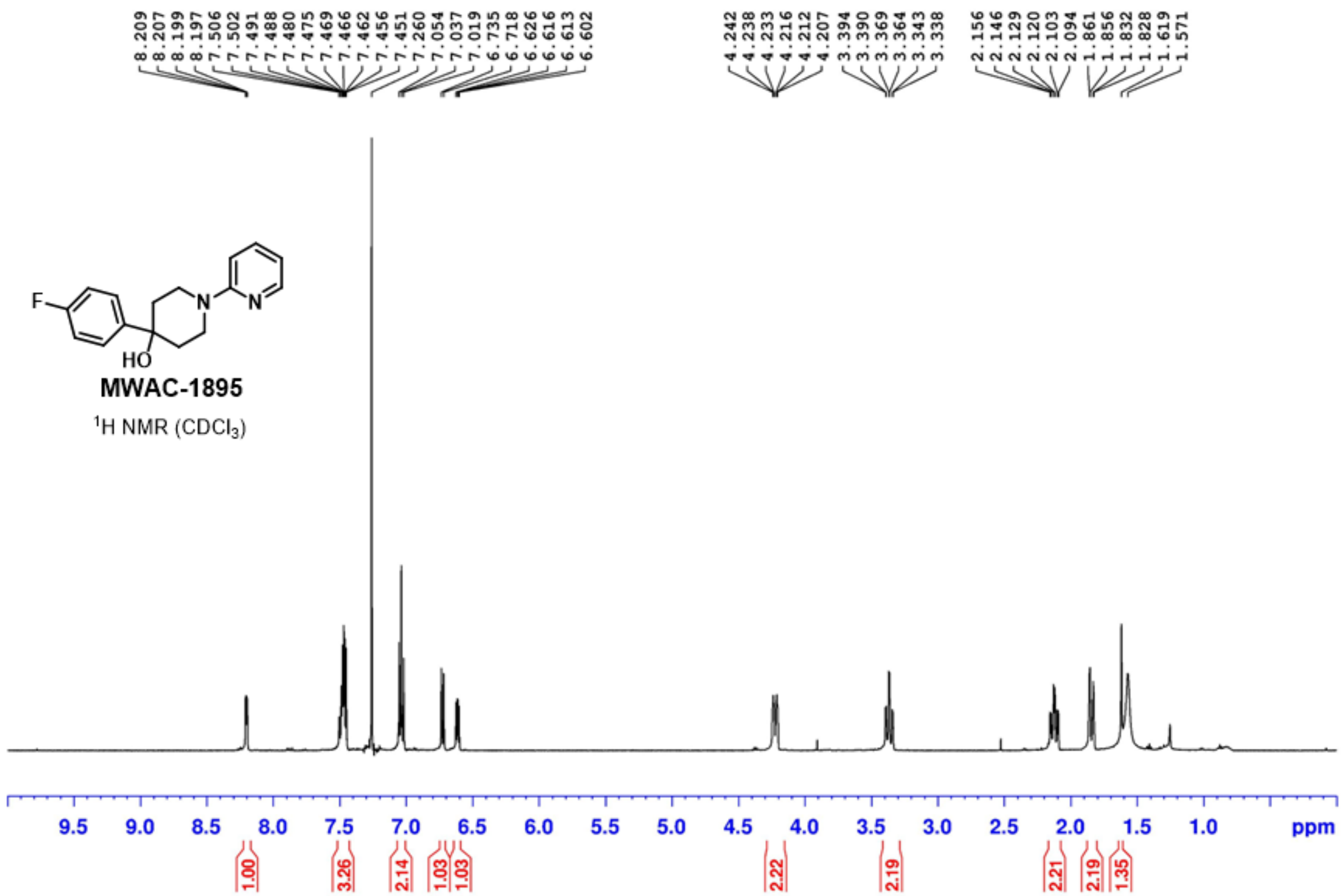

8.209
8.207
8.199
8.197
7.506
7.502
7.491
7.488
7.480
7.475
7.469
7.466
7.462
7.456
7.451
7.260
7.054
7.037
7.019
6.735
6.718
6.626
6.616
6.613
6.602
4.242
4.238
4.233
4.216
4.212
4.207
3.394
3.390
3.369
3.364
3.343
3.338
2.156
2.146
2.129
2.120
2.103
2.094
1.861
1.856
1.832
1.828
1.619
1.571
F
N
N
HO
MWAC-1895
¹H NMR (CDCl₃)
9.5
9.0
8.5
8.0
7.5
7.0
6.5
6.0
5.5
5.0
4.5
4.0
3.5
3.0
2.5
2.0
1.5
1.0
ppm
1.00
3.26
2.14
1.03
1.03
2.22
2.19
2.21
2.19
1.35

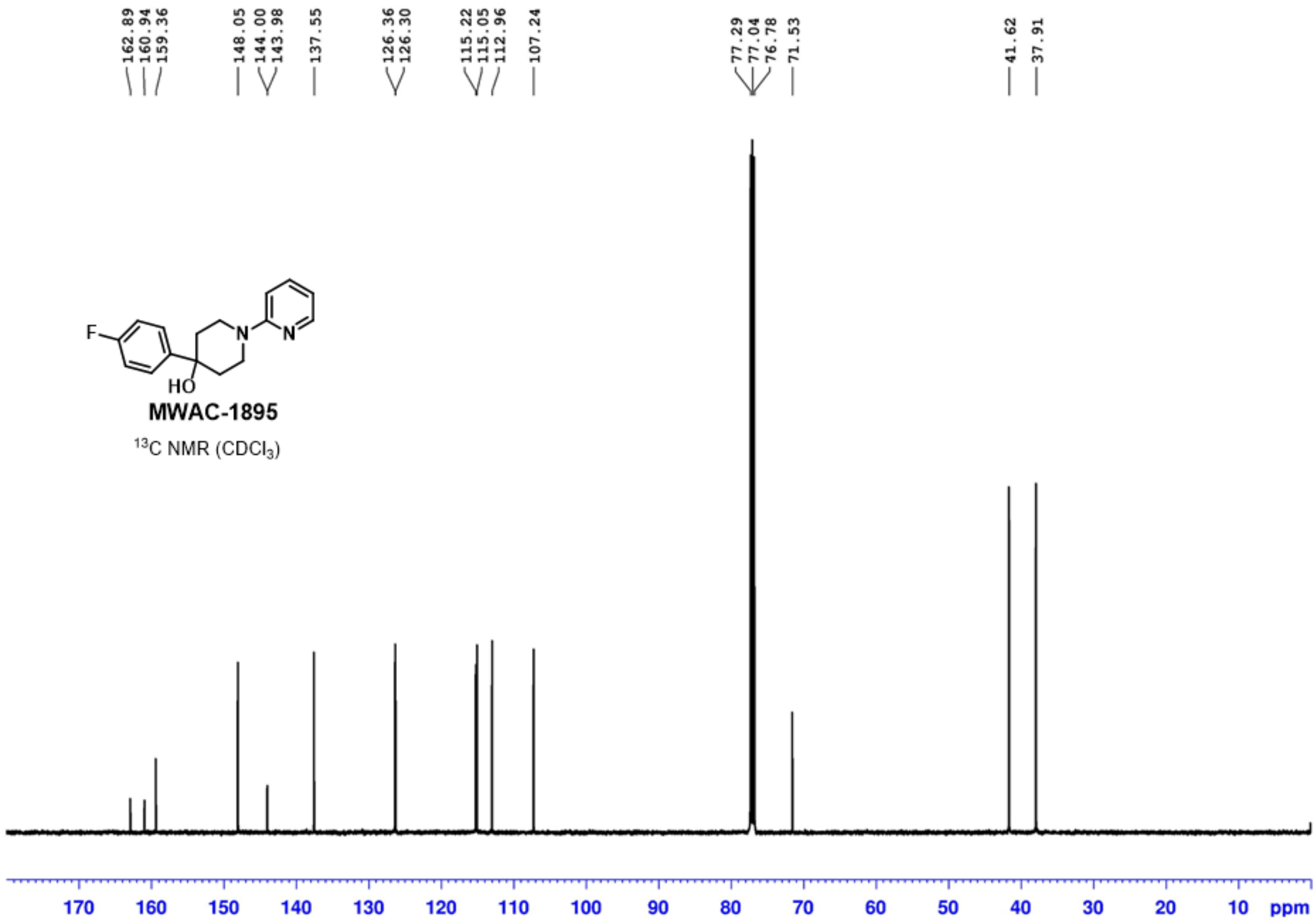
162.89
160.94
159.36
148.05
144.00
143.98
137.55
126.36
126.30
115.22
115.05
112.96
107.24
77.29
77.04
76.78
71.53
41.62
37.91
F
N
N
HO
MWAC-1895
13C NMR (CDCl3)
170
160
150
140
130
120
110
100
90
80
70
60
50
40
30
20
10
ppm

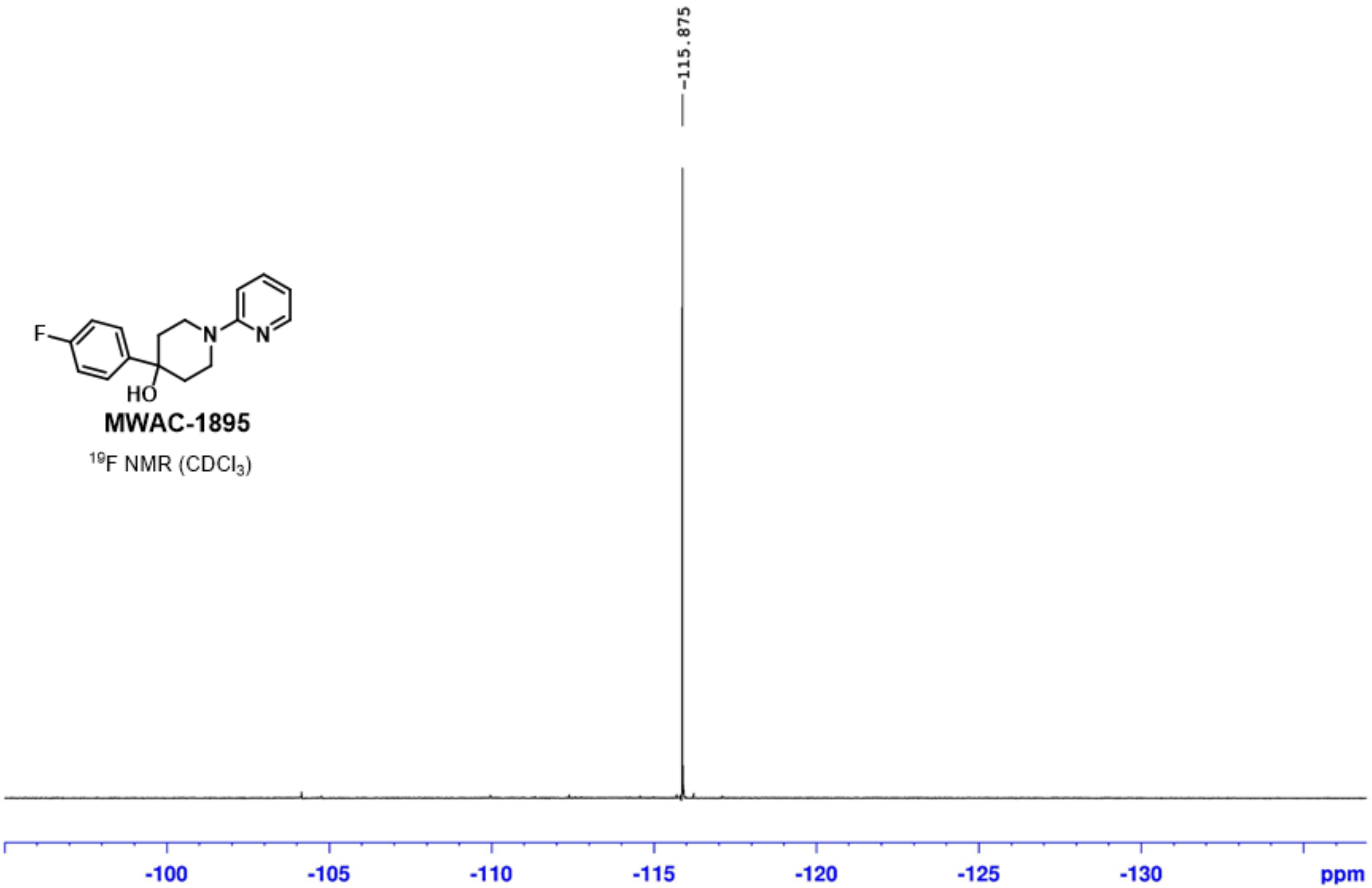

-115.875
MWAC-1895
19F NMR (CDCl3)
-100
-105
-110
-115
-120
-125
-130
ppm

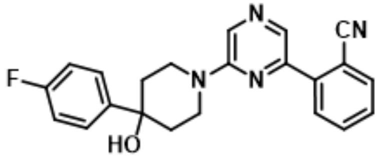

MWAC-2122

^{1}H NMR ($CDCl_3$)

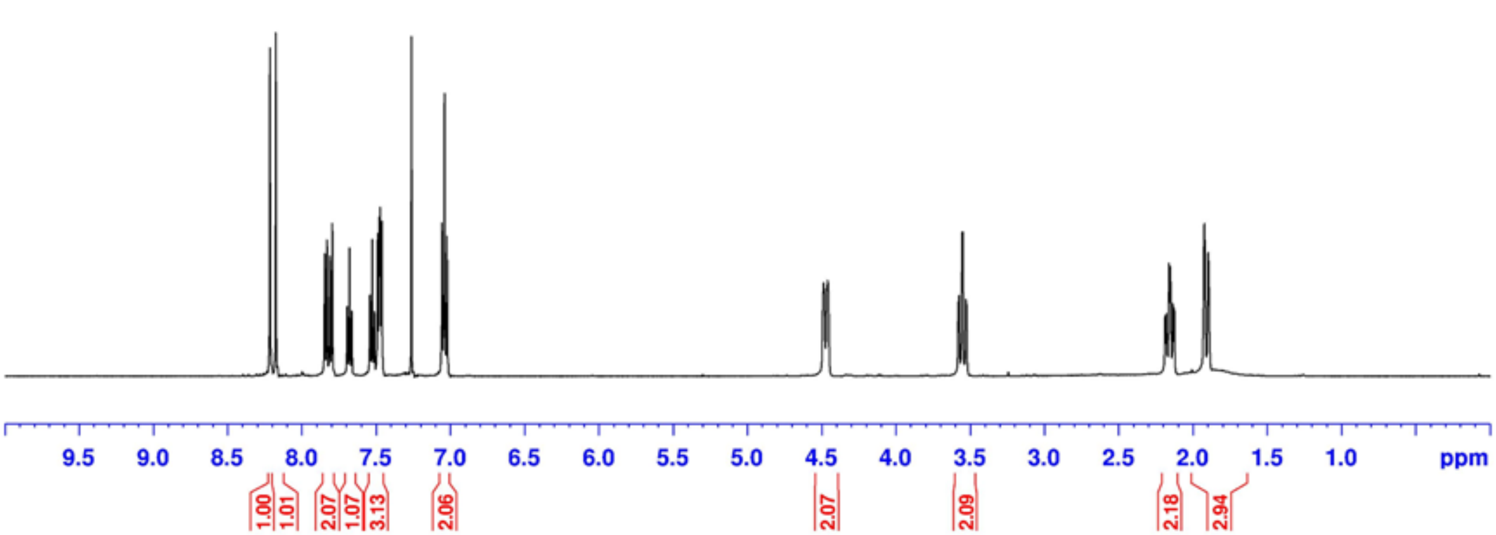

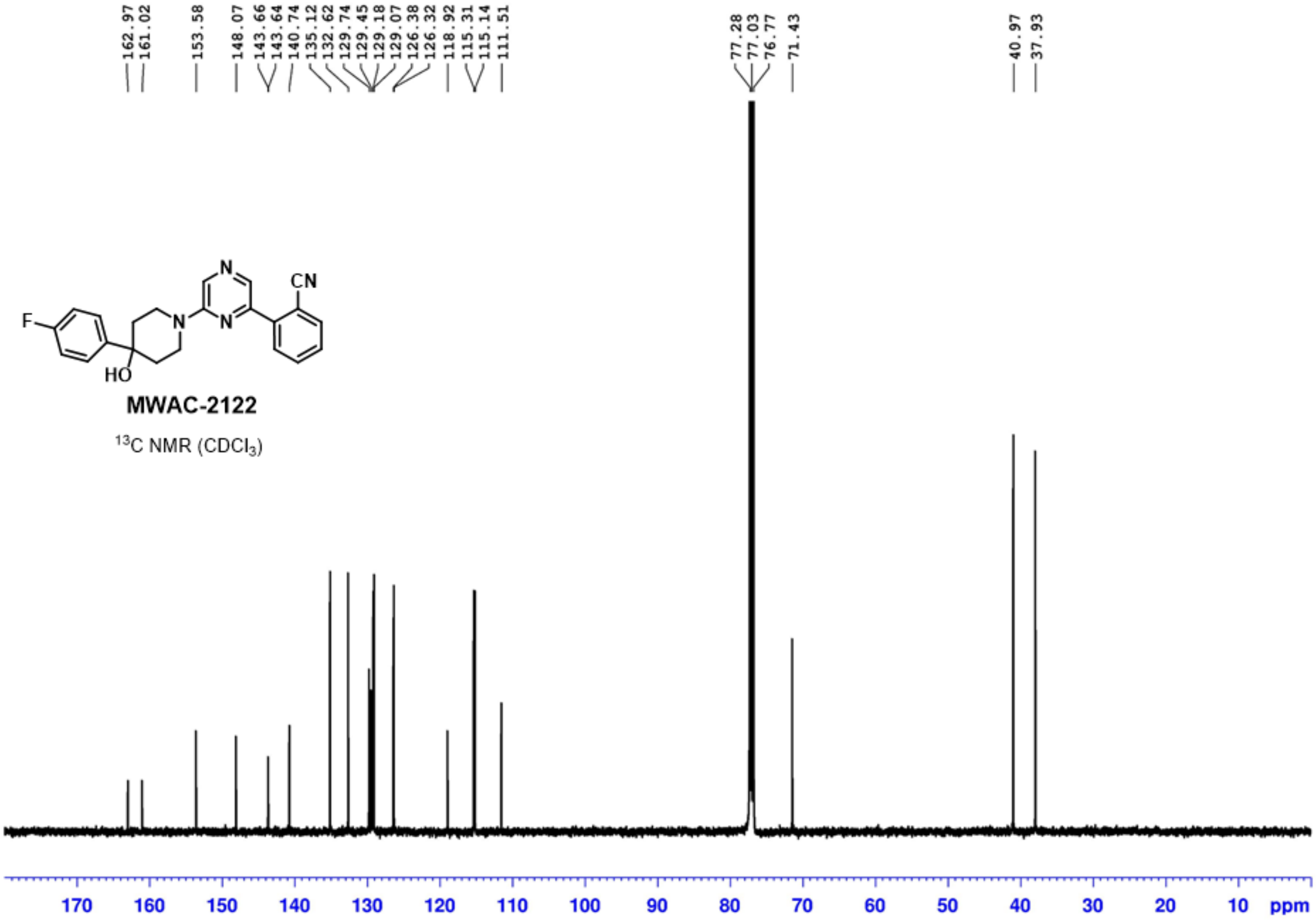

162.97
161.02
153.58
148.07
143.66
143.64
140.74
135.12
132.62
129.74
129.45
129.18
129.07
126.38
126.32
118.92
115.31
115.14
111.51
77.28
77.03
76.77
71.43
40.97
37.93
F
HO
N
N
N
CN
MWAC-2122
^{13}C NMR ($CDCl_3$)
170
160
150
140
130
120
110
100
90
80
70
60
50
40
30
20
10
ppm

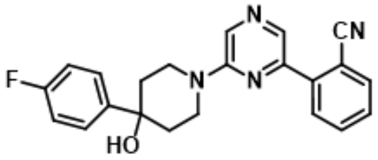

MWAC-2122

^{19}F NMR ($CDCl_3$)

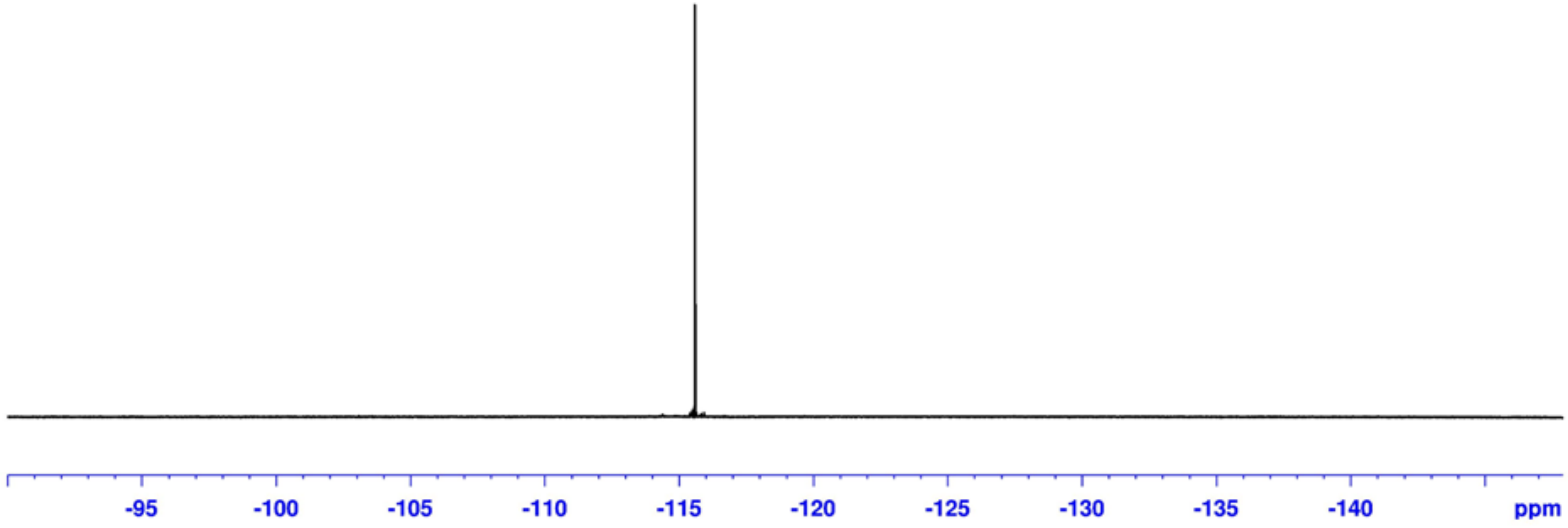

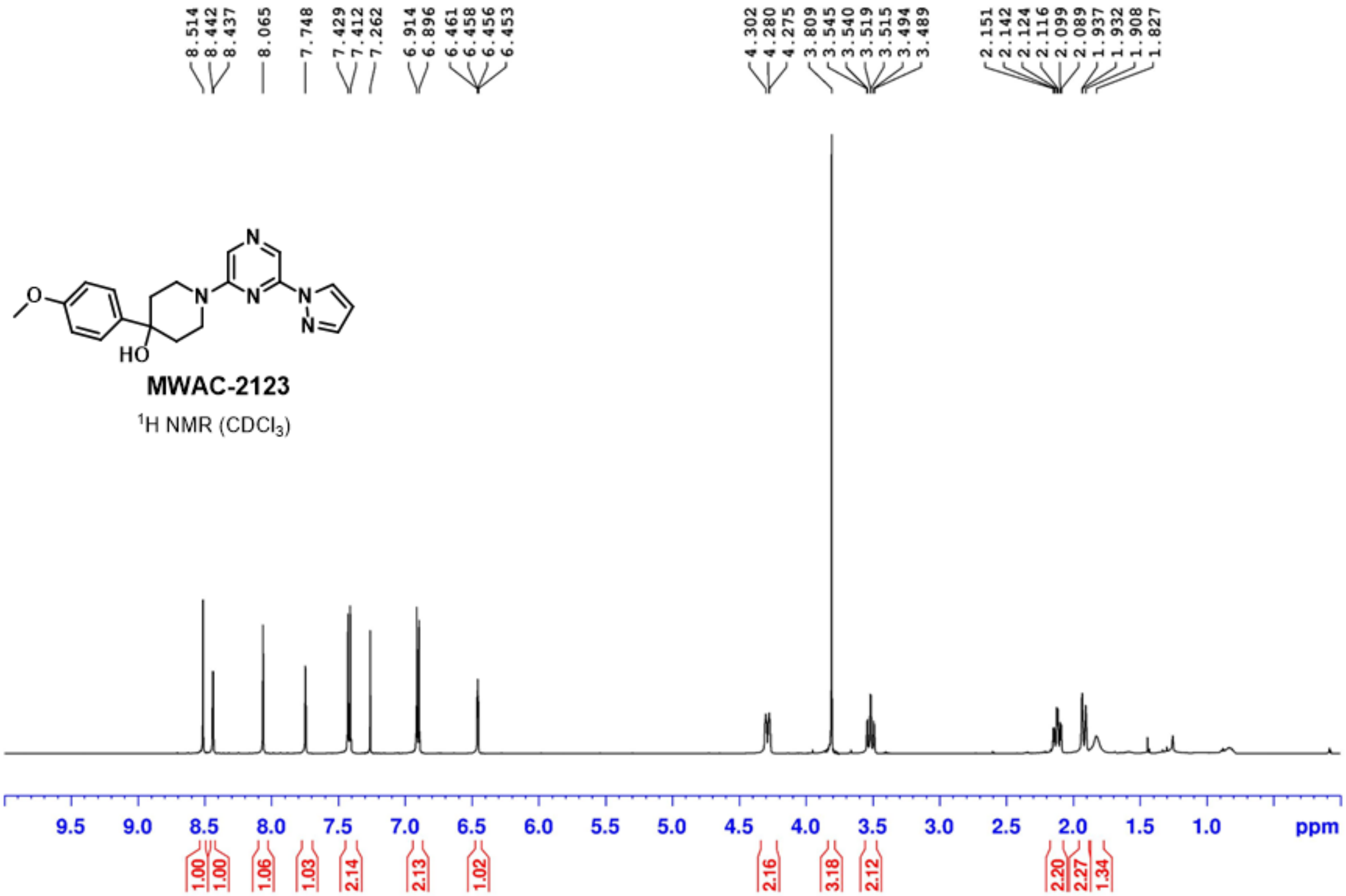
8.514
8.442
8.437
8.065
7.748
7.429
7.412
7.262
6.914
6.896
6.461
6.458
6.456
6.453
4.302
4.280
4.275
3.809
3.545
3.540
3.519
3.515
3.494
3.489
2.151
2.142
2.124
2.116
2.099
2.089
1.937
1.932
1.908
1.827
O
N
N
N
N
N
HO
MWAC-2123
1H NMR (CDCl3)
9.5 9.0 8.5 8.0 7.5 7.0 6.5 6.0 5.5 5.0 4.5 4.0 3.5 3.0 2.5 2.0 1.5 1.0 ppm
1.00
1.00
1.06
1.03
2.14
2.13
1.02
2.16
3.18
2.12
2.20
2.27
1.34

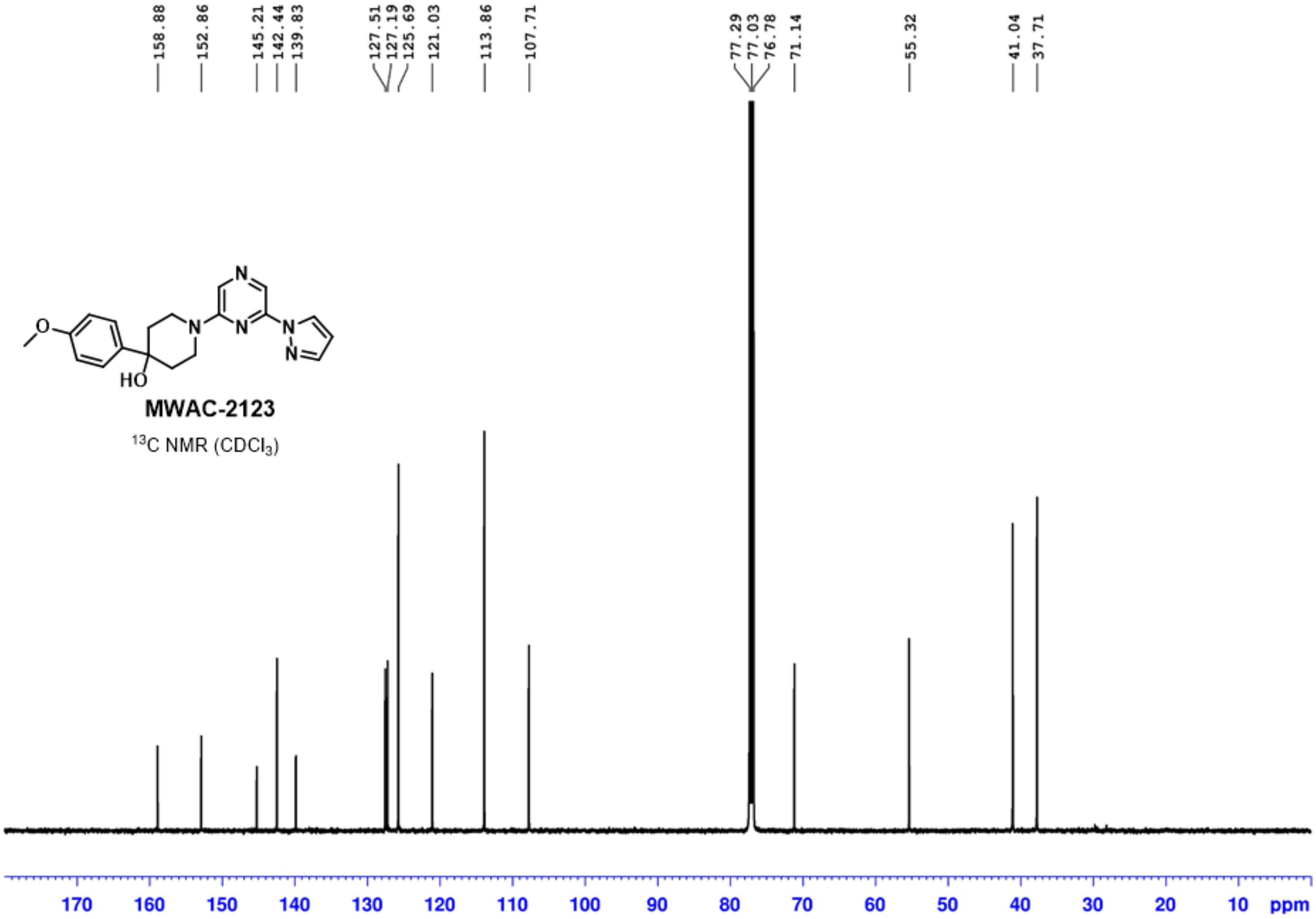
158.88
152.86
145.21
142.44
139.83
127.51
127.19
125.69
121.03
113.86
107.71
77.29
77.03
76.78
71.14
55.32
41.04
37.71
O
N
N
N
N
N
HO
MWAC-2123
13C NMR (CDCl3)
170 160 150 140 130 120 110 100 90 80 70 60 50 40 30 20 10 ppm

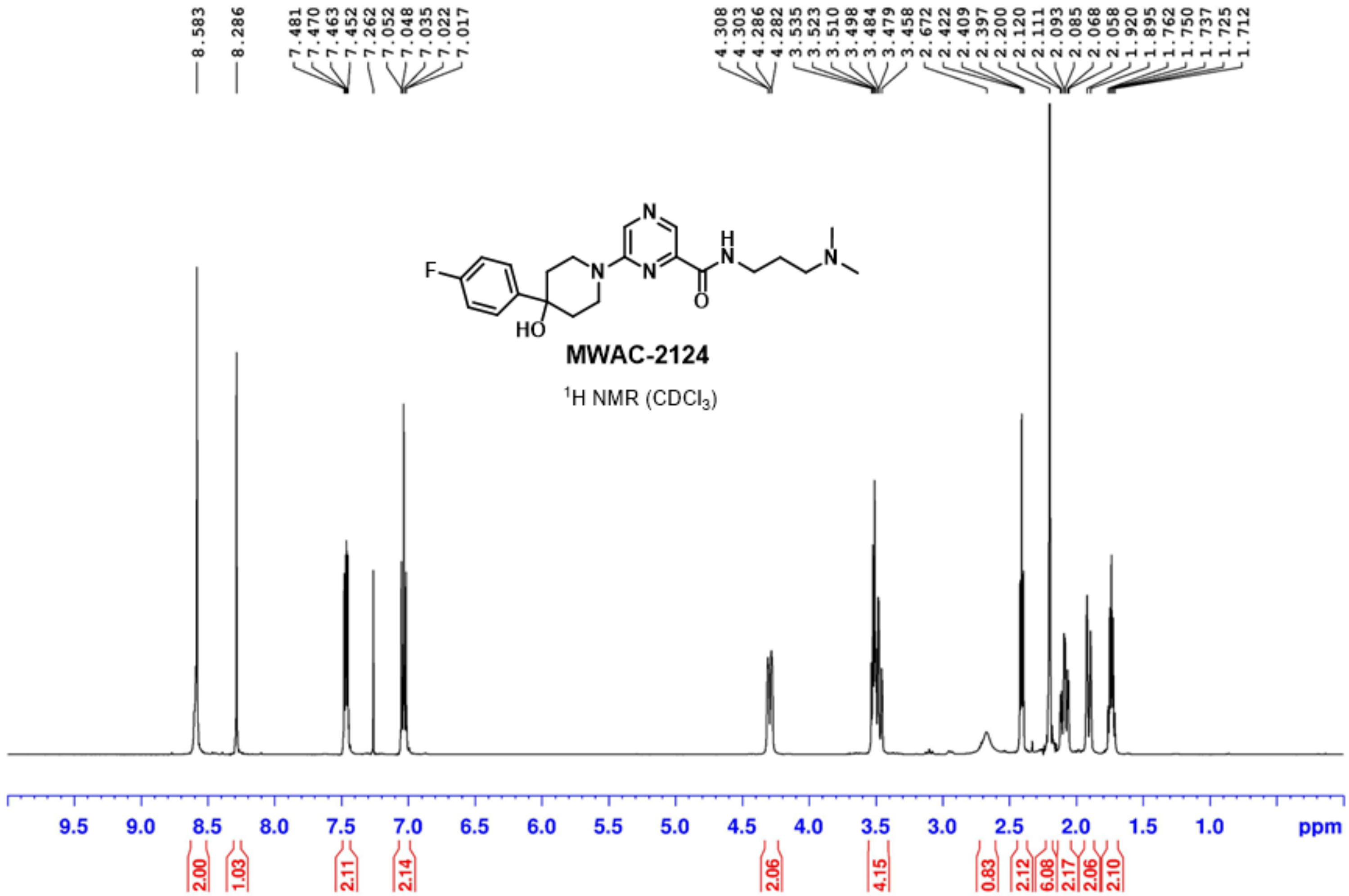
8.583
8.286
7.481
7.470
7.463
7.452
7.262
7.052
7.048
7.035
7.022
7.017
4.308
4.303
4.286
4.282
3.535
3.523
3.510
3.498
3.484
3.479
3.458
2.672
2.422
2.409
2.397
2.200
2.120
2.111
2.093
2.085
2.068
2.058
1.920
1.895
1.762
1.750
1.737
1.725
1.712
MWAC-2124
1H NMR (CDCl3)
9.5 9.0 8.5 8.0 7.5 7.0 6.5 6.0 5.5 5.0 4.5 4.0 3.5 3.0 2.5 2.0 1.5 1.0 ppm
2.00
1.03
2.11
2.14
2.06
4.15
0.83
2.12
6.08
2.17
2.06
2.10

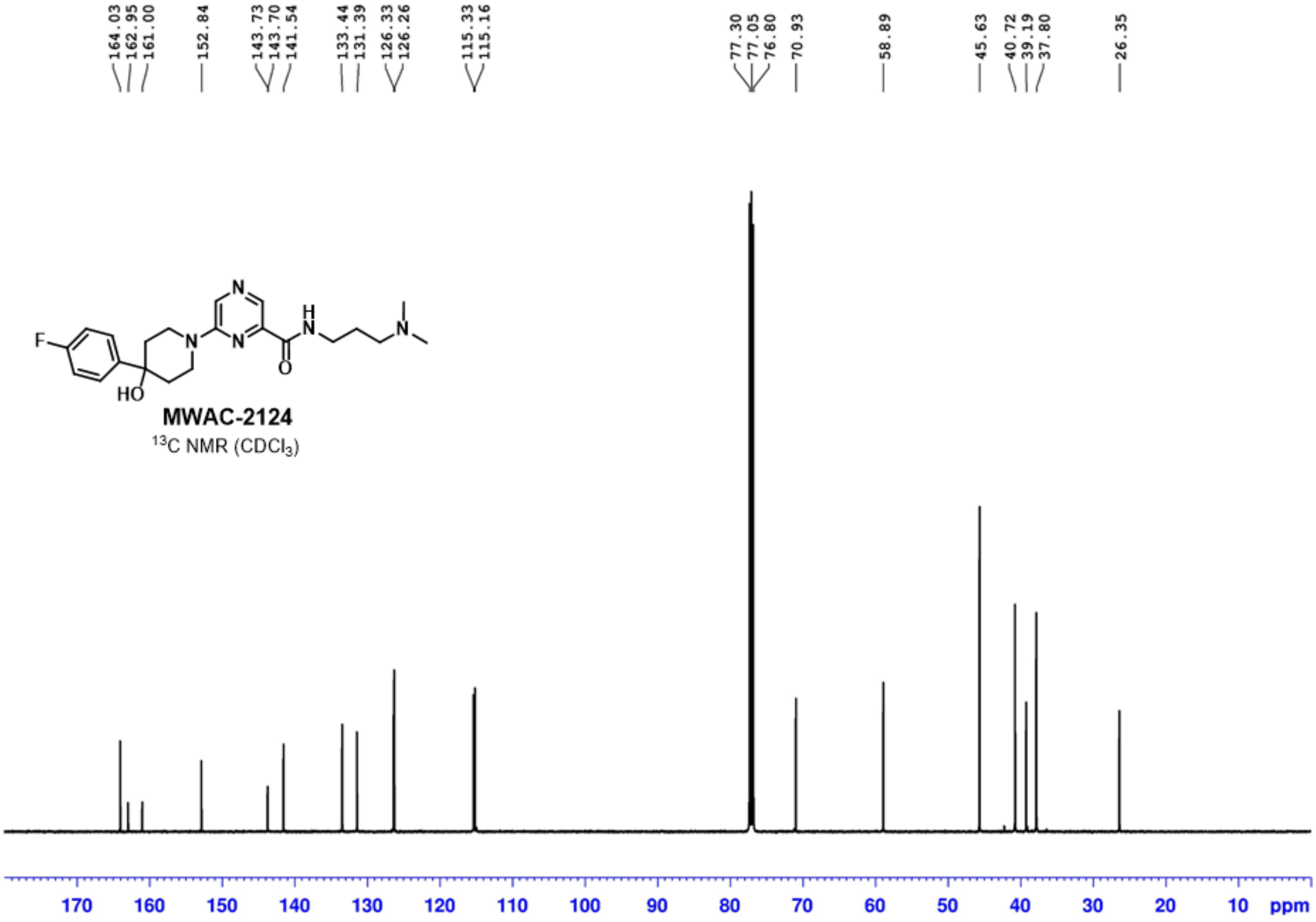
164.03
162.95
161.00
152.84
143.73
143.70
141.54
133.44
131.39
126.33
126.26
115.33
115.16
77.30
77.05
76.80
70.93
58.89
45.63
40.72
39.19
37.80
26.35
MWAC-2124
^{13}C NMR ($CDCl_3$)
170 160 150 140 130 120 110 100 90 80 70 60 50 40 30 20 10 ppm

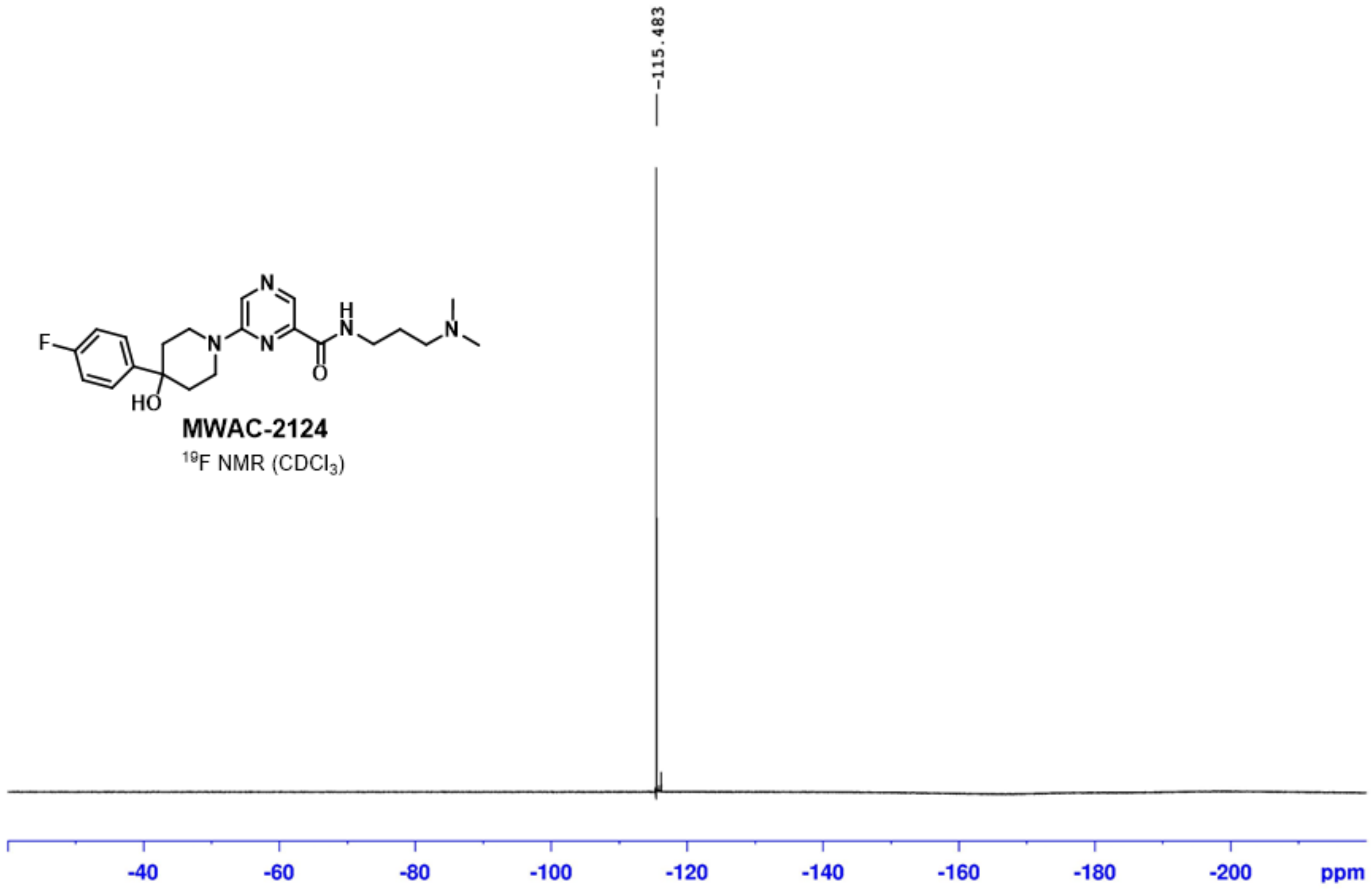
-115.483
MWAC-2124
19F NMR (CDCl3)
-40
-60
-80
-100
-120
-140
-160
-180
-200
ppm

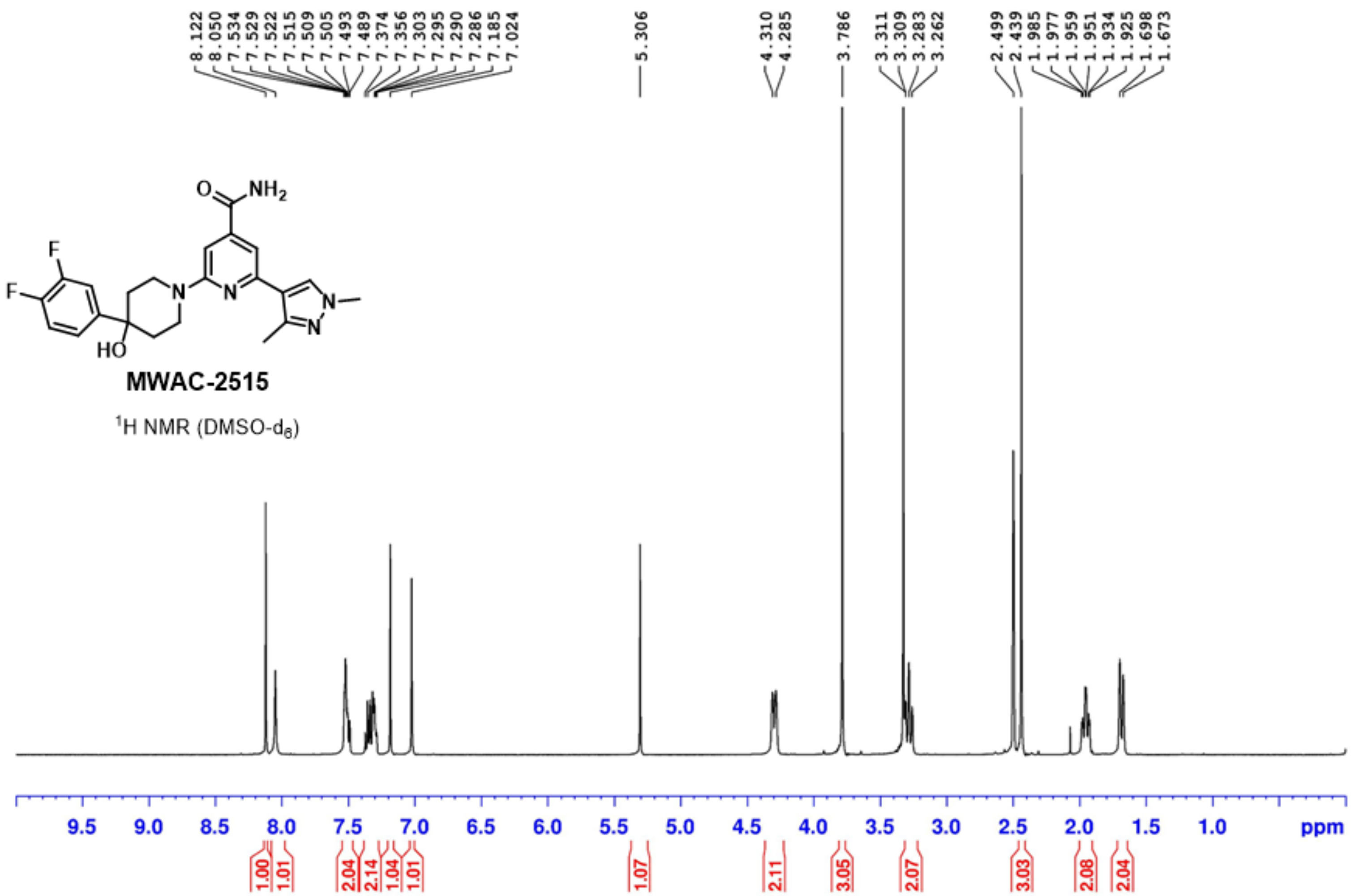

MWAC-2515
^{1}H NMR (DMSO-d$_6$)
8.122
8.050
7.534
7.529
7.522
7.515
7.509
7.505
7.493
7.489
7.374
7.356
7.303
7.295
7.290
7.286
7.185
7.024
5.306
4.310
4.285
3.786
3.311
3.309
3.283
3.262
2.499
2.439
1.985
1.977
1.959
1.951
1.934
1.925
1.698
1.673
9.5 9.0 8.5 8.0 7.5 7.0 6.5 6.0 5.5 5.0 4.5 4.0 3.5 3.0 2.5 2.0 1.5 1.0 ppm
1.00 1.01 2.04 2.14 1.04 1.01 1.07 2.11 3.05 2.07 3.03 2.08 2.04

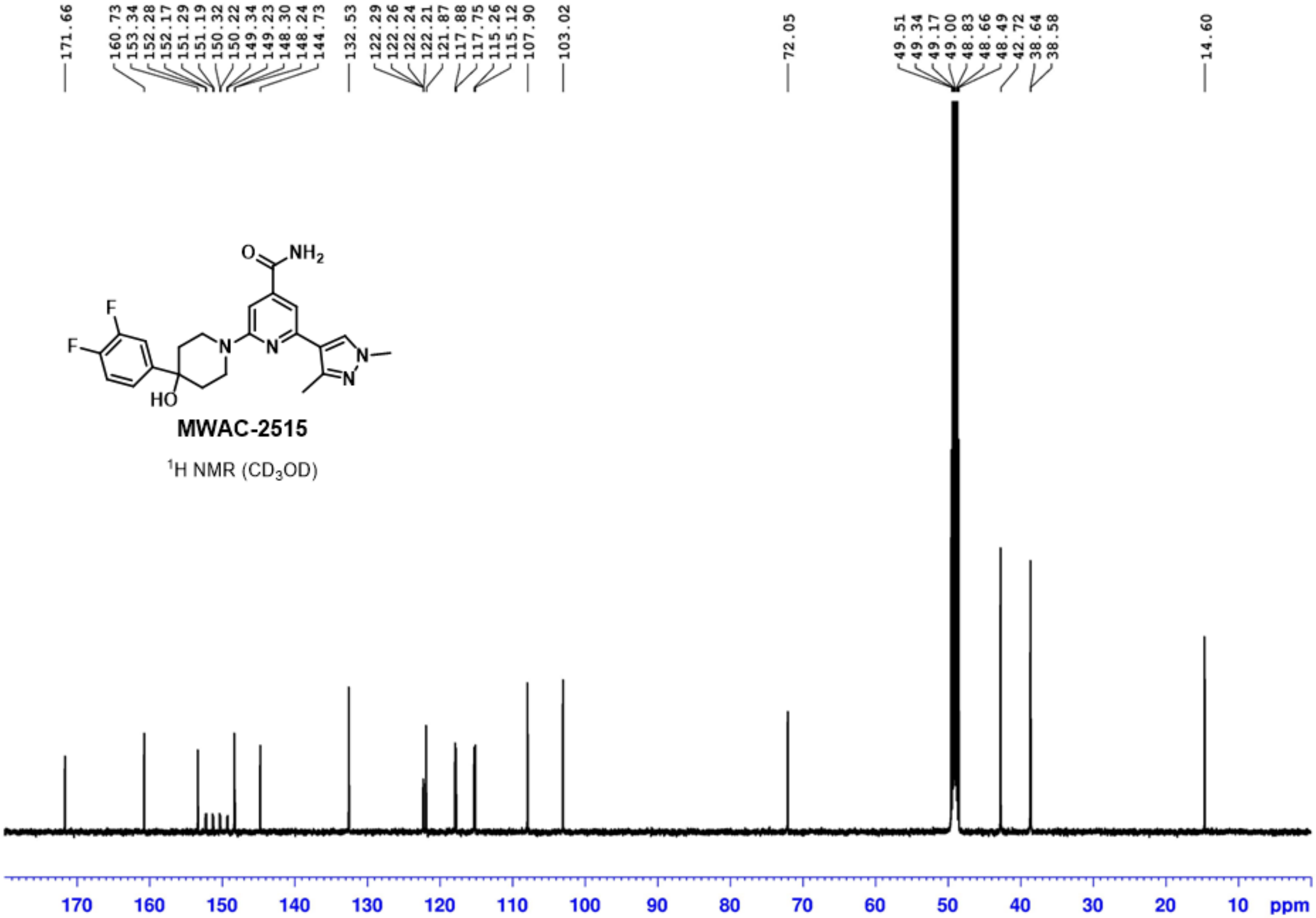

171.66
160.73
153.34
152.28
152.17
151.29
151.19
150.32
150.22
149.34
149.23
148.30
148.24
144.73
132.53
122.29
122.26
122.24
122.21
121.87
117.88
117.75
115.26
115.12
107.90
103.02
72.05
49.51
49.34
49.17
49.00
48.83
48.66
48.49
42.72
38.64
38.58
14.60
O
NH2
F
F
N
N
N
N
HO
MWAC-2515
1H NMR (CD3OD)
170
160
150
140
130
120
110
100
90
80
70
60
50
40
30
20
10
ppm

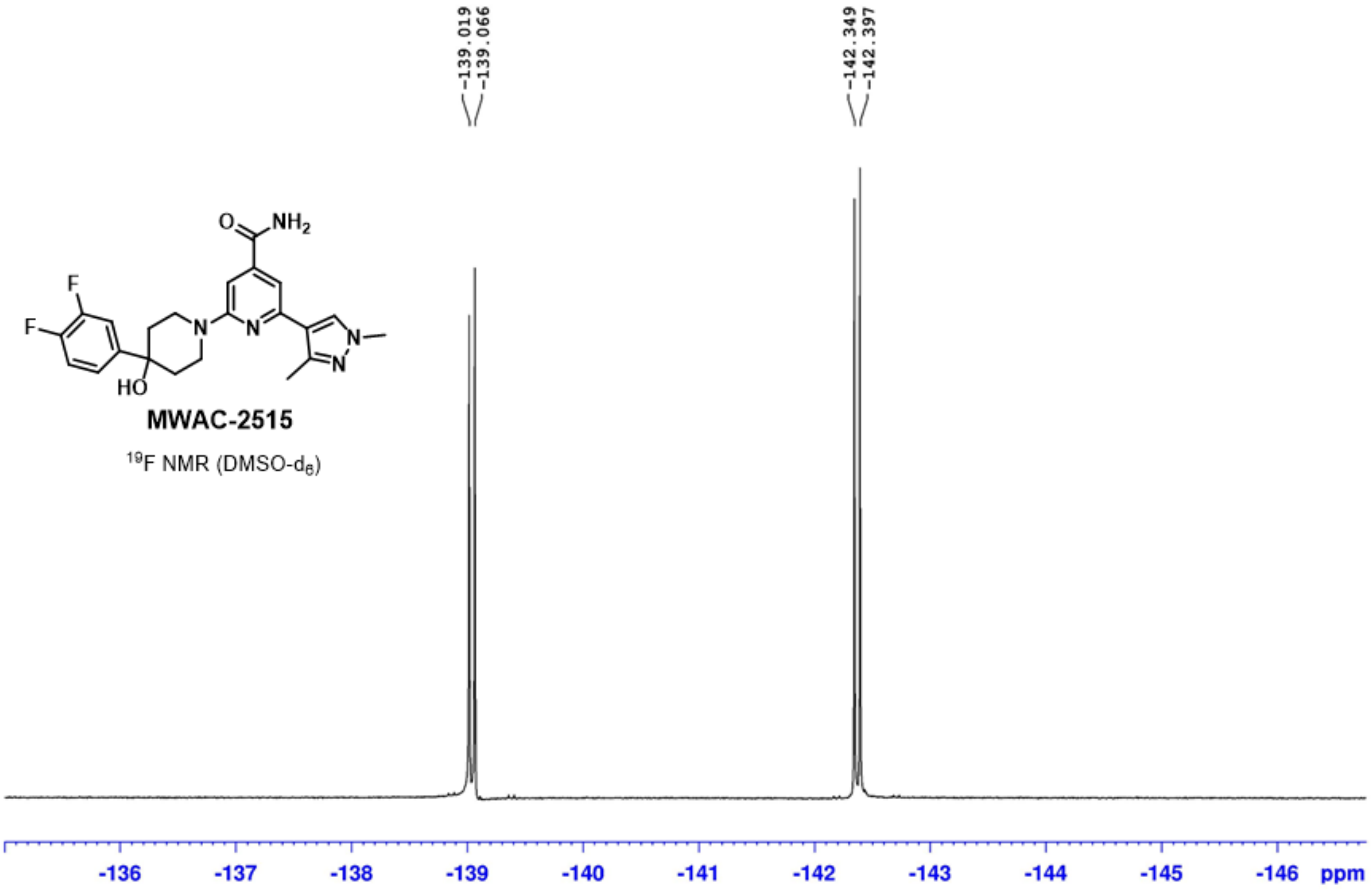
O
NH2
F
F
N
N
N
N
HO
MWAC-2515
19F NMR (DMSO-d6)
-139.019
-139.066
-142.349
-142.397
-136
-137
-138
-139
-140
-141
-142
-143
-144
-145
-146
ppm

HPLC Purity Traces

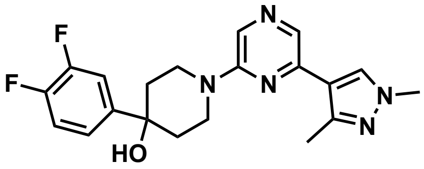

MWAC-1655

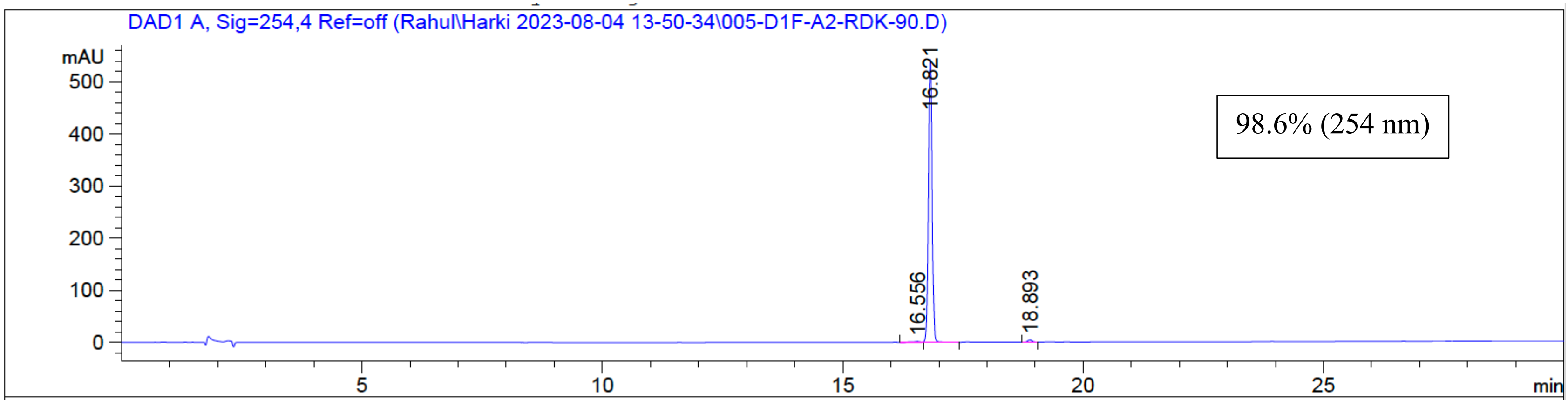

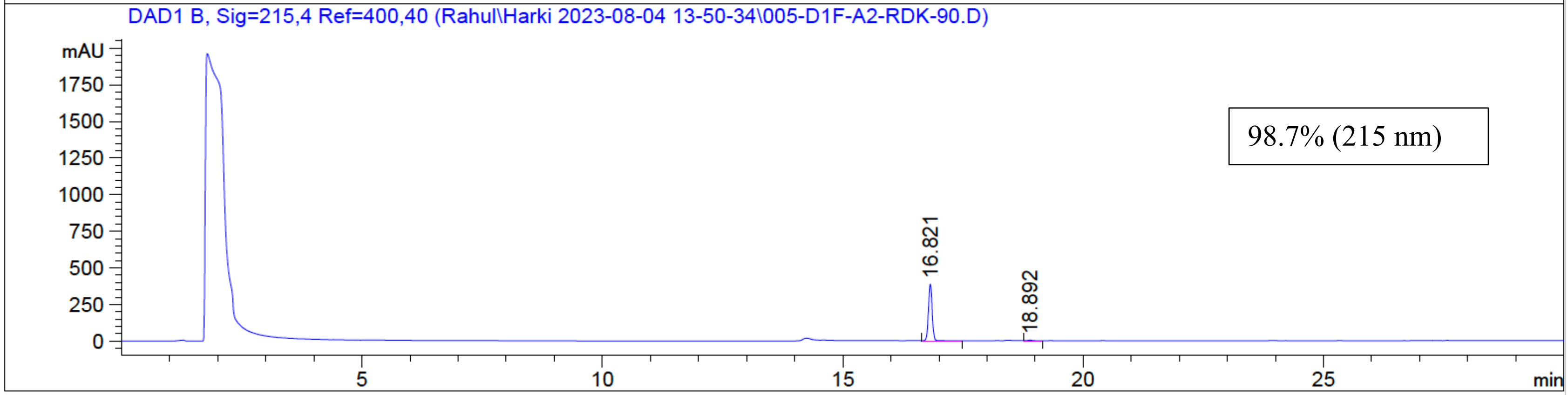

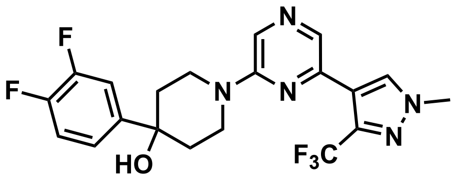
F
F
HO
N
N
N
F3C
N
N

MWAC-1656

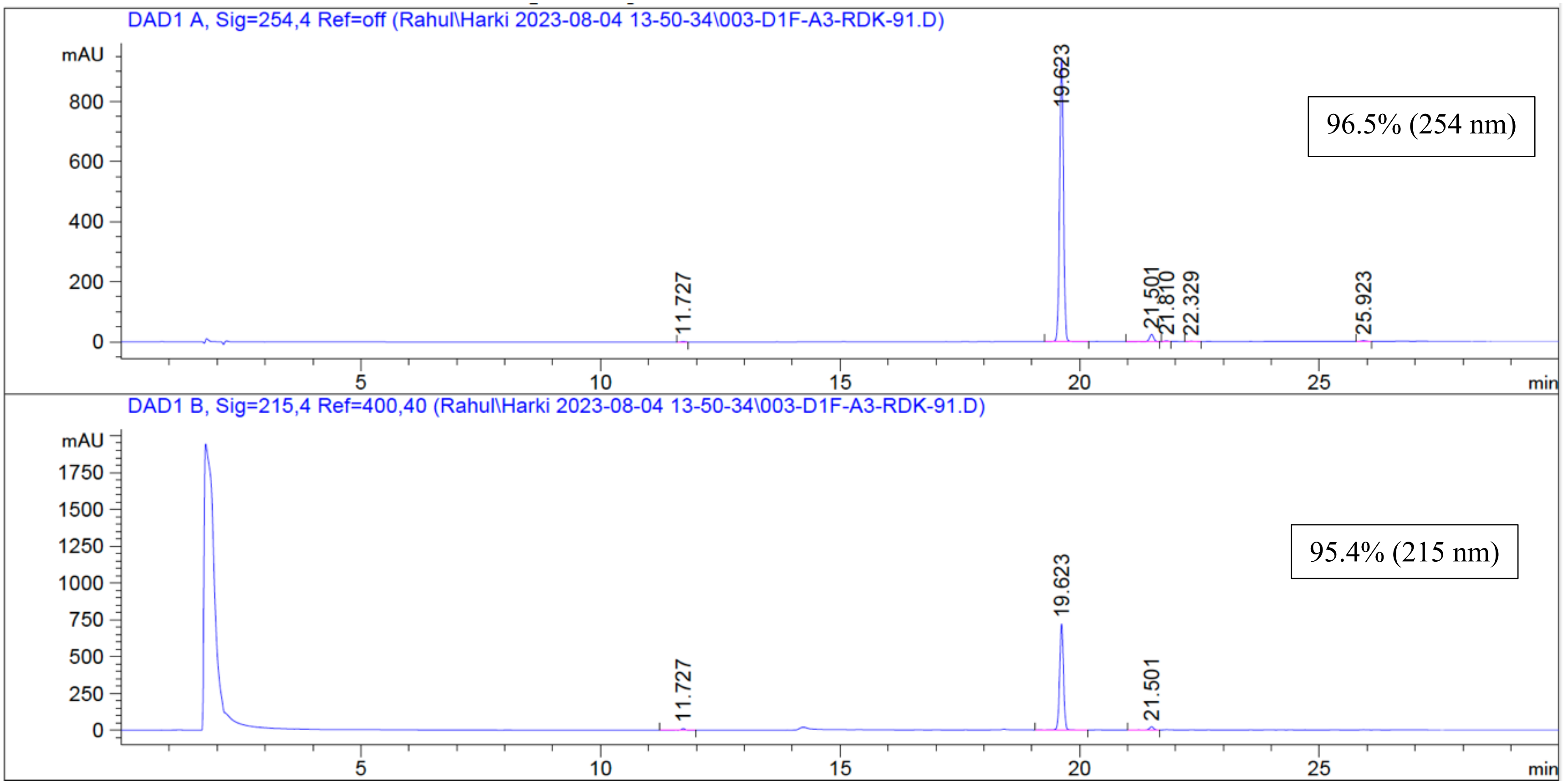
DAD1 A, Sig=254,4 Ref=off (Rahul\Harki 2023-08-04 13-50-34\003-D1F-A3-RDK-91.D)
mAU
800
600
400
200
0
19.623
11.727
21.501
21.810
22.329
25.923
96.5% (254 nm)
5
10
15
20
25
min
DAD1 B, Sig=215,4 Ref=400,40 (Rahul\Harki 2023-08-04 13-50-34\003-D1F-A3-RDK-91.D)
mAU
1750
1500
1250
1000
750
500
250
0
95.4% (215 nm)
19.623
11.727
21.501
5
10
15
20
25
min

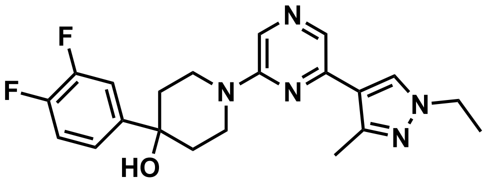
F
F
HO
N
N
N
N
N

MWAC-1657

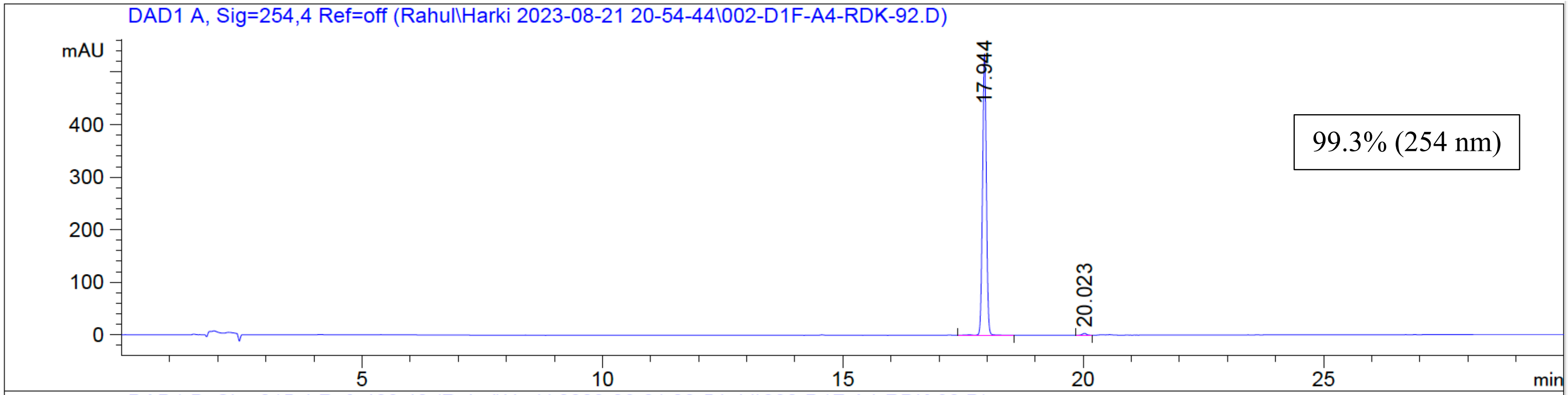
DAD1 A, Sig=254,4 Ref=off (Rahul\Harki 2023-08-21 20-54-44\002-D1F-A4-RDK-92.D)
mAU
400
300
200
100
0
17.944
20.023
99.3% (254 nm)
5
10
15
20
25
min

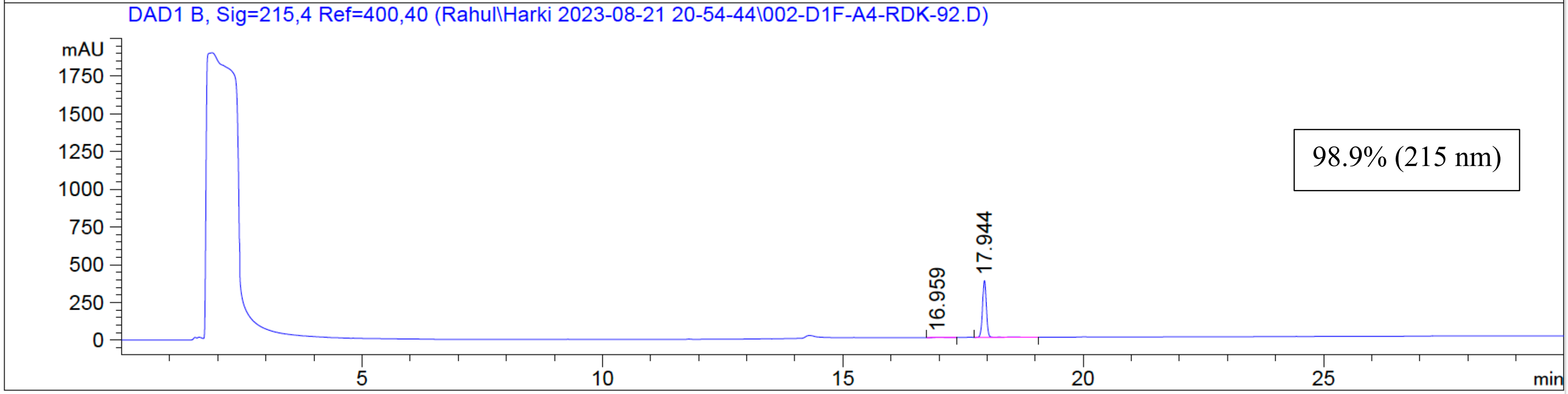
DAD1 B, Sig=215,4 Ref=400,40 (Rahul\Harki 2023-08-21 20-54-44\002-D1F-A4-RDK-92.D)
mAU
1750
1500
1250
1000
750
500
250
0
16.959
17.944
98.9% (215 nm)
5
10
15
20
25
min

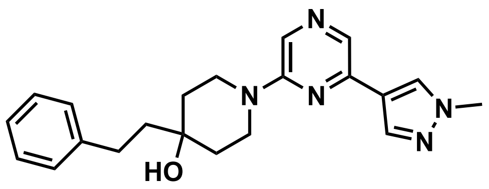
N
N
N
N
N
HO

MWAC-1658

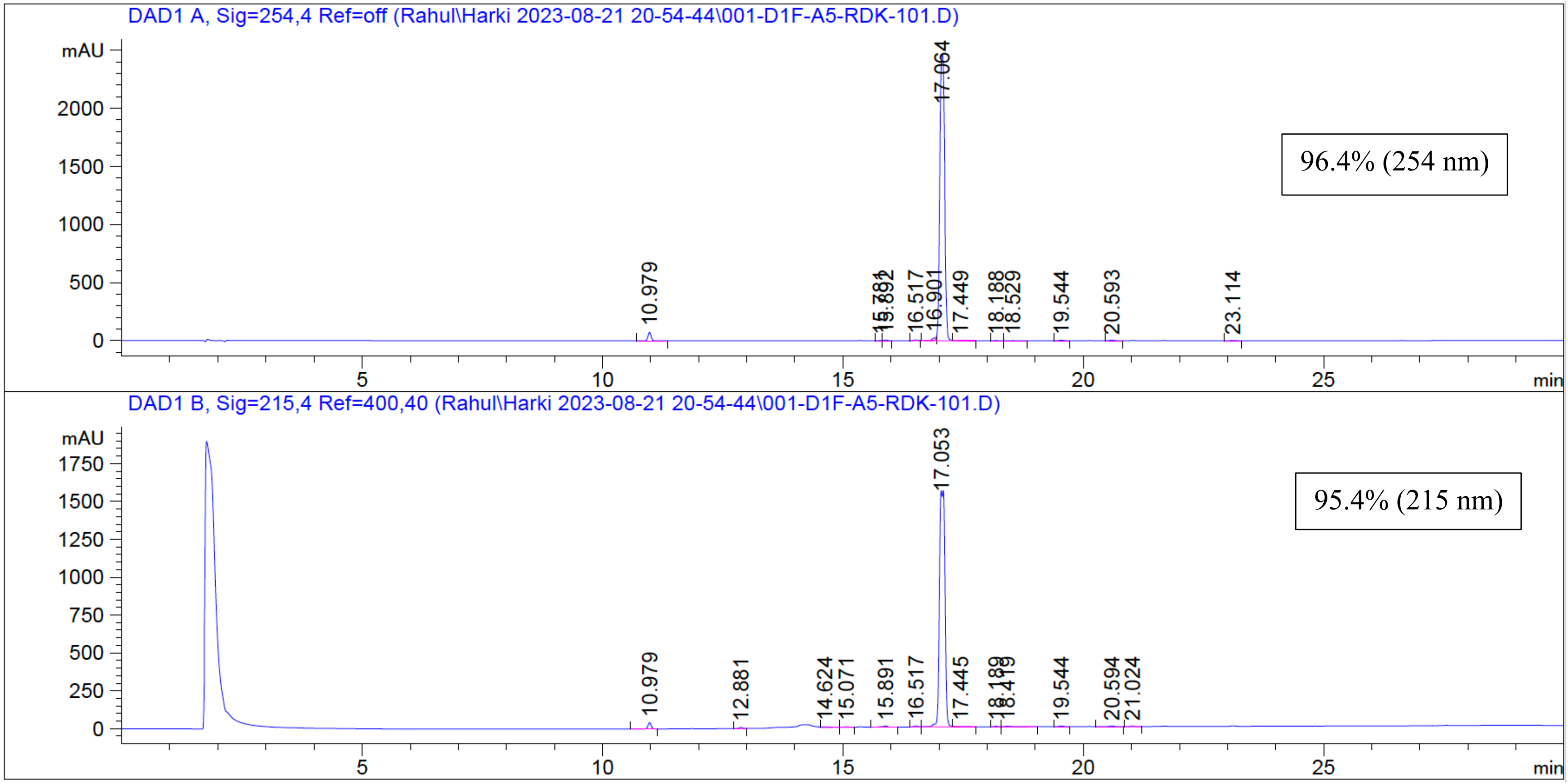
DAD1 A, Sig=254,4 Ref=off (Rahul\Harki 2023-08-21 20-54-44\001-D1F-A5-RDK-101.D)
mAU
2000
1500
1000
500
0
10.979
16.517
16.901
17.064
17.449
18.188
18.529
19.544
20.593
23.114
96.4% (254 nm)
5
10
15
20
25
min
DAD1 B, Sig=215,4 Ref=400,40 (Rahul\Harki 2023-08-21 20-54-44\001-D1F-A5-RDK-101.D)
mAU
1750
1500
1250
1000
750
500
250
0
10.979
12.881
14.624
15.071
15.891
16.517
17.053
17.445
19.544
20.594
21.024
95.4% (215 nm)
5
10
15
20
25
min

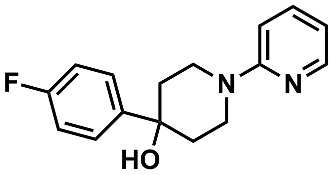
F
N
N
HO

MWAC-1895

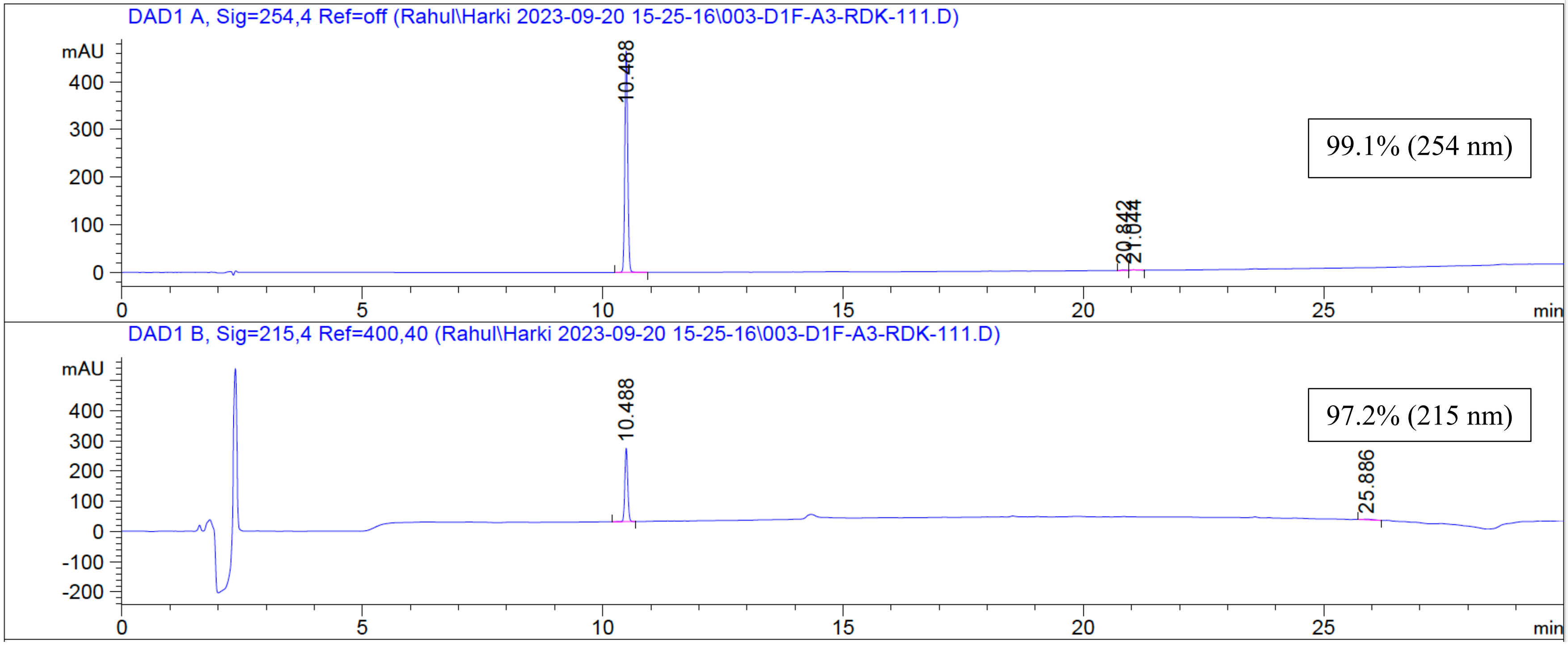
DAD1 A, Sig=254,4 Ref=off (Rahul\Harki 2023-09-20 15-25-16\003-D1F-A3-RDK-111.D)
mAU
400
300
200
100
0
10.488
20.842
21.044
99.1% (254 nm)
0
5
10
15
20
25
min
DAD1 B, Sig=215,4 Ref=400,40 (Rahul\Harki 2023-09-20 15-25-16\003-D1F-A3-RDK-111.D)
mAU
400
300
200
100
0
-100
-200
10.488
25.886
97.2% (215 nm)
0
5
10
15
20
25
min

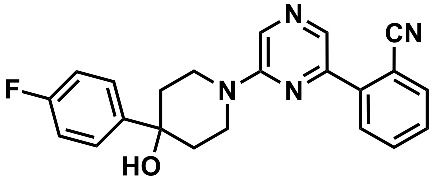
F
HO
N
N
N
CN

MWAC-2122

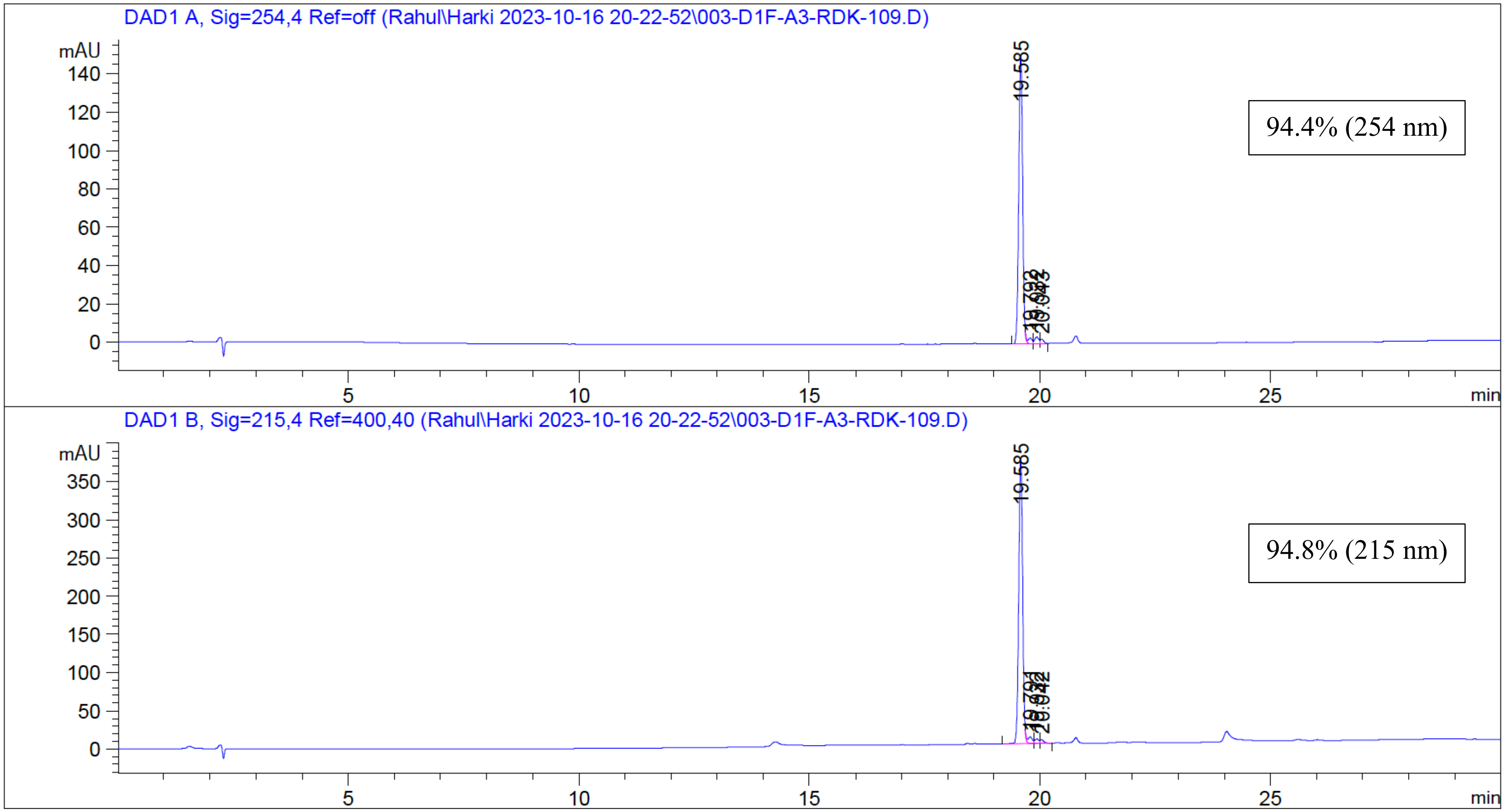
DAD1 A, Sig=254,4 Ref=off (Rahul\Harki 2023-10-16 20-22-52\003-D1F-A3-RDK-109.D)
mAU
19.585
94.4% (254 nm)
min
DAD1 B, Sig=215,4 Ref=400,40 (Rahul\Harki 2023-10-16 20-22-52\003-D1F-A3-RDK-109.D)
mAU
19.585
94.8% (215 nm)
min

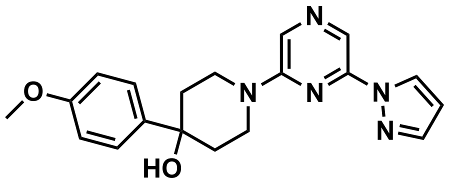

MWAC-2123

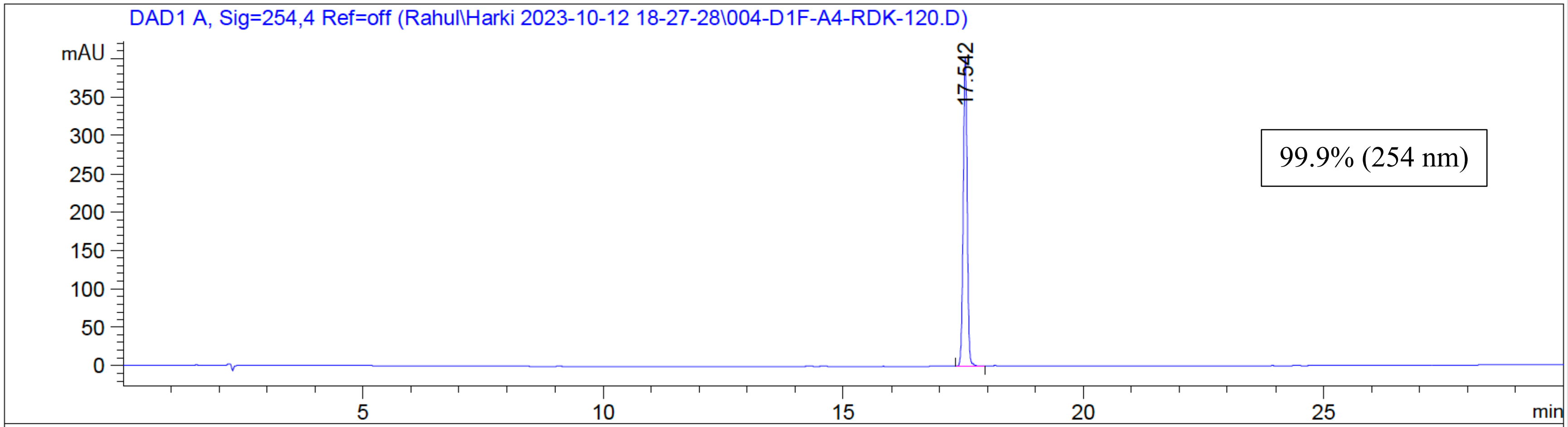

DAD1 A, Sig=254,4 Ref=off (Rahul\Harki 2023-10-12 18-27-28\004-D1F-A4-RDK-120.D)
mAU
350
300
250
200
150
100
50
0
17.542
5
10
15
20
25
min
99.9% (254 nm)

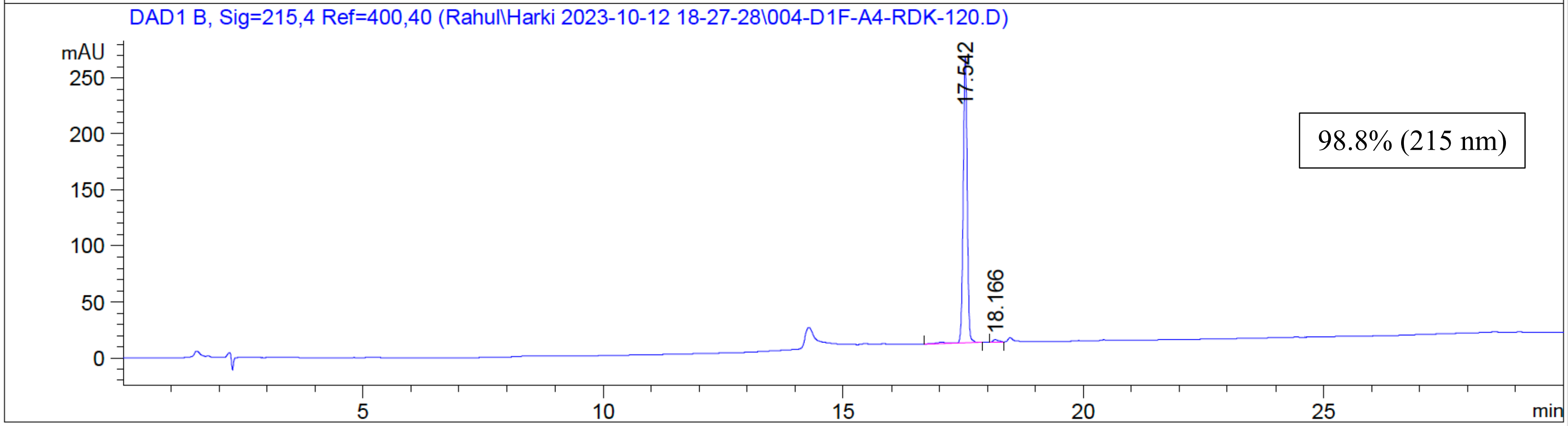

DAD1 B, Sig=215,4 Ref=400,40 (Rahul\Harki 2023-10-12 18-27-28\004-D1F-A4-RDK-120.D)
mAU
250
200
150
100
50
0
17.542
18.166
5
10
15
20
25
min
98.8% (215 nm)

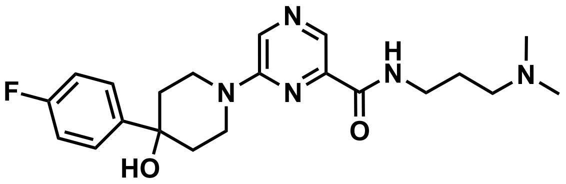
F
HO
N
N
N
H
N
O
N

MWAC-2124

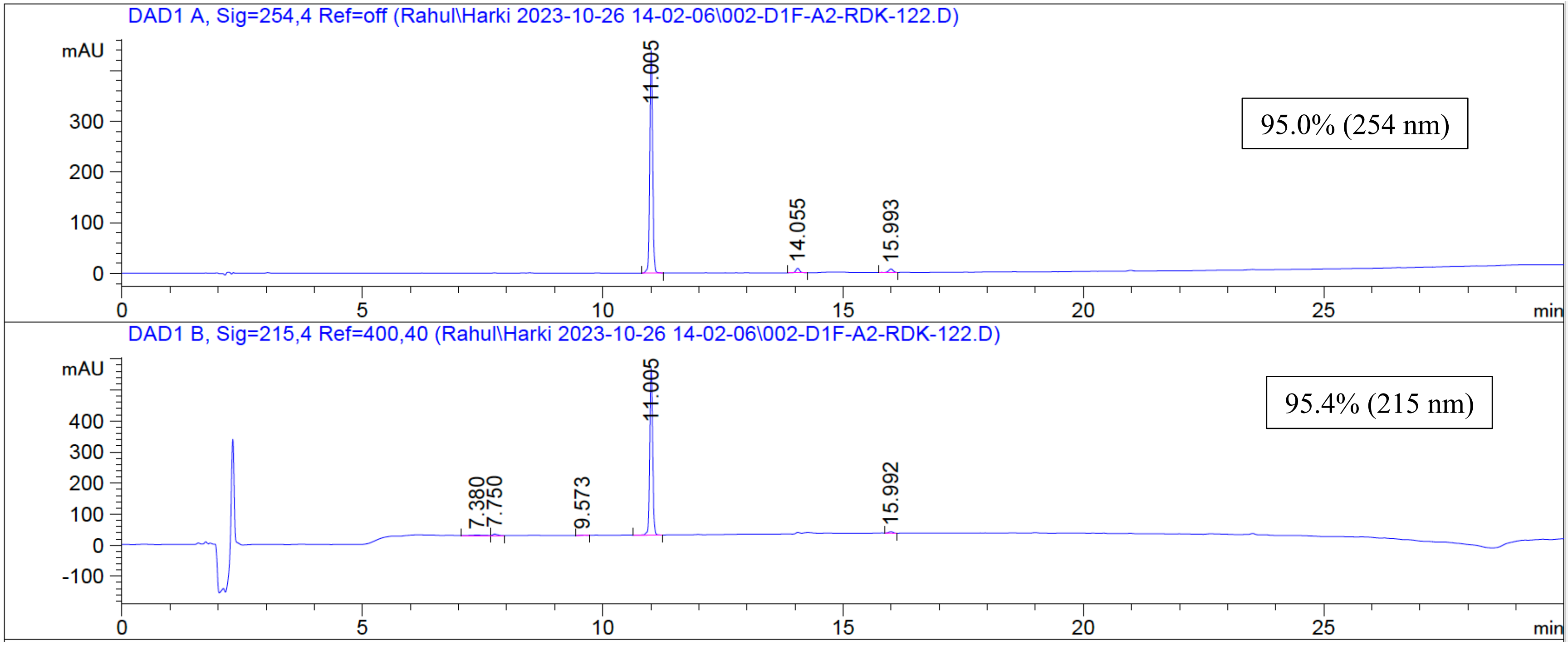
DAD1 A, Sig=254,4 Ref=off (Rahul\Harki 2023-10-26 14-02-06\002-D1F-A2-RDK-122.D)
mAU
300
200
100
0
11.005
14.055
15.993
95.0% (254 nm)
0
5
10
15
20
25
min
DAD1 B, Sig=215,4 Ref=400,40 (Rahul\Harki 2023-10-26 14-02-06\002-D1F-A2-RDK-122.D)
mAU
400
300
200
100
0
-100
7.380
7.750
9.573
11.005
15.992
95.4% (215 nm)
0
5
10
15
20
25
min

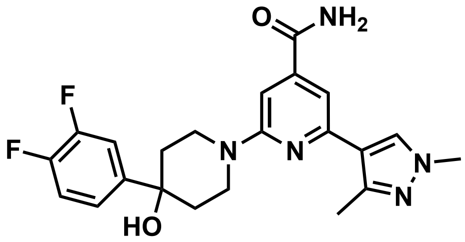
O
NH2
F
F
N
N
N
N
HO

MWAC-2515

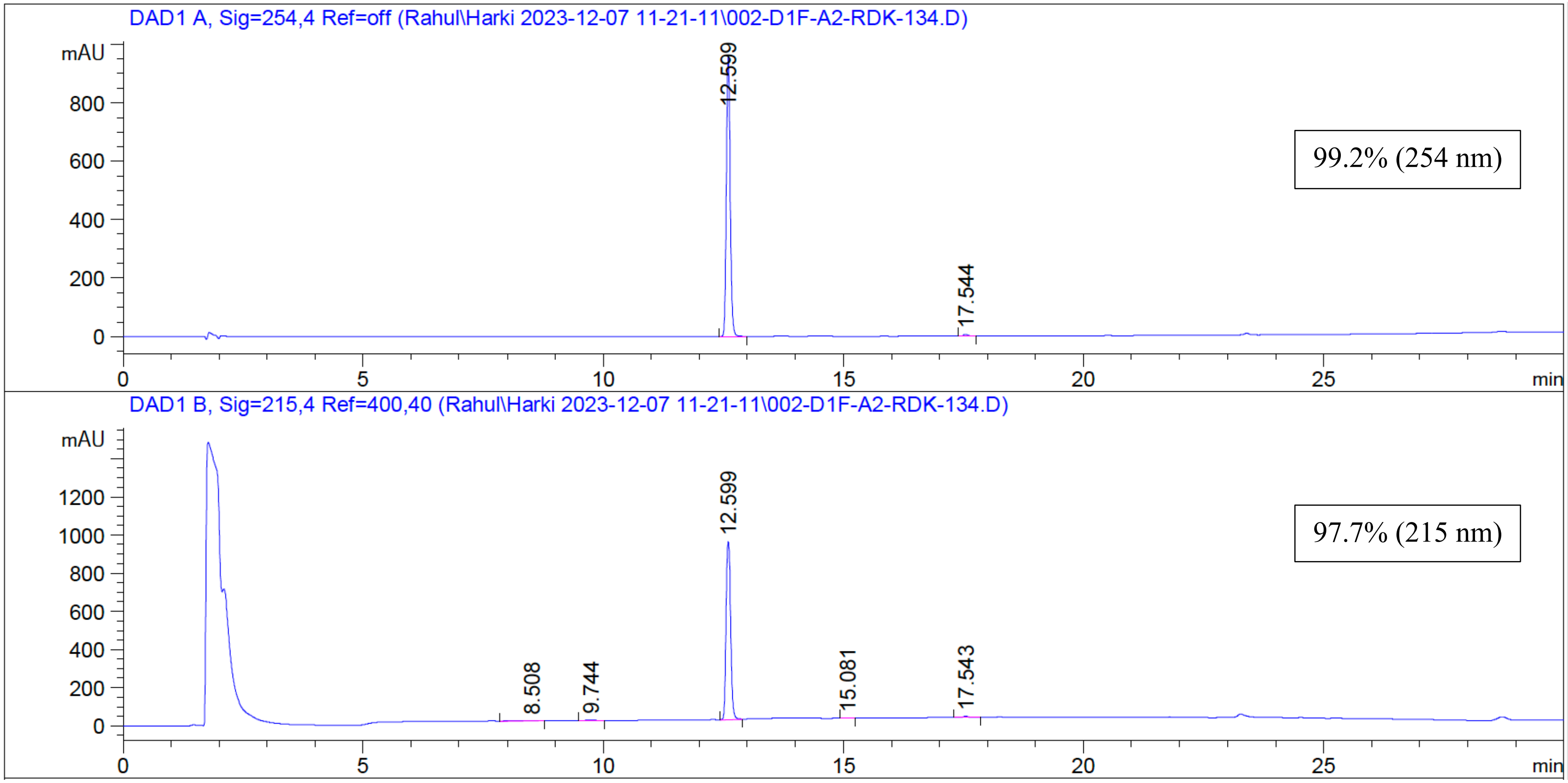
DAD1 A, Sig=254,4 Ref=off (Rahul\Harki 2023-12-07 11-21-11\002-D1F-A2-RDK-134.D)
mAU
800
600
400
200
0
12.599
17.544
99.2% (254 nm)
0
5
10
15
20
25
min
DAD1 B, Sig=215,4 Ref=400,40 (Rahul\Harki 2023-12-07 11-21-11\002-D1F-A2-RDK-134.D)
mAU
1200
1000
800
600
400
200
0
8.508
9.744
12.599
15.081
17.543
97.7% (215 nm)
0
5
10
15
20
25
min

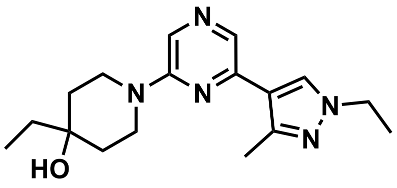
N
N
N
HO
N
N

MWAC-2782

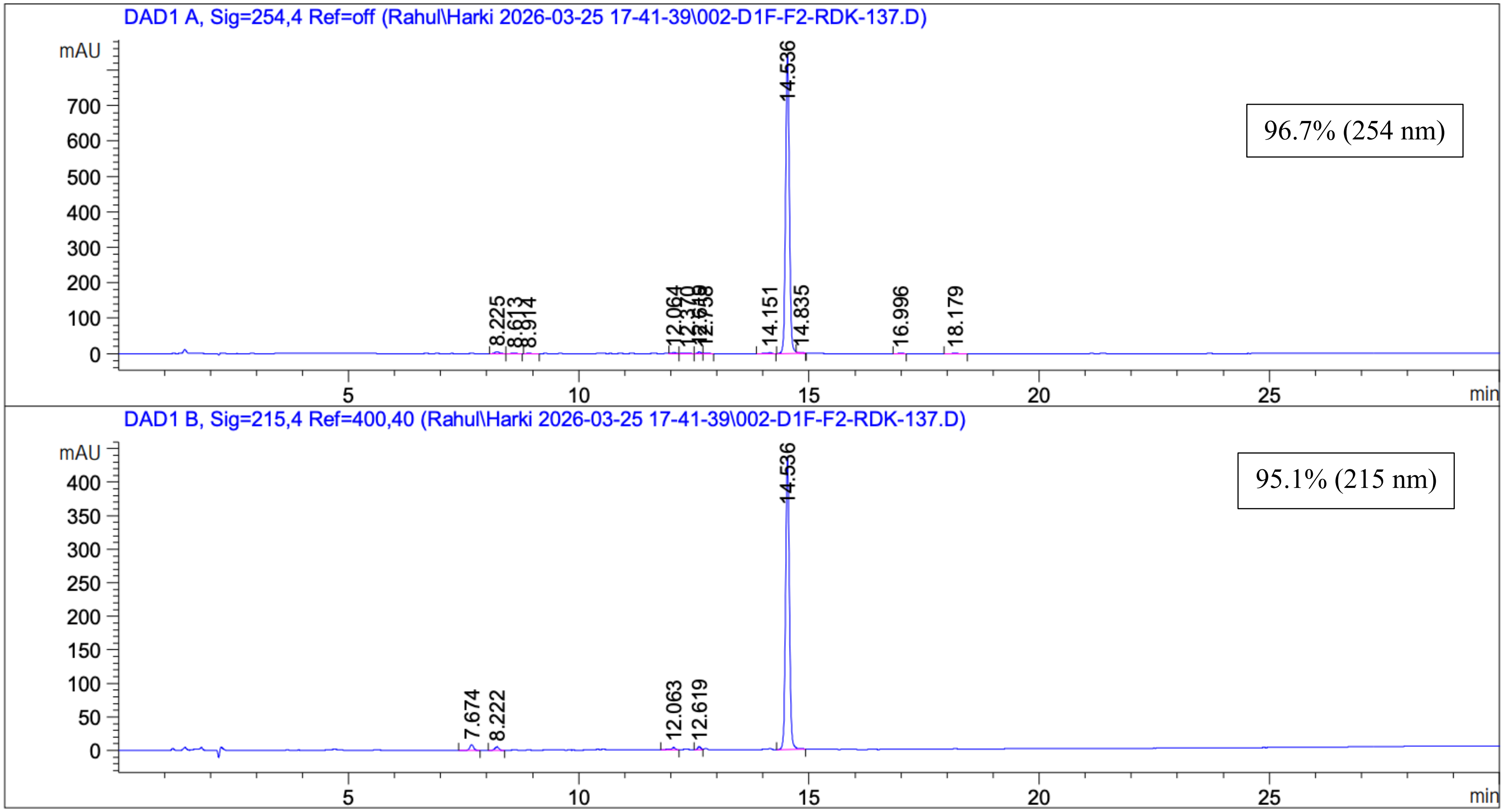
DAD1 A, Sig=254,4 Ref=off (Rahul\Harki 2026-03-25 17-41-39\002-D1F-F2-RDK-137.D)
mAU
700
600
500
400
300
200
100
0
14.536
8.225
8.613
8.914
12.064
14.151
14.835
16.996
18.179
5
10
15
20
25
min
96.7% (254 nm)
DAD1 B, Sig=215,4 Ref=400,40 (Rahul\Harki 2026-03-25 17-41-39\002-D1F-F2-RDK-137.D)
mAU
400
350
300
250
200
150
100
50
0
14.536
7.674
8.222
12.063
12.619
5
10
15
20
25
min
95.1% (215 nm)

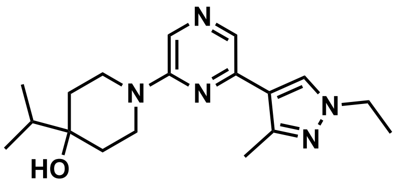

MWAC-2784

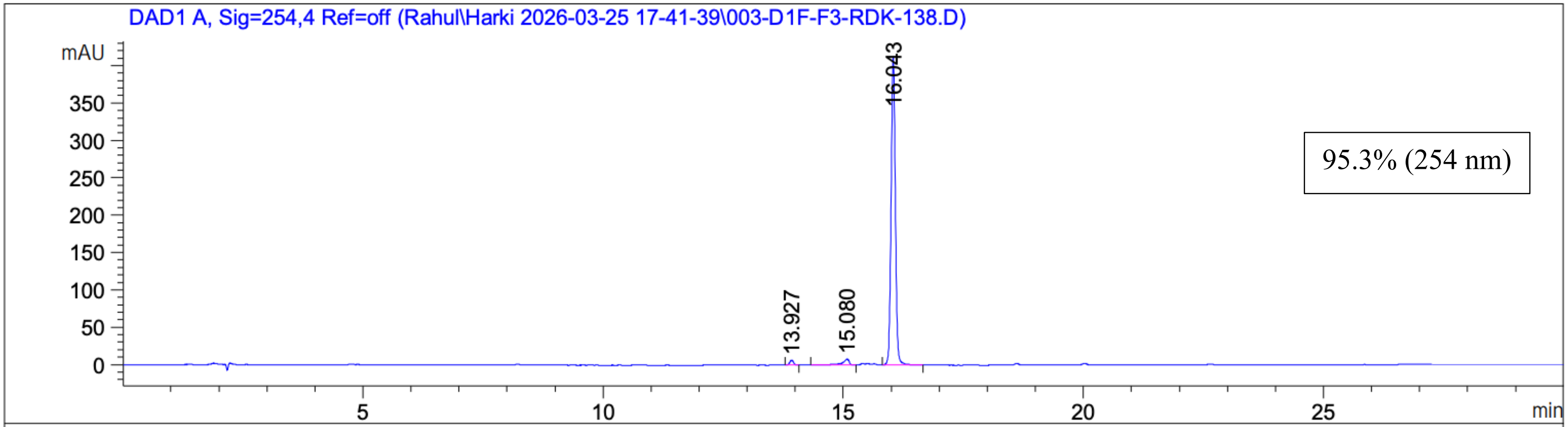
DAD1 A, Sig=254,4 Ref=off (Rahul\Harki 2026-03-25 17-41-39\003-D1F-F3-RDK-138.D)
mAU
13.927
15.080
16.043
95.3% (254 nm)
min

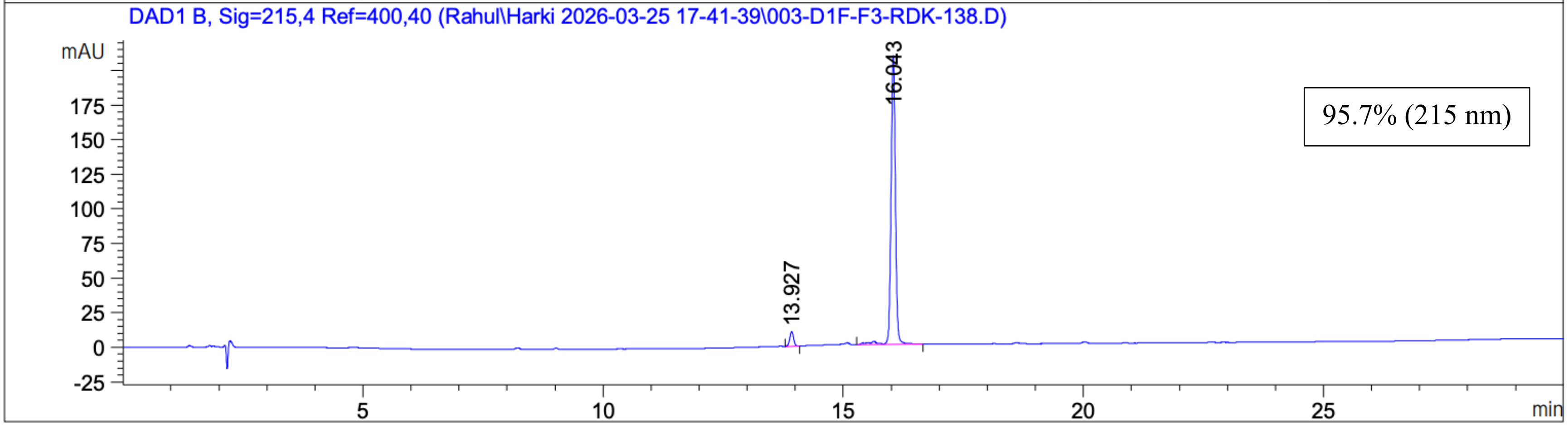
DAD1 B, Sig=215,4 Ref=400,40 (Rahul\Harki 2026-03-25 17-41-39\003-D1F-F3-RDK-138.D)
mAU
13.927
16.043
95.7% (215 nm)
min

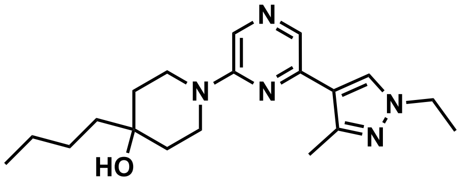
N
N
N
HO
N
N

MWAC-2785

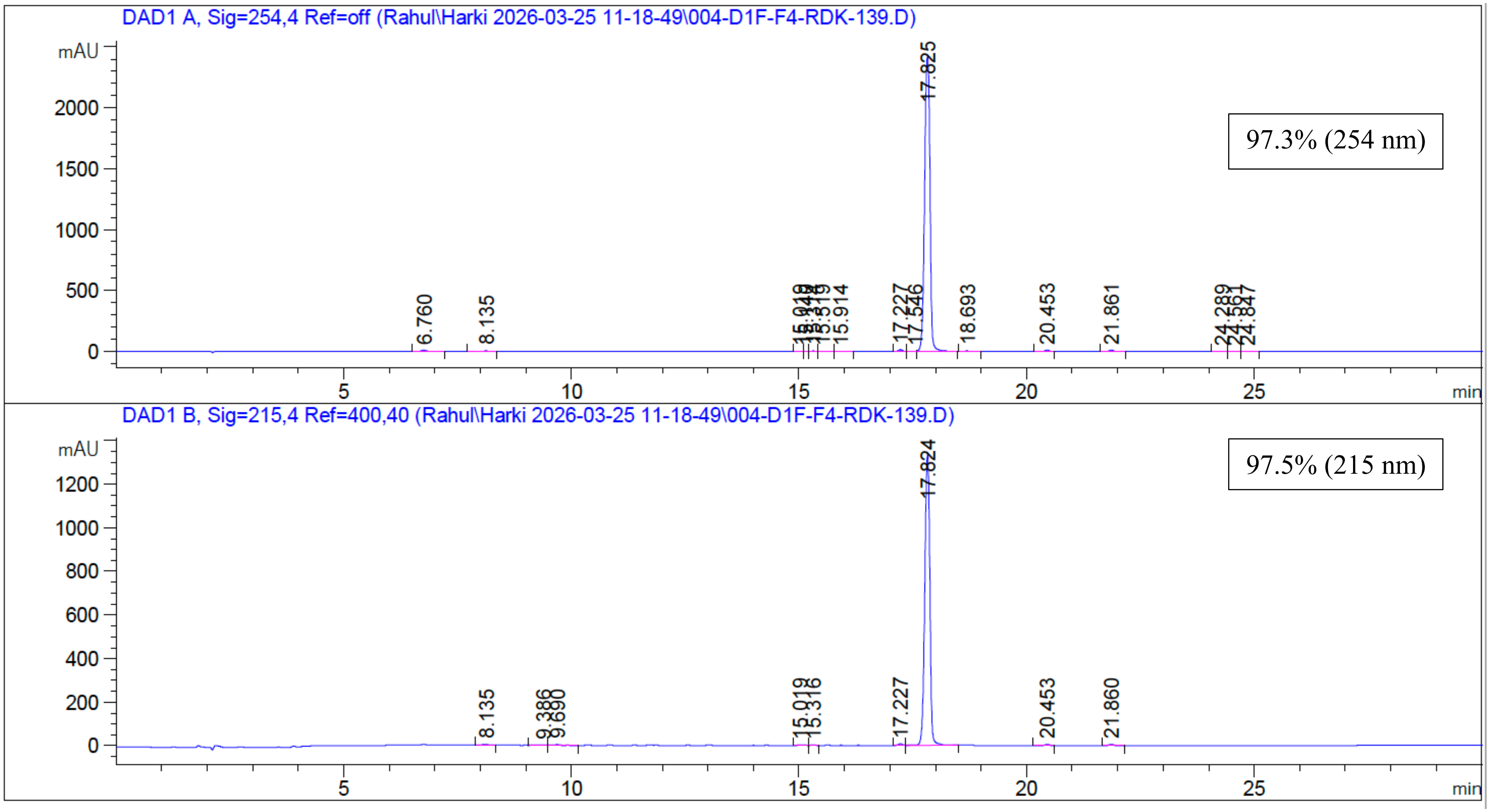
DAD1 A, Sig=254,4 Ref=off (Rahul\Harki 2026-03-25 11-18-49\004-D1F-F4-RDK-139.D)
mAU
2000
1500
1000
500
0
6.760
8.135
15.914
17.227
17.825
18.693
20.453
21.861
24.289
97.3% (254 nm)
5
10
15
20
25
min
DAD1 B, Sig=215,4 Ref=400,40 (Rahul\Harki 2026-03-25 11-18-49\004-D1F-F4-RDK-139.D)
mAU
1200
1000
800
600
400
200
0
8.135
9.386
9.690
15.019
15.316
17.227
17.824
20.453
21.860
97.5% (215 nm)
5
10
15
20
25
min

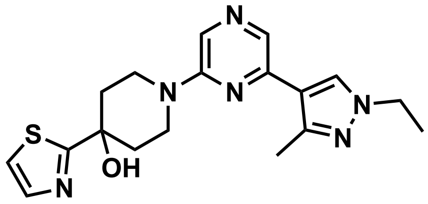

MWAC-3089

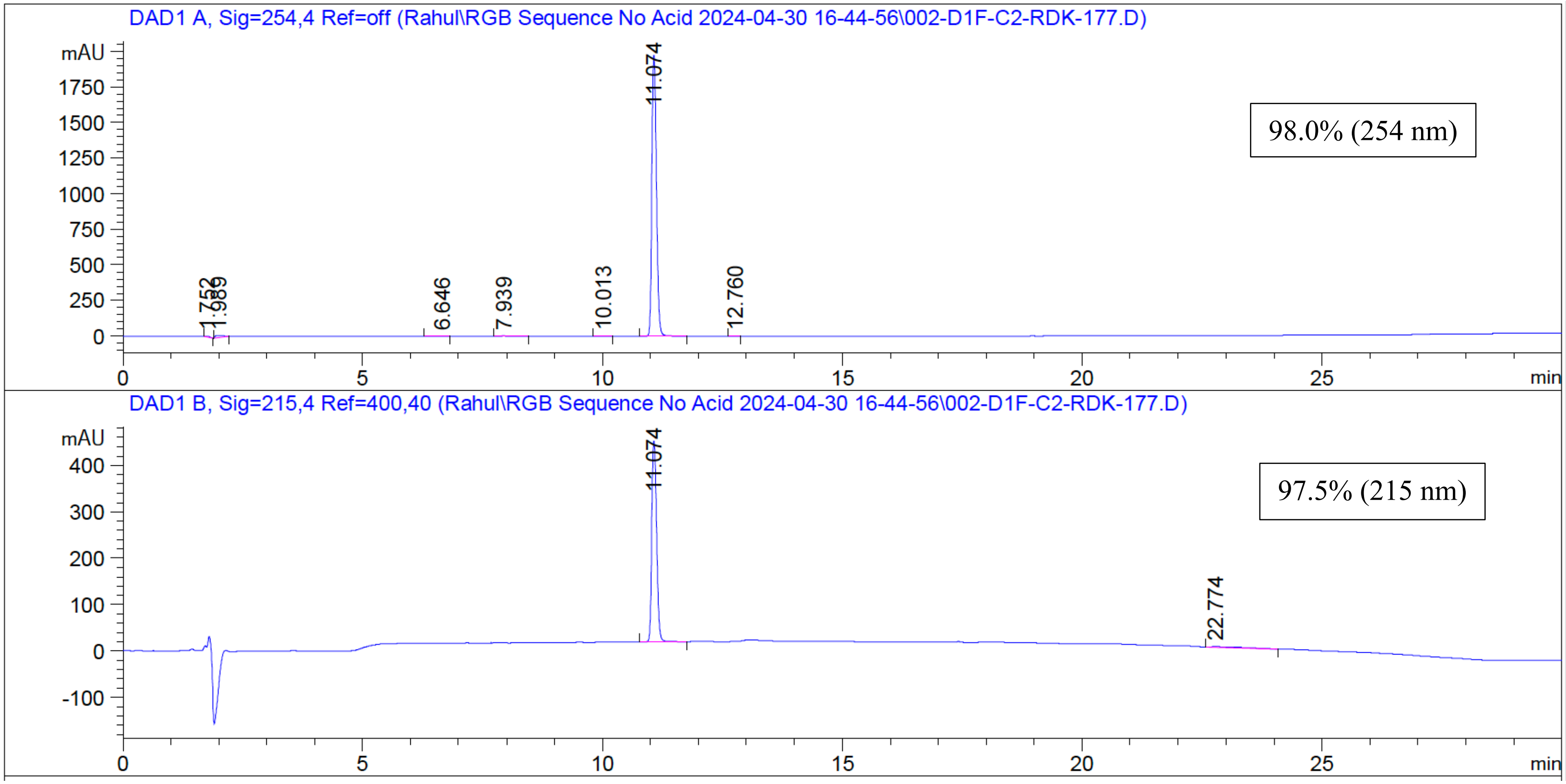
DAD1 A, Sig=254,4 Ref=off (Rahul\RGB Sequence No Acid 2024-04-30 16-44-56\002-D1F-C2-RDK-177.D)
98.0% (254 nm)
DAD1 B, Sig=215,4 Ref=400,40 (Rahul\RGB Sequence No Acid 2024-04-30 16-44-56\002-D1F-C2-RDK-177.D)
97.5% (215 nm)
mAU
min
11.074
22.774

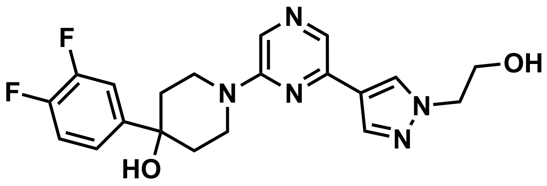

MWAC-3639

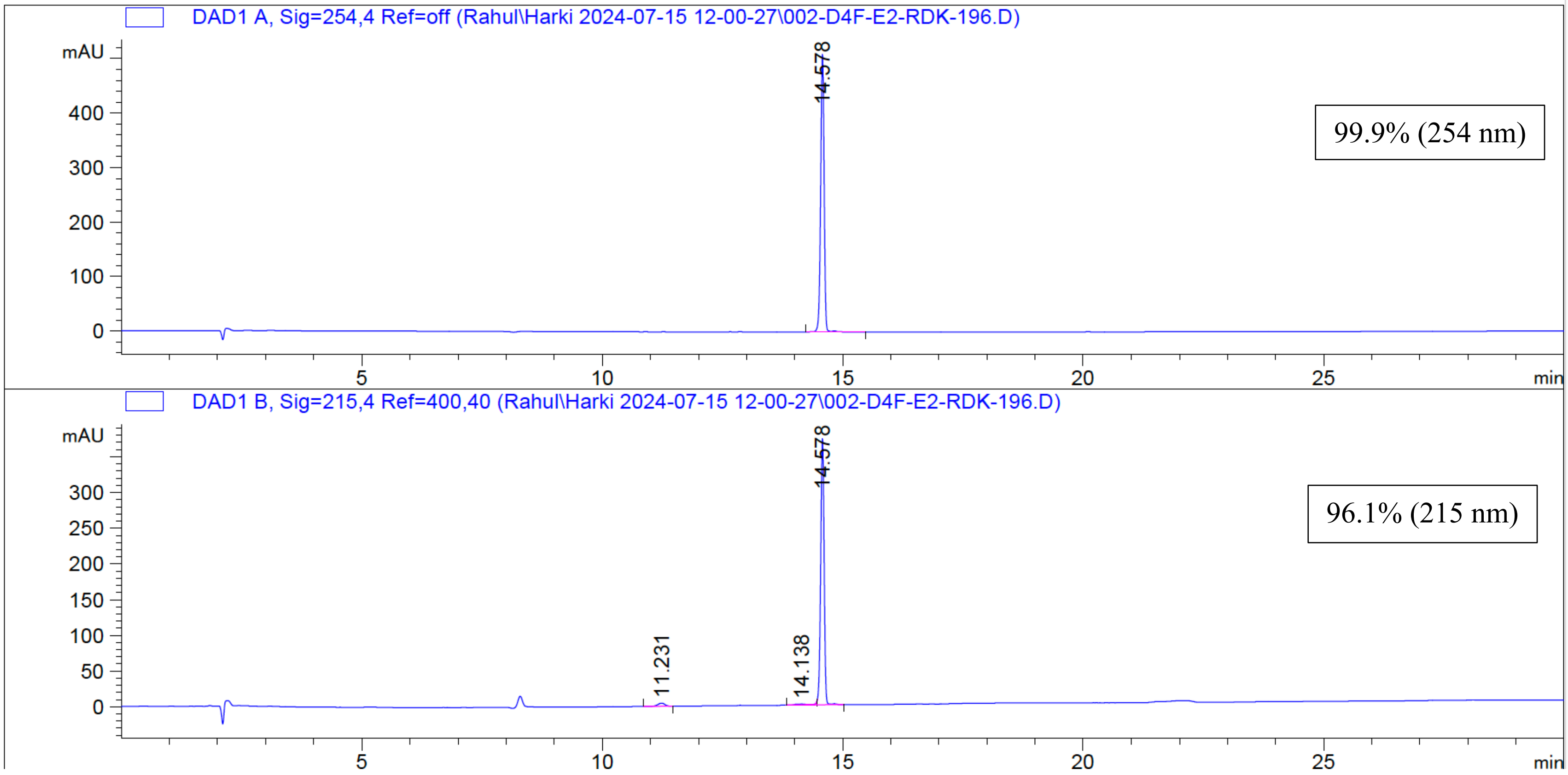
DAD1 A, Sig=254,4 Ref=off (Rahul\Harki 2024-07-15 12-00-27\002-D4F-E2-RDK-196.D)
mAU
14.578
99.9% (254 nm)
min
DAD1 B, Sig=215,4 Ref=400,40 (Rahul\Harki 2024-07-15 12-00-27\002-D4F-E2-RDK-196.D)
mAU
11.231
14.138
14.578
96.1% (215 nm)
min

Reference:
1. Khanna, A., Wilson, J.E. & Zablocki, M.M.; Modulators of TREX1, WO 2020/223590 A1 (2020).
