# Supplementary material for: Selective targeting of human TREX1 exonuclease by small molecule inhibitors is mediated by a conformational switch
Source: NAR Mol Med. 2026 Jun 19;3(3):ugag033. doi: 10.1093/narmme/ugag033 (PMC13335479; doi:10.1093/narmme/ugag033)
Supplement: ugag033_Supplemental_Files [file ugag033_supplemental_files.zip › TREX1_SupplementaryFigs_v18.pdf]

Supplementary Figures

Selective targeting of human TREX1 exonuclease by small molecule inhibitors is mediated by a conformational switch

Patricia C. Hernandez[1,2,3], Rahul Kardile[2,3,4], Ke Shi[1,2,3], Nicholas H. Moeller[1,2,3], Joseph A. Rollie[1,2,3], Daniel A. Harki[2,3,4,*], Hideki Aihara[1,2,3,*]

[1] Department of Biochemistry, Molecular Biology and Biophysics, University of Minnesota, Minneapolis, Minnesota, USA, 55455
[2] Institute for Molecular Virology, University of Minnesota, Minneapolis, Minnesota, USA, 55455
[3] Masonic Cancer Center, University of Minnesota, Minneapolis, Minnesota, USA, 55455
[4] Department of Medicinal Chemistry, University of Minnesota, Minneapolis, Minnesota, USA, 55455
* Corresponding authors: daharki@umn.edu, aihar001@umn.edu

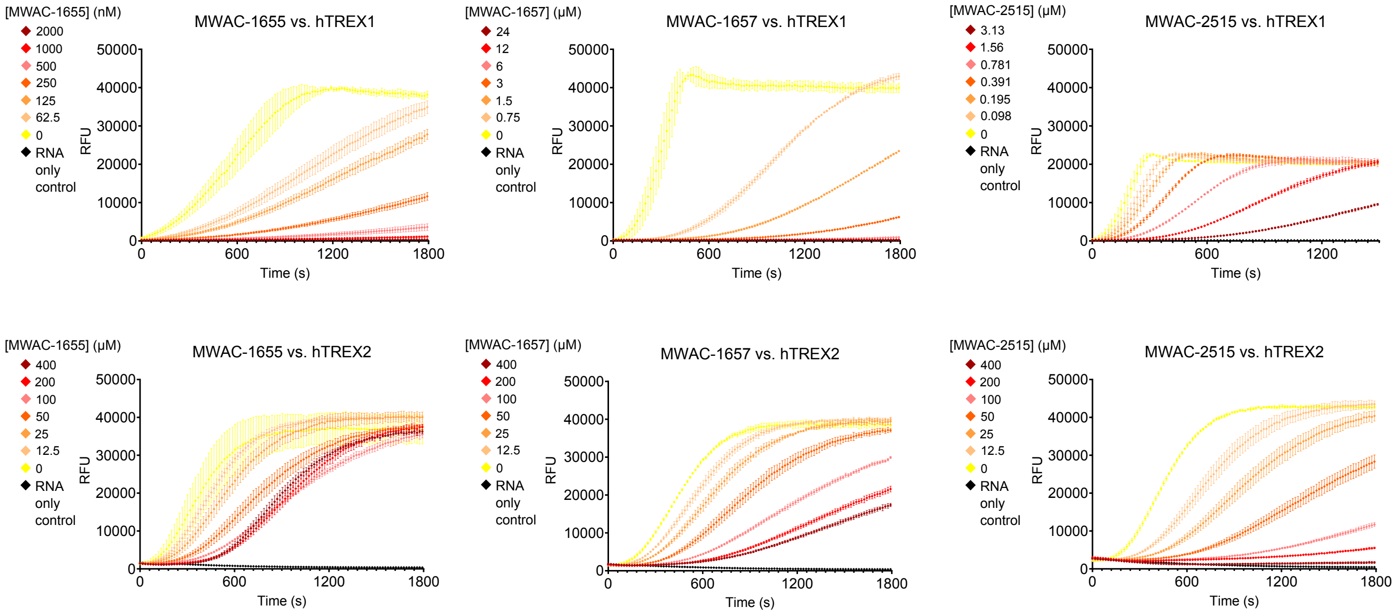

Supplementary Fig. 1. Raw kinetic traces from a real-time fluorescence-based exonuclease assay for hTREX1 and hTREX2.

Representative data showing the increase of fluorescence intensity over time for hTREX1 (top row) and hTREX2 (bottom row), in the presence of the indicated concentration of each inhibitor. The maximum slopes of these traces were determined using ICEKAT (1) and plotted against the inhibitor concentration to obtain the IC_{50} values (Figs. 2 and 3). The results plotted are the average of experiments performed in triplicate, except N=2 for MWAC-1657 against hTREX1, where the third replicate exhibited a lower end-point fluorescence intensity due to the use of a different batch of Cy3-labeled DNA (as seen for MWAC-2515 against hTREX1) and therefore is not plotted together. The error bars show standard deviations.

```
                                  L24
TREX1      MGSQALPPGPMQTLIFFDMEATGLPFSQPKVTELCLLAVHRCALESPPTSQGPPPTVPPP 60
TREX2      ----MSEAPRAETFVFLDLEATGLPSVEPEIAELSLFAVHRSSLENPEHDESGA---LVL 53
                      :*::*:*:******  :*:::**.*:****.:**.*  .:.
                             L15      S22/V23/E24

                              A80 I84
TREX1      PRVVDKLSLCVAPGKACSPAASEITGLSTAVLAAHGRQCFDDNLANLLLAFLRRQPQPWC 120
TREX2      PRVLDKLTLCMCPERPFTAKASEITGLSSEGLARCRKAGFDGAVVRTLQAFLSRQAGPIC 113
           ***:***:**:.* :  :  ********:  **   :  **. :.. * *** **  * *
                              K73

                Y129
TREX1      LVAHNGDRYDFPLLQAELAMLGLTSALDGAFCVDSITALKALERASSPSE-HGPRKSYSL 179
TREX2      LVAHNGFDYDFPLLCAELRRLGARLP-RDTVCLDTLPALRGLDRAHSHGTRARGRQGYSL 172
           ******  ****** ***  **      .:.*:*:: **:.*:** * .     *:.***
                  D121

TREX1      GSIYTRLYGQSPPDSHTAEGDVLALLSICQWRPQALLRWVDAHARPFGTIRPMYGVTASA 239
TREX2      GSLFHRYFRAEPSAAHSAEGDVHTLLLIFLHRAAELLAWADEQARGWAHIEPMYLPPDDP 232
           **:: * :  .*  :*:***** :** *   *   ** *.* :** :. *.***    .

TREX1      RTKPRPSAVTTTAHLATTRNTSPSLGESRGTKDLPPVKDPGALSREGLLAPLGLLAILTL 299
TREX2      SLEA-------------------------------------------------------- 236
             :

TREX1      AVATLYGLSLATPGE 314
TREX2      --------------- 236
```

Supplementary Fig. 2. Amino acid sequence alignment between human TREX1 and TREX2.
The sequence alignment was performed by Clustal Omega (2). The DEDDh motif is highlighted in yellow. TREX1 residues important in inhibitor binding are indicated by red triangles. TREX2 residues that may be responsible for weaker inhibition are also indicated.

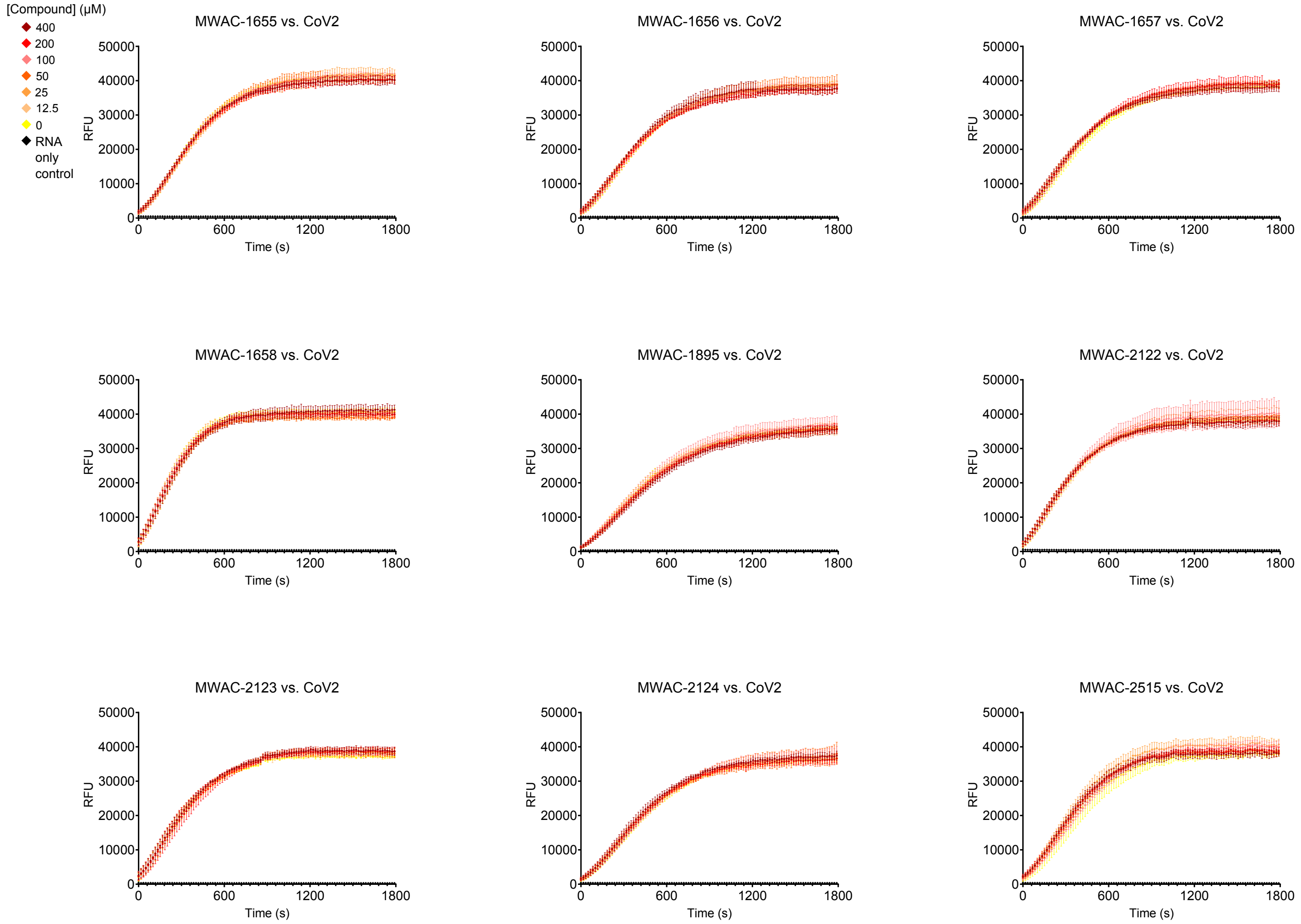

Supplementary Fig. 3. Lack of inhibition of SARS-CoV-2 nsp14/nsp10 exoribonuclease by the 9 compounds in Fig. 1. Raw kinetic traces from the fluorescence-based real-time exoribonuclease assay in the presence of varying concentrations of the compounds are shown. The results are representative of experiments performed in triplicate. The error bars show standard deviations.

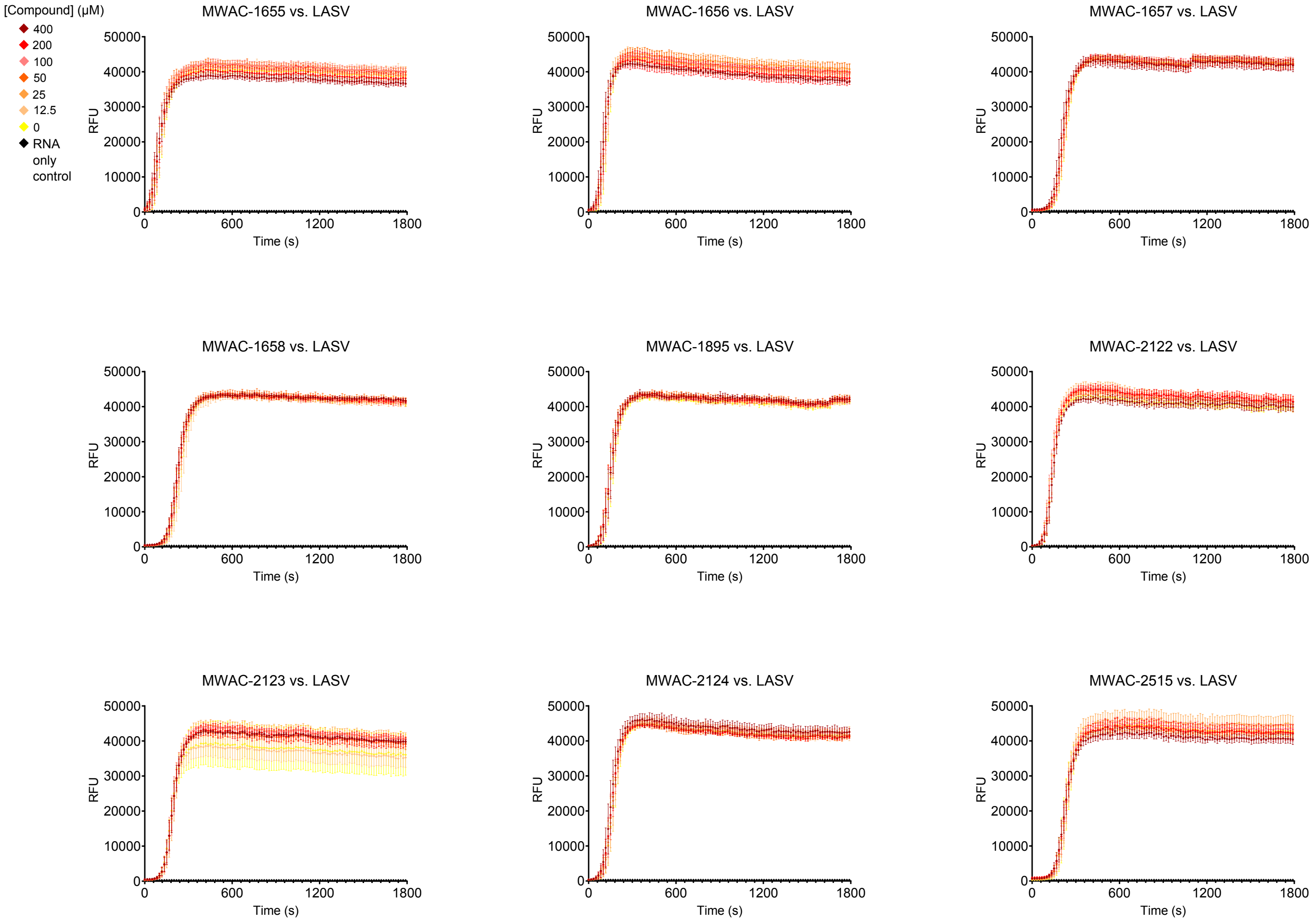

Supplementary Fig. 4. Lack of inhibition of Lassa virus NP exoribonuclease by the 9 compounds in Fig. 1. Raw kinetic traces from the fluorescence-based real-time exoribonuclease assay in the presence of varying concentrations of the compounds are shown. The results are representative of experiments performed in triplicate. The error bars show standard deviations.

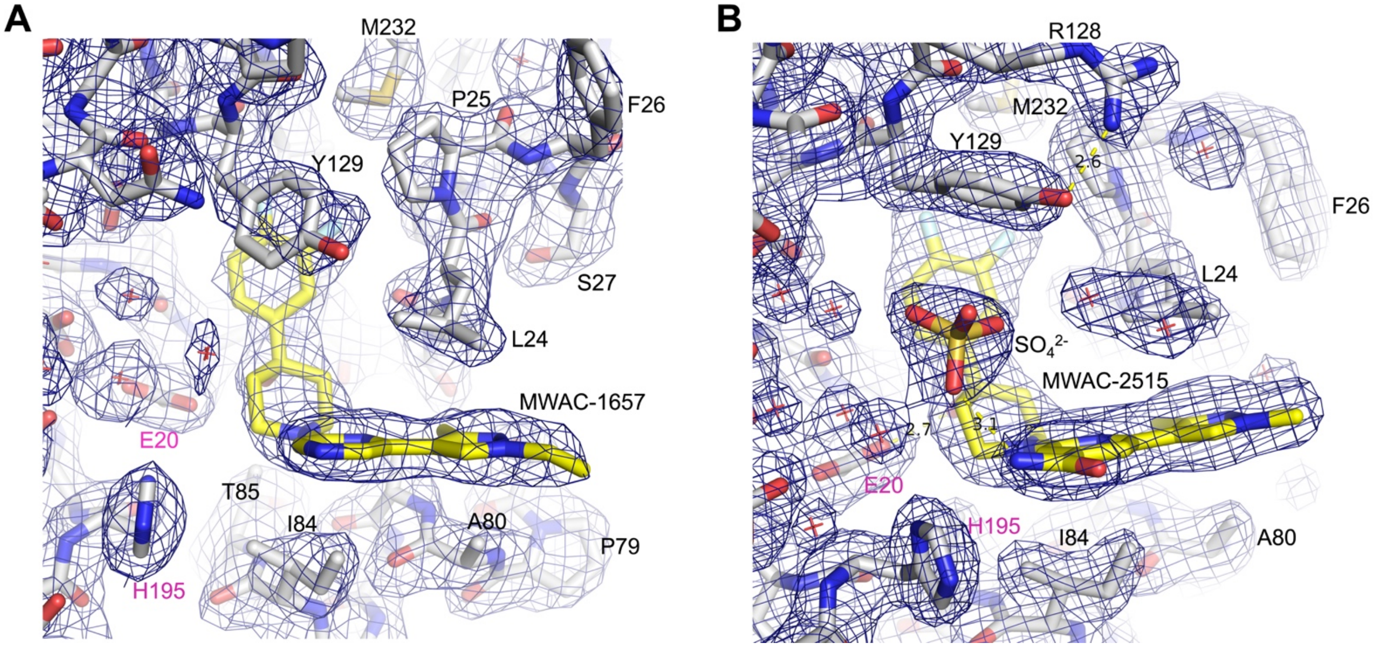

Supplementary Fig. 5. Crystallographic data. The 2Fo-Fc electron density map contoured at 1.0σ for hTREX1 in complex with MWAC-1657 (**A**) or MWAC-2515 (**B**).

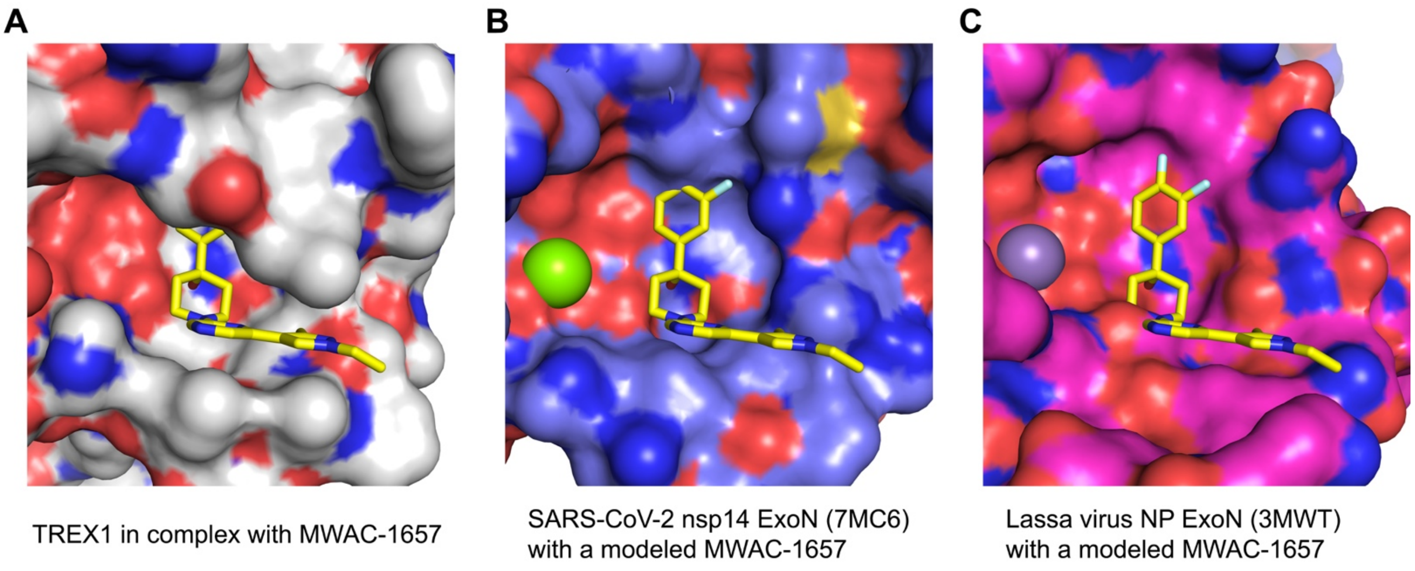

Supplementary Fig. 6. Structural basis for the lack of inhibition of viral ExoN activities by the hTREX1 inhibitors. A, Molecular surface of hTREX1 with bound MWAC-1657. **B,C,** Hypothetical placement of MWAC-1657 in the SARS-CoV-2 nsp14(3)(**B**) or Lassa virus NP(4)(**C**) ExoN active site, generated by superposition of the hTREX1/MWAC-1657 complex on each viral ExoN structure based on the conserved DEDDh residues and removing the hTREX1 structure. The viral ExoNs lack a hydrophobic pocket that accommodates the difluorophenyl moiety of the inhibitor and make a steric clash with the inhibitor, explaining the lack of inhibition.

Supplementary references

1. Bursch, K.L., Olp, M.D. and Smith, B.C. (2023) Analysis of continuous enzyme kinetic data using ICEKAT. *Methods Enzymol*, **690**, 109-129.

2. Madeira, F., Madhusoodanan, N., Lee, J., Eusebi, A., Niewielska, A., Tivey, A.R.N., Lopez, R. and Butcher, S. (2024) The EMBL-EBI Job Dispatcher sequence analysis tools framework in 2024. *Nucleic Acids Res*, **52**, W521-W525.

3. Moeller, N.H., Shi, K., Demir, O., Belica, C., Banerjee, S., Yin, L., Durfee, C., Amaro, R.E. and Aihara, H. (2022) Structure and dynamics of SARS-CoV-2 proofreading exoribonuclease ExoN. *Proc Natl Acad Sci U S A*, **119**.

4. Qi, X., Lan, S., Wang, W., Schelde, L.M., Dong, H., Wallat, G.D., Ly, H., Liang, Y. and Dong, C. (2010) Cap binding and immune evasion revealed by Lassa nucleoprotein structure. *Nature*, **468**, 779-783.
